# Non-local detection of coherent Yu–Shiba–Rusinov quantum projections

**DOI:** 10.1038/s41567-025-03109-y

**Published:** 2025-11-26

**Authors:** Khai That Ton, Chang Xu, Ioannis Ioannidis, Lucas Schneider, Thore Posske, Roland Wiesendanger, Dirk K. Morr, Jens Wiebe

**Affiliations:** 1 Department of Physics, University of Hamburg, Hamburg, Germany; 2 Department of Physics, University of Illinois Chicago, Chicago, IL USA; 3 I. Institute for Theoretical Physics, University of Hamburg, Hamburg, Germany; 4 The Hamburg Centre for Ultrafast Imaging, Hamburg, Germany

**Keywords:** Superconducting properties and materials, Quantum mechanics

## Abstract

Probing spatially confined quantum states from afar—a long-sought goal to minimize external interference—has been proposed to be feasible in condensed-matter systems through the coherent projection of the state. This can be achieved by engineering the eigenstates of the electron sea that surrounds the quantum state using cages built atom by atom, the so-called quantum corrals. However, the demonstration of the coherent nature of the projection and manipulation of its quantum composition are still important goals. Here we show this for the coherent projection of a Yu–Shiba–Rusinov quantum state that is induced by a magnetic impurity, using the eigenmodes of corrals on the surface of a superconductor. This enables us to manipulate the particle–hole composition of the projected state by tuning the corral eigenmodes through the Fermi energy. Our results demonstrate a controlled non-local method for the detection of magnet–superconductor hybrid quantum states.

## Abstract (web summary)

Coherently projecting a quantum state may allow it to be probed from a distance. This is now demonstrated for a Yu–Shiba–Rusinov state using a quantum corral.

## Main

**Fig. 1:**
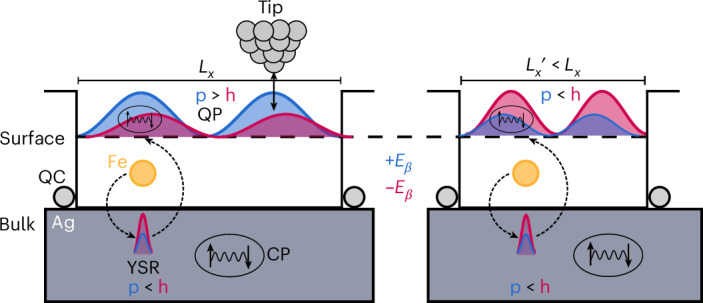
Non-local detection and tuning of p–h composition in a YSR quantum projection. Sketch of the p and h components of the YSR quantum projection (QP; blue and red waves at energies +*E*_*β*_ and −*E*_*β*_, respectively) located at the surface and extended throughout a quantum corral (QC) of Ag atoms (grey spheres) on a proximity SC Ag island with Cooper pairs (CP; grey box). The YSR quantum projection is induced by the p and h components of the YSR state (blue and red maxima at energies +*E*_*β*_ and −*E*_*β*_, respectively) localized in the bulk of the Ag island close to the Fe atom (orange sphere). It is created by a weak, indirect coupling between the YSR state and the quantum corral eigenmode via the proximitized Ag island (dashed arrows), whereas the direct coupling is negligible (see the justification in the main text and Supplementary Note 3). Consequently, the YSR state can be non-locally detected using the STM tip. The p–h composition of the quantum projection can be tuned between inverted (left panel; p > h) with respect to that of the native YSR state (p < h) and non-inverted (right panel; p < h) by adjusting the quantum corral geometry, for example, its lengths (*L*_*x*_ and ${L}_{x}^{{\prime} }$).

Atomic manipulation techniques have provided the unprecedented ability to build atomically precise structures of adatoms^1–3^ or molecules^4^ on the surface of solid-state materials, giving rise to intriguing quantum phenomena ranging from the creation of standing matter waves in quantum corrals^5^ and the design of quantum drums^6^ to the engineering of artificial electronic structures in molecular graphene^7^. Crucial for the emergence of all of these phenomena is the existence of vertically confined, two-dimensional Shockley surface states^8–10^ on the (111) surfaces of noble metals that allow for the multiple and coherent scattering of electrons off the adatom structure. The resulting constructive interference was argued to be the basis for the spatial projection of the many-body Kondo resonance between the foci of an elliptical quantum corral^11^. This effect—called a quantum mirage—was subsequently theoretically shown to be tied to the realization of the particle-in-a-box problem in quantum corrals with tight walls, exhibiting electronic states—corral eigenmodes—with well-defined energies and two-dimensional spatial patterns^12–19^. Indeed, it was theoretically predicted that these eigenmodes could enable the projection of other atomic-scale quantum phenomena, such as the impurity-induced Yu–Shiba–Rusinov (YSR) state in a superconductor (SC)^20^, resonant impurity states in topological insulators^21^, or vibrational^22^ and spin excitations^23^ of atoms, opening a unique opportunity to non-locally probe quantum states at a distance via their projected images. This remote detection, in turn, requires that the projected image reflects and maintains the fundamental quantum nature of the original state. How the quantum nature of the projection can be experimentally detected has remained an intriguing question. Here we demonstrate the quantum nature of a mirage by considering a magnetic-impurity-induced and strongly localized YSR state in an *s*-wave SC^24,25^. In particular, we show the formation of a hybrid quantum state between the YSR state and a quantum corral eigenmode, with a well-defined phase relation between the spatial oscillations of its particle (p)-like and hole (h)-like components, similar to that found in long-range coherent YSR states^24,26,27^. In the following, we refer to this hybrid quantum state, which was theoretically predicted 20 years ago^20^, as a coherent quantum projection of the YSR state. To realize this quantum projection, we place single magnetic Fe atoms in Ag-atom-based quantum corrals assembled on the (111) surface of thin proximity SC Ag islands grown on Nb(110), making use of their energetically and spatially well-defined eigenmodes (Fig. 1). As we will show below, the Fe spin is coupling predominantly to the bulk Ag states (rather than to the surface state), inducing a YSR state at energy ±*E*_*β*_ deep inside the bulk SC gap. The YSR state, in turn, couples to the surface states, leading to a spectroscopic projection via the Machida–Shibata states (MSSs), which are the corral eigenmodes with a proximity-induced SC gap that enter the substrate gap whenever a corral eigenmode is close to *E*_F_ (refs. ^24,28,29^). The YSR state and its projection are energetically well separated from the de Gennes–Saint James coherence states of the substrate^30^ as well as from the MSSs. We demonstrate that an image of the YSR state can be coherently projected over length scales up to 20 times larger than its localization length, where it is non-locally detected with the scanning tunnelling microscopy (STM) tip in a minimally perturbative fashion. Over these distances, the p- and h-like components of the YSR projection maintain their spatial phase shift, reflecting one of the hallmarks of the long-range coherent YSR quantum state^24,26,27^. This projection occurs whenever the Fe adatom is located close to a maximum in the corral eigenmode’s wavefunction, even when this maximum is not located close to the focus of an elliptical quantum corral. By adjusting the corral length *L*_*x*_ and thereby tuning the corral’s eigenmodes through *E*_F_, we can invert the p–h composition of the long-range part of the projected YSR state located at the surface of the Ag island but extended throughout the quantum corral having the shape of the corral eigenmode (Fig. 1).

## Native YSR states of Fe atoms

**Fig. 2:**
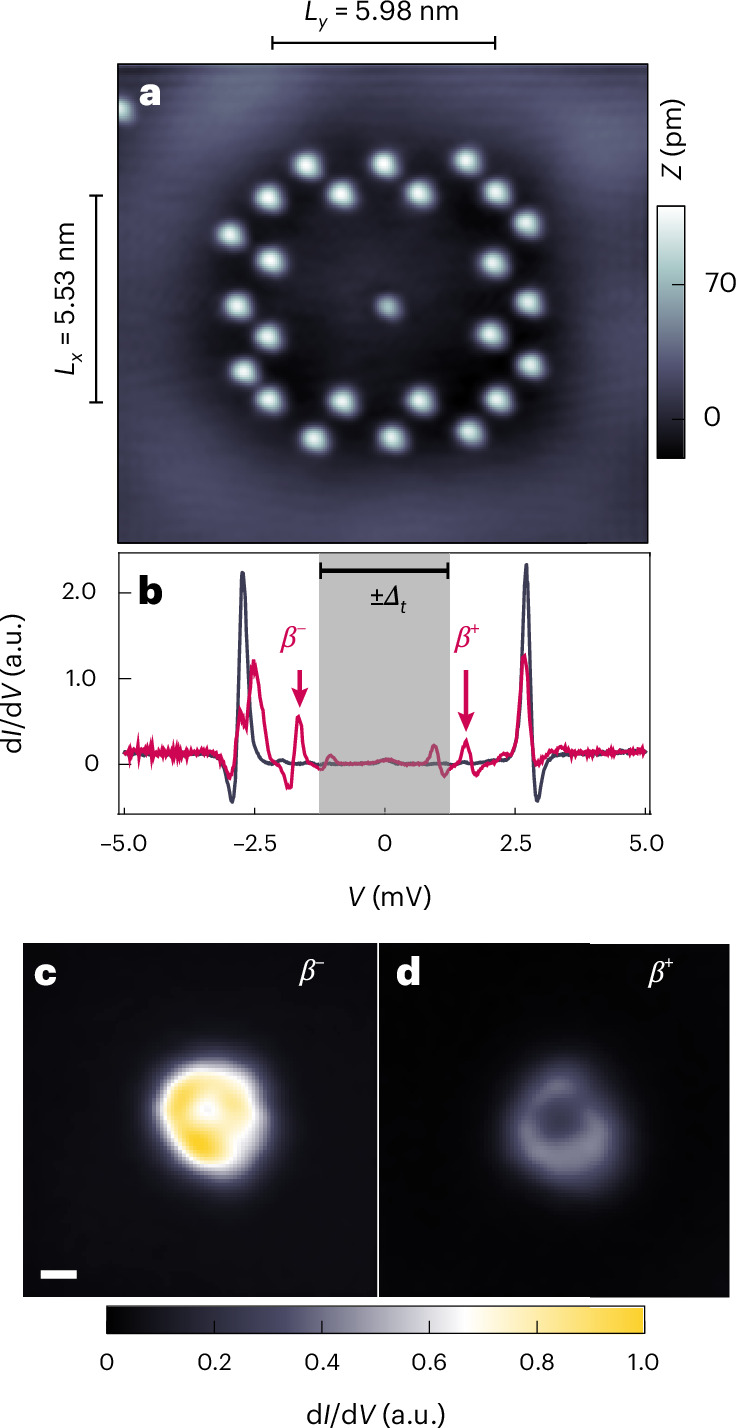
Native YSR states of the Fe atom. **a**, Constant-current STM image of a single Fe atom in the centre of a Ag corral of dimensions *L*_*x*_ = 5.53 nm and *L*_*y*_ = 5.98 nm (*V*_bias_ = 5 mV, *I*_set_ = 1 nA). *L*_*x*_ and *L*_*y*_ are defined as the distances between the inner rows of Ag atoms. **b**, d*I*/d*V* spectra taken on the Fe atom (red) shown in **a** and a substrate region (black) inside an even smaller corral without an Fe atom (Supplementary Fig. 2; *V*_stab_ = 5 mV, *I*_stab_ = 1 nA; ${V}_{{\rm{mod}}}=50\,\upmu {\rm{V}}$ for the Fe atom and ${V}_{{\rm{mod}}}=20\,\upmu {\rm{V}}$ for the substrate). **c**,**d**, Constant-contour d*I*/d*V* maps of the Fe atom shown in **a** acquired at a bias voltage *V*_bias_ of −1.61 mV (**c**) and 1.61 mV (**d**) corresponding to the energies ∓*E*_*β*_ of the h (*β*^−^) and p (*β*^+^) components, respectively, of the *β* YSR state (*V*_stab_ = 5 mV, *I*_stab_ = 1 nA, ${V}_{{\rm{mod}}}=100\,\upmu {\rm{V}}$). Scale bar, 0.3 nm (**c**). a.u. is the output voltage (in volts) of the lock-in amplifier using the same lock-in parameters for both d*I*/d*V* maps.

We start with the investigation of the native YSR states of single Fe atoms on the surface of a Ag island of thickness 12 nm grown on the (110) surface of an SC Nb single crystal^29,31^ (for experimental procedures, see the Methods and Supplementary Note 1). First, to exclude the interaction of the Fe 3*d* orbitals with any surface-state-related mode, we place the Fe atoms inside a nearly square-shaped double-walled quantum corral made out of non-magnetic (Supplementary Note 2) Ag atoms with a sufficiently small length and width of *L*_*x*_ = 5.53 nm and *L*_*y*_ = 5.98 nm, respectively, (Fig. 2a) such that the lowest quantum corral eigenmode has an energy far above *E*_F_ (Supplementary Note 4 and ref. ^29^). Thus, the energy, spatial extension and p–h composition of the native YSR state stemming from the coupling of the Fe 3*d* orbitals to the proximity SC Ag bulk states can be determined. Figure 2b (black curve) shows a Ag substrate spectrum measured in an even smaller empty corral (Supplementary Fig. 2), revealing the de Gennes–Saint James coherence peaks of the Ag island. Note that we used an SC tip with a gap *Δ*_t_ = 1.32 meV for all measurements, such that the sample states appear at a bias voltage *V* shifted by ±*Δ*_t_/*e* away from zero bias with respect to their original energy (Supplementary Note 1 and Supplementary Fig. 1). Compared with the spectrum taken on the Ag substrate, the spectrum taken on the Fe atom (Fig. 2b, red curve) reveals three pairs of in-gap peaks (Supplementary Note 2 and Supplementary Fig. 3), which we identify as YSR states resulting from the coupling of the Fe 3*d* orbitals to the Ag island^32^. In the following, we focus on the lowest-energy YSR state labelled *β*^±^ (Fig. 2b) with energy ±*E*_*β*_, which is energetically well separated from the de Gennes–Saint James coherence states of the substrate, as well as from the MSSs for any corral size. The other YSR states either overlap with the MSS (*α*^±^), or are too small in intensity (*γ*^±^), such that their YSR quantum projections cannot be properly analysed (Supplementary Figs. 3b, 16c and 18d–f). Constant-contour d*I*/d*V* maps taken at the bias voltage of the *β*^±^ YSR state reveal the spatial shape, extent and p–h composition of this native YSR state (Fig. 2c,d). The h-like (*β*^−^) and p-like (*β*^+^) components possess different spatial forms, resembling downward- and upward-pointing triangles, respectively, reflecting an orbital origin that is distinctly different from that of the other two YSR states (Supplementary Note 2 and Supplementary Fig. 3). Moreover, they have a spatial extent and, hence, a localization length of ~0.75 nm (Supplementary Fig. 3g,h), which is similar to the apparent diameter of the Fe atom extracted from the STM images. Finally, the intensity of the *β*^−^ component is substantially larger than that of the *β*^+^ component. We, thus, conclude that without the coupling to a surface-related mode, the native *β* YSR state of the Fe atom, which originates in the coupling of the Fe 3*d* orbitals to the paired electrons in the bulk of the Ag island, possesses a dominant h-like component and is detectable only up to distances of about 1 nm away from the Fe atom.

## YSR quantum projection in elliptical quantum corrals

**Fig. 3:**
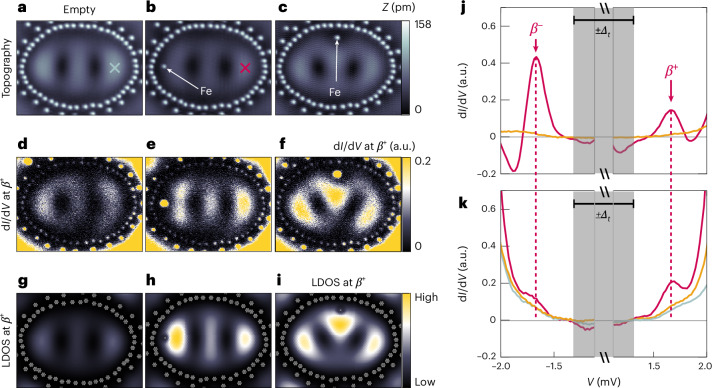
YSR quantum projection in an elliptical quantum corral. **a**–**c**, Constant-current STM images of an empty Ag corral (**a**) with the major-axis length *a* = 18.20 nm and minor-axis length *b* = 13.30 nm, and the same corral with an Fe atom located on the major axis close to the left edge (**b**) and on the minor axis close to the top edge (**c**) (*V*_bias_ = −5 mV, *I*_set_ = 1 nA). **d**–**i**, Experimental (**d**–**f**) constant-height d*I*/d*V* maps and theoretical (**g**–**i**; Supplementary Note 3) LDOS maps of the empty corral (**d** and **g**), the corral with the Fe on the major (**e** and **h**) and on the minor axis (**f** and **i**) taken close to +*E*_*β*_ (**d**–**f**: *V*_bias_ = 1.67 mV, *V*_stab_ = −5 mV, *I*_stab_ = 1 nA, ${V}_{{\rm{mod}}}=100\,\upmu {\rm{V}}$; a.u.: output voltage (in volts) of the lock-in amplifier using the same lock-in parameters for all three d*I*/d*V* maps; **g**–**i**: for the theoretical parameters, see Supplementary Note 3). **d**–**f** have been set to the same ranges in the colour scale. The same applies to **g** and **h**. The colour-scale range in **i** was set to its minimum and maximum values. **j**, d*I*/d*V* spectra taken on the Fe atom in **b** (red) and on a Ag atom replacing the Fe atom (orange; see the corral in Supplementary Fig. 4j,m,n). **k**, d*I*/d*V* spectra taken on the empty locations indicated by the crosses in **a** and **b** of the empty corral (grey), the corral in **b** with the Fe atom (red) and the corral in which the Ag atom replaces the Fe atom (orange) (*V*_stab_ = −5 mV, *I*_stab_ = 1 nA, ${V}_{{\rm{mod}}}=50\,\upmu {\rm{V}}$).

To realize the YSR quantum projection, we first consider an elliptical quantum corral whose wall also consists of two rows of non-magnetic Ag atoms (Fig. 3a). The size of the corral is tailored such that there exists a corral eigenmode close to *E*_F_ whose spatial structure is revealed by the close-to-zero-bias STM image (Fig. 3a) and constant-height d*I*/d*V* map (Fig. 3d) across the corral. Placing an Fe atom inside the corral on the main axis close to the left maximum of the corral eigenmode (Fig. 3b), we find that a *β* YSR state appears in the d*I*/d*V* spectrum measured at the Fe site at the same energies ±*E*_*β*_ and with similar relative intensities between the *β*^−^ and *β*^+^ components as in the native case investigated above (Figs. 3j and 2b). However, the spectroscopic signature of this YSR state is now detectable in the d*I*/d*V* signal at distances more than ten times larger than the spatial extent of the native *β* YSR state, as revealed by a d*I*/d*V* map close to +*E*_*β*_ (Fig. 3e; data close to −*E*_*β*_ are shown in Supplementary Fig. 4e). The spatial pattern of this state strongly resembles that of the empty corral’s eigenmode in Fig. 3d, but it now appears with an increased intensity in Fig. 3e. Moreover, a d*I*/d*V* spectrum taken near the rightmost maximum of this spatially extended state (Fig. 3b, red cross) reveals that it is located at approximately the same energy ±*E*_*β*_ and possesses a similar spectral width as the Fe atom’s native *β* YSR state (Fig. 3k, red spectrum). Note, however, that the p–h composition of this extended state is inverted with regard to the native *β* YSR state, with the p-like component now showing a larger intensity than the h-like component. If the Fe atom is replaced by a non-magnetic Ag atom at the same lattice site (Supplementary Fig. 4j), the d*I*/d*V* spectrum taken at the position of the cross shown in Fig. 3b is largely indistinguishable from that taken on the same site in the empty corral, revealing no signature of the spatially extended in-gap state (Fig. 3k, orange and grey lines). The d*I*/d*V* map shown in Fig. 3e, thus, demonstrates that the proximity of the corral’s eigenmode to *E*_F_ leads to a spatially extended projection of the native Fe *β* YSR state, thereby creating the theoretically predicted YSR quantum mirage^20^. Additional data with the Fe atom on other locations inside the corral (Fig. 3c,f and Supplementary Fig. 4b,c,f–i) reveal that the intensity and spatial form of this induced YSR quantum projection strongly depend on the location of the Fe atom with respect to the maxima in the corral eigenmode, as expected for the projection induced by a quantum state^20^. Moreover, the nearly identical energies of the *β*^±^ YSR peaks measured on the native Fe and in the presence of the corral eigenmode (Figs. 2b and 3j and Supplementary Note 8) suggest a dominant coupling of the Fe 3*d* orbitals to the bulk Ag electronic states, rather than to the Ag surface state. By contrast, a strong coupling to the surface state would lead to a substantial variation in the YSR state’s energy when the size of the corral is changed^20^ (Supplementary Note 3 and Supplementary Fig. 9).

To further elucidate the microscopic origin of the experimental data, we consider a semi-infinite three-dimensional (3D) tight-binding Hamiltonian (Methods and Supplementary Note 3), where we assume that the Fe (Ag) atoms couple to the bulk (surface) states only, as discussed above. The theoretically computed local density of states (LDOS) (Fig. 3g–i) obtained from this Hamiltonian shows good agreement with the experimental d*I*/d*V* maps (Fig. 3d–f) for an empty corral, as well as for the cases in which an Fe atom is located at different positions inside the corral. This substantiates our conclusion that the experimental data can be interpreted as the coherent projection of the native *β* YSR state through the corral’s eigenmode, thereby creating a YSR mirage. This projection occurs via an indirect path involving a dominant coupling of the Fe 3*d* orbitals to the Ag bulk states, which, in turn, are coupled to the surface state, as schematically shown in Fig. 1. We note that the assumption of a static magnetic impurity in our calculations (Supplementary Note 3) implies that the *β*^±^ components of the native YSR state, as well as that of the YSR quantum projection, are fully spin polarized.

## Coherent nature of the YSR quantum projection

**Fig. 4:**
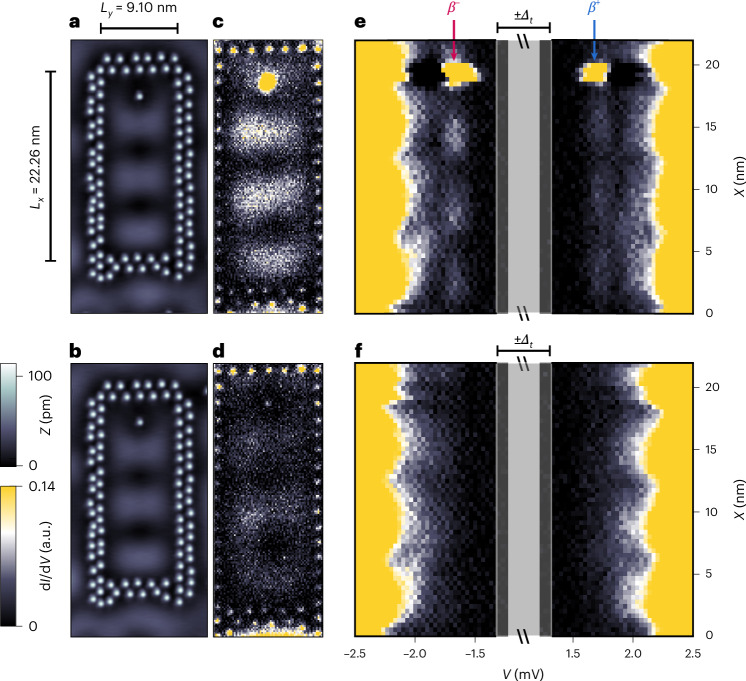
Long-range coherence of the YSR quantum projection. **a**,**b**, Constant-current STM images of a Ag corral (*L*_*x*_ = 22.26 nm, *L*_*y*_ = 9.1 nm) with the Fe atom placed in the topmost quarter (**a**) and of the same corral in which the Fe atom was replaced with a Ag atom (**b**) (Fourier filtered, *V*_bias_ = −5 mV, *I*_set_ = 1 nA). **c**,**d**, Constant-height d*I*/d*V* maps taken close to −*E*_*β*_ inside the corrals of **a** (**c**) and **b** (**d**) (*V*_bias_ = −1.67 mV, *V*_stab_ = −5 mV, *I*_stab_ = 1 nA, ${V}_{{\rm{mod}}}=100\,\upmu {\rm{V}}$). **e**,**f**, d*I*/d*V* line profiles taken along the longitudinal vertical axes through the corrals in **a** (**e**) and **b** (**f**) (*V*_stab_ = −5 mV, *I*_stab_ = 1 nA, ${V}_{{\rm{mod}}}=50\,\upmu {\rm{V}}$). The red and blue arrows above **e** indicate *V*_bias_ corresponding to −*E*_*β*_ and +*E*_*β*_, respectively. The grey vertical lines indicate the bias voltage of the tip gap *Δ*_t_/*e*. a.u.: output voltage (in volts) of the lock-in amplifier using the same lock-in parameters for **c**–**f**.

To demonstrate that a coherent projection of the YSR state can be achieved, we consider a rectangular quantum corral of width *L*_*y*_ = 9.1 nm and length *L*_*x*_ = 22.26 nm, with an Fe atom placed on the corral’s longitudinal axis (Fig. 4a). For such a corral, the eigenmodes are characterized by a pair of quantum numbers (*n*_*x*_, *n*_*y*_) reflecting the numbers of maxima *n*_*x*_ (*n*_*y*_) in the *x* (*y*) direction^29^. For *L*_*x*_ = 22.26 nm, the (3, 1) eigenmode is the one located closest to *E*_F_, as evidenced by the constant-current map shown in Fig. 4a (note a slight downward shift in the eigenmode due to the presence of the Fe atom). The native YSR state of the Fe atom couples to this eigenmode, leading to its spatial projection, as follows from the d*I*/d*V* map taken close to −*E*_*β*_ (Fig. 4c). A d*I*/d*V* line profile taken along the corral’s longitudinal axis (Fig. 4e, Supplementary Note 5 and Supplementary Fig. 18) shows a clear energetical separation of the *β*^±^ components of the YSR quantum projection from the MSSs, with an energy linewidth comparable to that of the native *β* YSR state. It also reveals a constant relative phase shift in the spatial oscillations between the *β*^−^ and *β*^+^ components of the YSR quantum projection (Supplementary Note 7 and Supplementary Fig. 21), which is a characteristic signature of its phase coherence^24,26,27^. In addition, we find that the amplitudes of the spatial oscillations hardly attenuate with distance from the Fe atom, which further substantiates the coherence of the projection of the quantum state. We, thus, conclude that the coupling of the YSR state to the quantum corral eigenmodes leads to its coherent projection over length scales up to 20 times larger than its localization length, thereby satisfying the fundamental requirement for the non-local detection of quantum states. Our interpretation of Fig. 4c,e as showing a projection of the native YSR state is further supported by the observation that, when the Fe atom is replaced by a non-magnetic Ag atom, no in-gap state exists at ±*E*_*β*_ (Fig. 4b,d,f). Finally, the YSR quantum projection in this particular rectangular corral, in contrast to the elliptical one, possesses the same qualitative p–h composition as the native Fe YSR state (Figs. 4e, 3j and 2b). This raises the question of whether this composition, and not only the spatial structure of the projection, can be manipulated by shifting the energies of the corral eigenmodes through *E*_F_.

## Manipulating the spatial form and p–h composition of projected YSR states

**Fig. 5:**
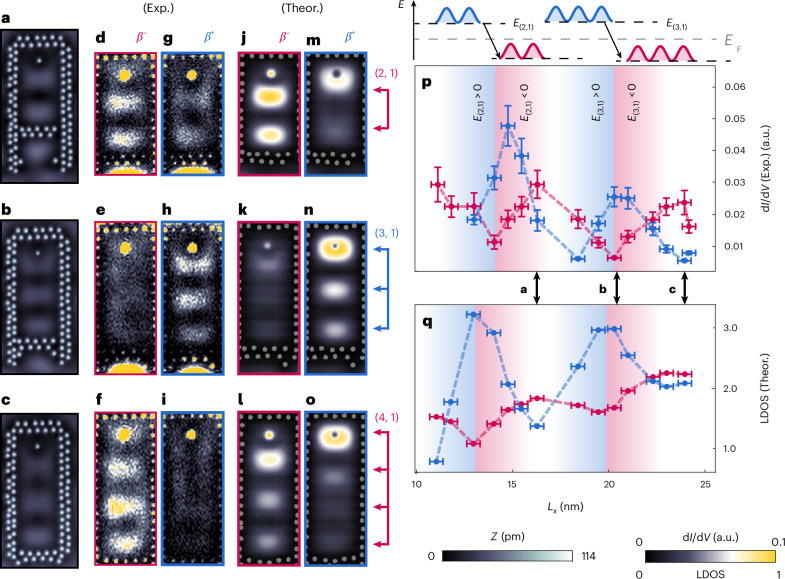
Oscillation of p–h composition of the YSR quantum projection. **a**–**c**, Constant-current STM images of Ag corrals, including an Fe atom at the top, with constant widths *L*_*y*_ = 9.1 nm and different *L*_*x*_ values of 16.29 nm (**a**), 20.27 nm (**b**) and 23.91 nm (**c**) (*V*_bias_ = −5 mV, *I*_set_ = 1 nA). **d**–**o**, Experimental (Exp.) constant-height d*I*/d*V* maps (**d**–**i**) and theoretical (Theor.) LDOS maps (**j**–**o**) taken inside the corrals of **a**–**c** close to −*E*_*β*_ (**d**–**f**: *V*_bias_ = −1.67 mV; **j**–**l**: for the theoretical parameters, see Supplementary Note 3) and +*E*_*β*_ (**g**–**i**: *V*_bias_ = 1.67 mV; **m**–**o**: for the theoretical parameters, see Supplementary Note 3) (*V*_stab_ = −5 mV, *I*_stab_ = 1 nA, ${V}_{{\rm{mod}}}=100\,\upmu {\rm{V}}$). **p**,**q**, Experimental (**p**) and theoretical (**q**) intensities of the *β*^−^ (red) and *β*^+^ (blue) components of the YSR quantum projection as a function of corral lengths *L*_*x*_ extracted from corrals with *L*_*x*_ values ranging from 4.7 nm to 24.1 nm, as described in the main text (the horizontal errors are defined by the deviation in the short corral side’s inner-wall atoms from a straight line; the vertical experimental errors are described in Supplementary Note 7). The sketch on the top of **p** and the corresponding blue- and red-shaded areas indicate the lengths at which the energies ${E}_{({n}_{x},{n}_{y})}$ of the eigenmode with the given quantum number (*n*_*x*_, *n*_*y*_) cross *E*_F_ (Supplementary Notes 3 and 4 and Supplementary Figs. 10 and 16). The double arrows underneath **p** indicate the lengths of the corrals shown in **a**–**c**. a.u.: output voltage (in volts) of the lock-in amplifier using the same lock-in parameters in all the d*I*/d*V* maps (**d**–**i**) and **p**.

To investigate this question, we consider a series of quantum corrals with increasing length *L*_*x*_, allowing us to shift the energies of several eigenmodes through the SC gap. Figure 5a–c shows the constant-current STM images for three different corral lengths, with the corral eigenmodes being located at different distances from *E*_F_. The corresponding d*I*/d*V* maps taken inside the corrals close to −*E*_*β*_ and +*E*_*β*_ (Fig. 5d–f and Fig. 5g–i, respectively) reveal again the existence of a YSR quantum projection; they are well reproduced by the corresponding theoretically computed LDOS (Fig. 5j–l and Fig. 5m–o, respectively). Although for the corrals shown in Fig. 5a,c, with the eigenmodes located well outside the gap (Supplementary Notes 3 and 4), the intensity of the *β*^−^ component is larger than that of the *β*^+^ component, this p–h composition is reversed for the corral shown in Fig. 5b, where the (3, 1) corral eigenmode in the absence of the SC would be located very close to *E*_F_. The spatially resolved p–h asymmetry within these three corrals is shown in Supplementary Fig. 20 and described in Supplementary Note 6. A plot of the *β*^−^ and *β*^+^ quantum projection intensities for a larger number of quantum corral lengths *L*_*x*_ (Supplementary Fig. 19 and Supplementary Note 7) reveals an oscillatory behaviour of the intensities, with the *β*^+^ projection intensity always being larger than the *β*^−^ projection intensity whenever a corral eigenmode is very close to *E*_F_ (Fig. 5p; the blue and red-shaded areas show the ranges in which the respective corral eigenmode is above and below *E*_F_, respectively). At the same time, the energy of the YSR projection remains essentially unchanged and close to ±*E*_*β*_ (Supplementary Figs. 23a and 24). These features are well reproduced by the theoretically computed intensities shown in Fig. 5q (Supplementary Note 3). Thus, by tuning the energy of a corral eigenmode close to *E*_F_, it is possible to manipulate the p–h composition of the YSR projection, as reflected in the intensities of the *β*^+^ and *β*^−^ components. Moreover, this manipulation of the p–h composition can occur while the projections’ spatial shape remains essentially unaltered, as follows from a comparison of the d*I*/d*V* maps for corrals of length *L*_*x*_ = 18.4 nm and *L*_*x*_ = 20.3 nm (Supplementary Fig. 19).

The dependence of the intensities of the *β*^±^ components in the YSR projection on the corral length is mainly determined by the p–h asymmetry of the corral eigenmodes. Indeed, the calculation of the LDOS inside the corral (Supplementary Equation (38)) includes both real and imaginary parts of the corral eigenmodes (Supplementary Note 3), both of which are large and highly p–h asymmetric when the eigenmode is near *E*_F_, but vanishingly small otherwise (Supplementary Fig. 8). Although this is reminiscent of the well-known dependence of the p–h asymmetry of a YSR state arising from a single magnetic impurity on the p–h asymmetry of the conduction band in the normal state^24^, in our case, the p–h asymmetry of the YSR state projection arises from the p–h asymmetry of the projecting subsystem, that is, the corral eigenmodes. To what extent the eigenmodes’ p–h asymmetry is imprinted onto that of the YSR projection, however, is not universal, and strongly depends on the hybridization *V*_hyb_ between the Shockley surface states and the Ag island bulk bands (Supplementary Fig. 12 and Supplementary Note 3). Indeed, by reducing the complexity of the theoretical model to a model in which a single orbital of an impurity is coupled to a simplified zero-dimensional but still highly p–h-asymmetric MSS via the Ag bulk states, we reproduce not only the qualitative behaviour shown in Fig. 5p,q (Supplementary Fig. 25 and Supplementary Note 9) but also the dependence on *V*_hyb_ (Supplementary Fig. 26).

## Discussion and outlook

Our theoretical models predict an abrupt change in the relative intensities of the p–h components of the YSR projection when the state’s energy crosses *E*_F_ (Supplementary Figs. 13 and 25), for example, by tuning the coupling of the magnetic adatom to the substrate^33,34^. Hence, energy crossings of the YSR states, which are known to be accompanied by a change in the spin ground state of the SC^24,33,34^, could also be measured indirectly via the YSR projection—a prediction that could be tested in future work and help to identify the quantum phase transitions of single-atom, multiorbital YSR states on SC surfaces^35^.

We foresee intriguing possibilities if the Fe was replaced by magnetic atoms that couple more strongly to the Shockley surface state^36,37^, thereby realizing more strongly coupled YSR quantum corral hybrid states. In such systems, the feasibility of engineering the strength and landscape of couplings of distant atomic YSR states was theoretically predicted^17,20,38–43^. The range and potential of the above-demonstrated methodology presents an approach to manipulate the extent of the (local) p–h mixing in the SC state, and to non-locally access and manipulate YSR qubits^44^ or elusive quantum states like p–h-symmetric Majorana bound states^45–47^ over length scales that are orders of magnitudes larger than their typical localization lengths in a minimal-invasive fashion.

## Methods

### Experimental methods

The experimental work was performed in a SPECS STM system operated at *T* = 4.5 K, which is equipped with custom-built ultrahigh-vacuum chambers for sample preparation^48^. Constant-current STM images were recorded with a closed feedback loop at a tunnel current *I*_set_ and with a bias voltage *V*_bias_ applied to the sample. For measurements of the differential tunnel conductance (d*I*/d*V*), which is closely related to the sample’s LDOS below the tip, we used four different modes, applying standard lock-in techniques with a small modulation voltage ${V}_{{\rm{mod}}}$ (r.m.s.) of frequency *f* = 1,097.1 Hz added to *V*_bias_. (1) A single d*I*/d*V* spectrum was recorded after stabilizing the tip on the desired location at *I*_stab_ and *V*_stab_, switching the feedback off and recording d*I*/d*V* as a function of *V*_bias_. (2) The so-called d*I*/d*V* line profiles are the d*I*/d*V* spectra recorded laterally at many locations along a line using the same measurement mode at parameters *I*_stab_ and *V*_stab_. (3) For the so-called constant-contour d*I*/d*V* maps, we first recorded a *z* contour using a constant-current STM image at *I*_stab_ and *V*_stab_ and then switched the feedback off and measured d*I*/d*V* at *V*_bias_ using the same contour. (4) For the so-called constant-height d*I*/d*V* maps, we stabilized the vertical tip position *z* on a Ag atom of the corral wall at *I*_stab_ and *V*_stab_ and then switched the feedback off and recorded the d*I*/d*V* spatially resolved at *V*_bias_ keeping the same *z*.

The STM tips were prepared from a mechanically cut and sharpened high-purity Nb wire and flashed in situ to about 1,500 K to remove residual contaminants or oxide layers. STS measurements with superconducting tips have an increased effective energy resolution but require careful interpretation of the acquired d*I*/d*V* data. As, for example, in ref. ^49^, due to the convolution between the SC tip’s density of states and the SC sample’s LDOS, all the spectroscopic features above and below *E*_F_ are shifted by approximately the tip gap ±*Δ*_t_ = ±1.32 meV. Furthermore, at finite temperatures, thermal replicas appear at bias values between −*Δ*_t_ and +*Δ*_t_, leading to redundant spectral information in the measurement. Due to these effects, it is sufficient to look at the spectral features below −*Δ*_t_ and above +*Δ*_t_. For the sake of clarity, in the spectroscopy data, we cut out the portions between −*Δ*_t_ and +*Δ*_t_. The full spectra are provided in the Supplementary Information.

We used a Nb(110) crystal, which was first flashed under ultrahigh-vacuum conditions to *T* ≈ 2,000 K. This treatment left us with a Nb(110) surface, which is still covered by an oxide layer^29^. We then deposited seven monolayers of Ag onto this surface^29^. Thereafter, Fe was deposited onto the cold sample kept at a temperature *T* < 10 K from the Fe rod of a thoroughly outgassed electron-beam evaporator, resulting in a statistical distribution of single Fe atoms on the surface. Using the STM tip, Ag atoms were extracted from the Ag substrate, as described previously^29^. Subsequently, after gathering a sufficient number of Ag atoms, the Ag quantum corrals, including the Fe atoms inside, were constructed by lateral atom manipulation at low tunnelling resistances of *G* ≈ 100 kΩ. Because the Ag walls of the corrals have a finite transparency for the surface-state electrons, we constructed a second wall of Ag atoms around the central wall^29^.

### Tight-binding Hamiltonian

We consider a semi-infinite 3D tight-binding Hamiltonian *H* = *H*_3D_ + *H*_s_ + *H*_at_ + *H*_c_ + *H*_hyb_ (Supplementary Note 3), where *H*_3D_ describes the semi-infinite 3D electronic structure of the Ag island, *H*_s_ describes the electronic Shockley surface-state band, *H*_hyb_ represents the coupling between the surface and bulk states (which leads to proximity-induced superconductivity in the former), *H*_c_ describes the coupling of a corral of non-magnetic Ag atoms to the surface states, giving rise to the eigenmode structure of the surface band inside the corral, and *H*_at_ represents the scattering arising from the presence of a Ag or Fe atom inside the corral, where we assume that the Fe (Ag) atoms couple to the bulk (surface) states only.

## Online content

Any methods, additional references, Nature Portfolio reporting summaries, source data, extended data, supplementary information, acknowledgements, peer review information; details of author contributions and competing interests; and statements of data and code availability are available at 10.1038/s41567-025-03109-y.

## Supplementary information

Supplementary Information Supplementary Notes 1–9, Figs. 1–26, Equations (1)–(44) and references.

## Data Availability

The raw data used to generate the plots in the paper are available via Zenodo at 10.5281/zenodo.17341988 (ref. ^50^). The other datasets produced or examined in this study can be obtained from the corresponding author upon reasonable request.

## Code availability

The codes generated during the current study are available from the corresponding author on reasonable request.
