# Supplementary material for: Non-local detection of coherent Yu–Shiba–Rusinov quantum projections
Source: Nat Phys. 2025 Nov 26;22(1):54–60. doi: 10.1038/s41567-025-03109-y (PMC12811141; doi:10.1038/s41567-025-03109-y)
Supplement: Supplementary file 1 — Supplementary Notes 1–9, Figs. 1–26, Equations (1)–(44) and references. [file 41567_2025_3109_MOESM1_ESM.pdf]

nature physics

Supplementary information https://doi.org/10.1038/s41567-025-03109-y

Non-local detection of coherent Yu–Shiba–Rusinov quantum projections

In the format provided by the authors and unedited

Contents

Supplementary Note 1 — Superconducting tip gap 2

Supplementary Note 2 — Native YSR states of the Fe atom 4

Supplementary Note 3 — Semi-infinite 3D tight-binding model 7

Supplementary Note 4 — Determination of the corral length dependent eigenmode energies 25

Supplementary Note 5 — $\mathrm{d}I/\mathrm{d}V$ spectra in rectangular corrals with Ag and Fe atoms 31

Supplementary Note 6 — Spatially resolved p-h asymmetry of the YSR quantum projection 34

Supplementary Note 7 — Fitting procedures for the β YSR quantum projection 36

Supplementary Note 8 — $\beta^{\pm}$ YSR states on Fe atoms in the corral 40

Supplementary Note 9 — Effective model and analytical results 42

Supplementary References 47

Supplementary Note 1 Superconducting tip gap

Δ_t was determined as described in the following. We simulated the tunneling current between the tip and the sample, and then took the numerical derivative of the current (see Fig. S1). We used the following expression to simulate the tunneling current between the tip and the sample with the bias-voltage V applied between them:

$$I \propto \int_{-\infty}^{+\infty} \rho_t(\epsilon - eV)\rho_s(\epsilon)\left(f(\epsilon - eV) - f(\epsilon)\right) d\epsilon. \quad (1)$$

Here f is the Fermi-function given by

$$f(\epsilon, T) = \frac{1}{e^{\epsilon/k_B T} + 1}. \quad (2)$$

with $\epsilon = E - E_F$. The LDOS of the superconducting Nb tip ρ_t is simulated by a Dynes function

$$\rho_t = N_{0,t}\Re\left[\frac{\epsilon - i\gamma_t}{\sqrt{(\epsilon - i\gamma_t)^2 - \Delta_t^2}}\right], \quad (3)$$

where $N_{0,t}$ is the normal state LDOS, γ_t is the lifetime broadening parameter accounting for a finite lifetime of the quasiparticle excitations outside the gap, and $\Re$ denotes the real part. Since we operate at a small bias voltage range, we assumed that $N_{0,t}$ is constant. The coherence peaks of the LDOS of the sample ρ_s were also simulated by a Dynes function, now with the parameters $N_{0,s}$, Δ_s and γ_s. The in-gap features, i.e., the negative and positive energy MSS (index $j = 1, 2$) and the negative (β^-) and positive energy (β^+) YSR states ($j = 3, 4$), were modeled by Lorentzian peaks:

$$L(\epsilon, I_j, \epsilon_j, \gamma_j) = \frac{I_j\gamma_j}{\gamma_j^2 + (\epsilon - \epsilon_j)^2} \quad (4)$$

with I_j being the height, ϵ_j the energetic location, and γ_j the width of the peak. Using Eq. (3) and Eq. (4) we modelled the sample's LDOS as:

$$\rho_s = N_{0,s}\Re\left[\frac{\epsilon - i\gamma_s}{\sqrt{(\epsilon - i\gamma_s)^2 - \Delta_s^2}}\right] + \sum_{j=1}^{4} L(\epsilon, I_j, \epsilon_j, \gamma_j) \quad (5)$$

with the summation over the positive and negative energy MSS and β YSR states. Since the broadening parameters γ_j only change the width of the peaks, but not their energetic location, and we merely want to determine the tip gap Δ_t, we fix $\gamma_j = 0.05\,\text{meV}$ ($j = 1, 2, 3, 4$) for the sake of simplicity. The remaining parameters are adjusted to fit the spectra in Fig. S1 measured on the substrate (panel A) and on a Fe atom (panel B). The fit parameters are given in the figure caption. We determined a value of $\Delta_t = 1.32\,\text{meV}$.

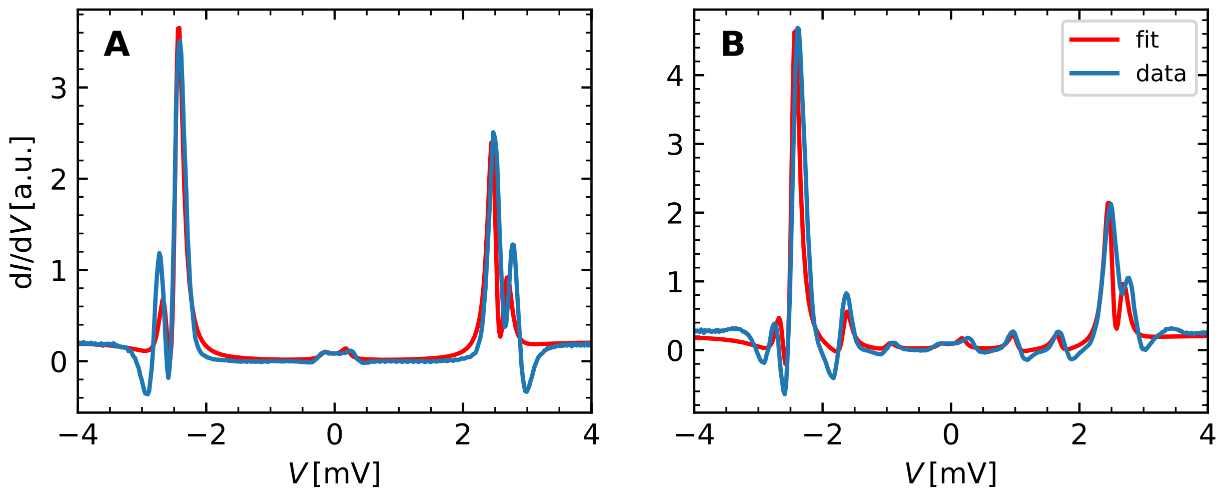

Supplementary Figure 1 | Determination of the tip gap. $\mathrm{d}I/\mathrm{d}V$ spectra (blue) and fits (red) taken on a substrate location (**A**) and on a Fe atom (**B**) ($V_{\mathrm{stab}} = -5\,\mathrm{mV}$, $I_{\mathrm{stab}} = 1\,\mathrm{nA}$, $V_{\mathrm{mod}} = 50\,\mu\mathrm{V}$). Fitting parameters: $T = 4.54\,\mathrm{K}$, $\Delta_{\mathrm{s}} = 1.35\,\mathrm{meV}$, $\Delta_{\mathrm{t}} = 1.32\,\mathrm{meV}$, $\epsilon_{1,2} = \pm 1.15\,\mathrm{meV}$ (A,B), $\epsilon_{3,4} = \pm 0.34\,\mathrm{meV}$ (**B**).

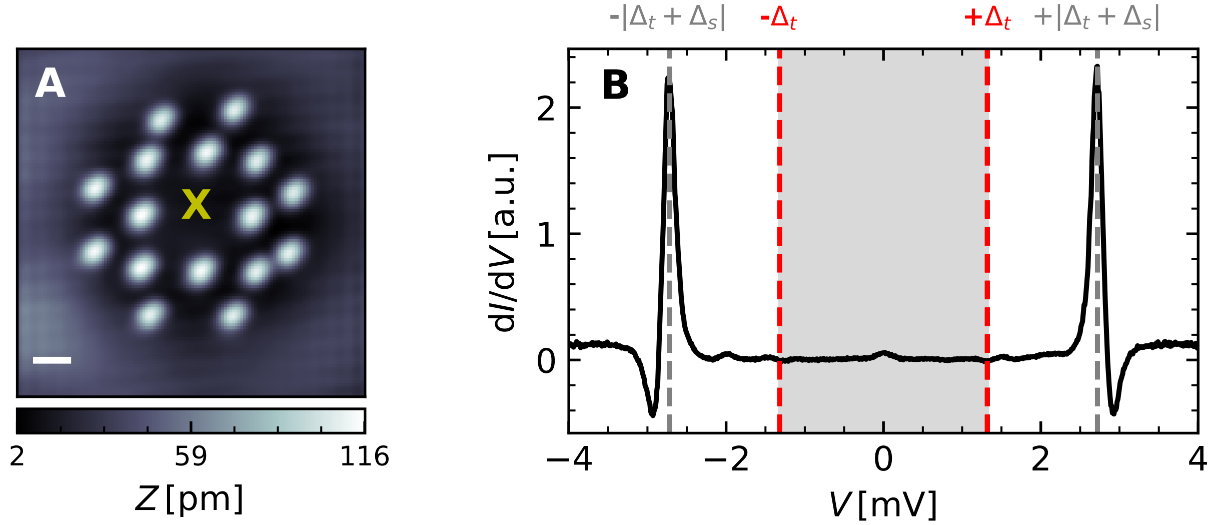

Supplementary Figure 2 | De Gennes-Saint James coherence peaks of the proximitized Ag island. (**A**) Constant-current STM image of a Ag corral of very small size (scale bar corresponds to $1\,\mathrm{nm}$, $V_{\mathrm{bias}} = 15\,\mathrm{mV}$, $I_{\mathrm{set}} = 1\,\mathrm{nA}$). (**B**) $\mathrm{d}I/\mathrm{d}V$ spectrum taken inside the Ag corral at the location of the cross shown in (A) measured with the superconducting Nb tip. The dashed lines mark the biases corresponding to the tip gap $\pm\frac{\Delta_{\mathrm{t}}}{e} = \pm 1.32\,\mathrm{mV}$ and to the De Gennes-Saint James coherence peaks $\pm\frac{|\Delta_{\mathrm{t}}+\Delta_{\mathrm{s}}|}{e} = \pm 2.67\,\mathrm{mV}$. This measurement is also shown in Fig. 2b of the main text ($V_{\mathrm{stab}} = 5\,\mathrm{mV}$, $I_{\mathrm{stab}} = 1\,\mathrm{nA}$, $V_{\mathrm{mod}} = 20\,\mu\mathrm{V}$).

Supplementary Note 2 Native YSR states of the Fe atom

The spectra taken on the Fe atom (Figs. S3A,B and red curve in Fig. 2b of the main text) reveal three pairs of peaks or shoulders in between the substrate coherence peaks at $-1.61\,\mathrm{meV}$ (β^-) and $+1.61\,\mathrm{meV}$ (β^+), at $-2.33\,\mathrm{meV}$ (γ^-) and $2.33\,\mathrm{meV}$ (γ^+), and at $-2.41\,\mathrm{meV}$ (α^-) and $2.41\,\mathrm{meV}$ (α^+). They are due to the particle-hole partners of three YSR states resulting from the coupling of the Fe $3d$ orbitals to the proximitized Ag film. The constant-contour $\mathrm{d}I/\mathrm{d}V$ maps taken at the biases of the above in-gap peaks or shoulders of the Fe atom, which are closely related to the LDOS of its respective YSR states, are shown in Fig. S3C to F. While the most intense α^- YSR state (Fig. S3E) has a circularly symmetric shape with maximum intensity above the atom, the β^- and β^+ YSR states have intermediate intensities and resemble downwards and upwards pointing triangles, respectively, with intensity minima above the atom (Figs. S3C,D). Finally, the weakest intensity γ^+ YSR state (Fig. S3F) has a spatially more extended triangular shape, again with a central intensity minimum. This threefold multiplicity of the YSR states of the $3d$ transition metal atom is consistent with its three-fold coordinated hollow adsorption site on the Ag(111) surface, which is subject to a trigonal pyramidal crystal field [1]. Considering the different intensities, symmetries and the energetical order of the three YSR states, their most likely assignment to the Fe $3d$ orbitals is the following. The α YSR state stems from the d_z^2 orbital, the γ from the (almost) degenerate xy and $x^2 - y^2$ orbitals, and the β from the almost degenerate xz and yz orbitals [1]. Most importantly, all states except for the γ YSR state have a similar spatial extent ($\varnothing \approx 1.5\,\mathrm{nm}$) as the apparent diameter of the Fe atom which is extracted from the STM images (see Figs. S3G to J). Note, that spectra taken on the Ag atoms (see Figs. 3j and 4f of the main text, as well as Figs. S18E,F) are essentially indistinguishable from the substrate and do not show any YSR states, indicating their nonmagnetic character.

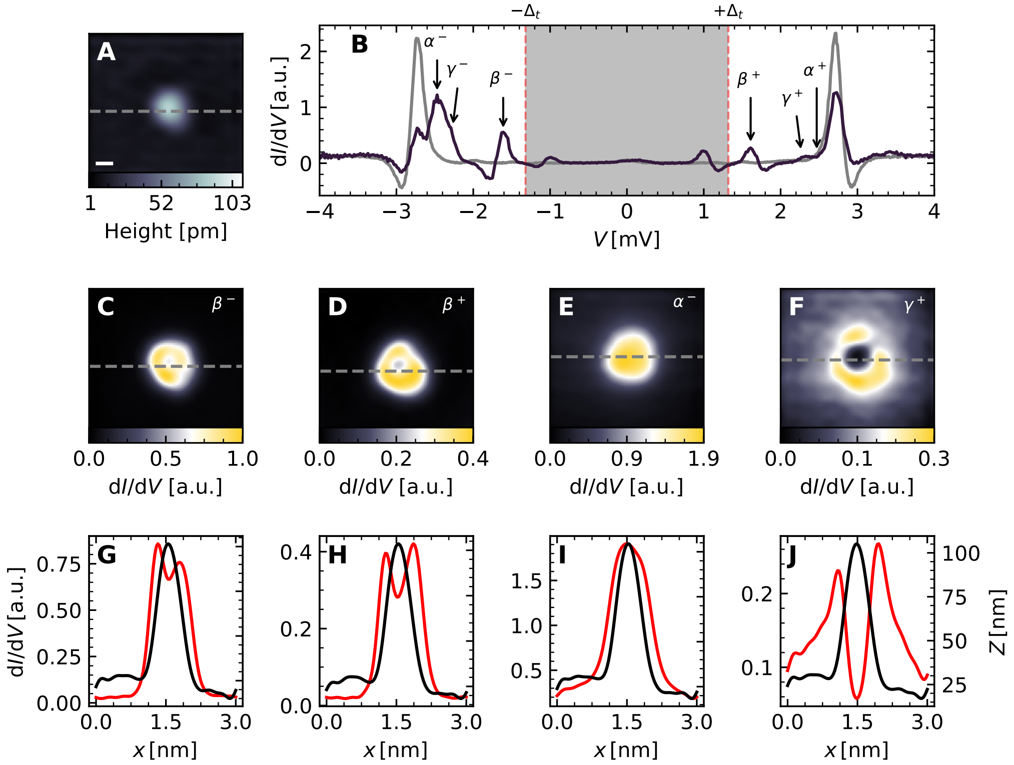

Supplementary Figure 3 | Native YSR states of the Fe atom. (**A**) Constant-current STM image of the same single Fe atom in the center of the same rectangular Ag corral shown in Fig. 2 of the main text (scale bar: $300\,\mathrm{pm}$, $V_{\mathrm{bias}} = 5\,\mathrm{mV}$, $I_{\mathrm{set}} = 1\,\mathrm{nA}$). (**B**) $\mathrm{d}I/\mathrm{d}V$ spectra taken on the Fe atom (black curve) shown in (A) and on the substrate (gray curve) from Fig. S2. This measurement is also shown in Fig. 2b of the main text. The three particle-hole pairs of YSR states are indicated by arrows ($V_{\mathrm{stab}} = 5\,\mathrm{mV}$, $I_{\mathrm{stab}} = 1\,\mathrm{nA}$, $V_{\mathrm{mod}} = 50\,\mu\mathrm{V}$ for the Fe atom, $V_{\mathrm{mod}} = 20\,\mu\mathrm{V}$ for the substrate). (**C** to **F**) Constant-contour $\mathrm{d}I/\mathrm{d}V$ maps of the Fe atom acquired at the respective bias voltages $V_{\mathrm{bias}} = -1.61\,\mathrm{mV}$ of the β^- state (C, same as in Fig. 2c of the main text), $V_{\mathrm{bias}} = 1.61\,\mathrm{mV}$ of the β^+ state (D, same as in Fig. 2d of the main text), $V_{\mathrm{bias}} = -2.41\,\mathrm{mV}$ of the α^- state (E), and $V_{\mathrm{bias}} = 2.33\,\mathrm{mV}$ of the γ^+ state (F). Measurement parameters: $V_{\mathrm{stab}} = 5\,\mathrm{mV}$, $I_{\mathrm{stab}} = 1\,\mathrm{nA}$, $V_{\mathrm{mod}} = 100\,\mu\mathrm{V}$. (**G** to **J**) Black and red lines are line profiles taken from the STM image in (A) and from the corresponding constant-contour $\mathrm{d}I/\mathrm{d}V$ maps in (C to F), respectively, along the gray dashed lines in (A) and (C to F).

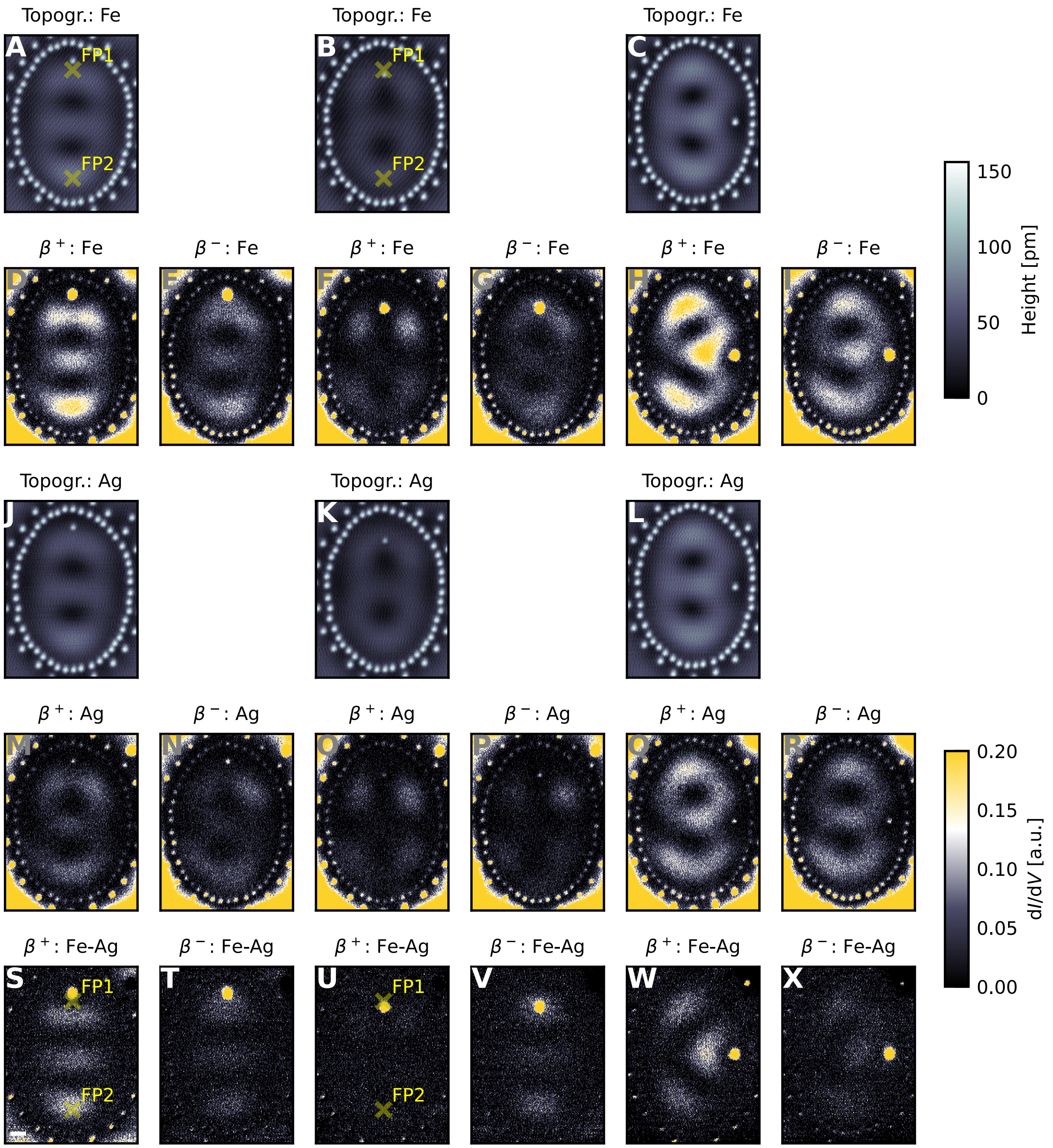

Supplementary Figure 4 | YSR quantum projection in an elliptical corral for different Fe locations. (**A** to **C**) Constant-current STM images of the same elliptical corral as in Fig. 3 of the main text with the Fe atom placed slightly above (A), slightly below (B) and completely off (C) the top focal point FP1 ($V_{\mathrm{bias}} = -5\,\mathrm{mV}$, $I_{\mathrm{set}} = 1\,\mathrm{nA}$). (**D** to **I**) Constant-height $\mathrm{d}I/\mathrm{d}V$ maps taken, as indicated, close to the bias corresponding to $\pm E_\beta$ for the corrals in (A) to (C), respectively (D, F, H: $V_{\mathrm{bias}} = 1.67\,\mathrm{mV}$; E, G, I: $V_{\mathrm{bias}} = -1.67\,\mathrm{mV}$, $V_{\mathrm{stab}} = -5\,\mathrm{mV}$, $I_{\mathrm{stab}} = 1\,\mathrm{nA}$, $V_{\mathrm{mod}} = 100\,\mu\mathrm{V}$). (**J** to **R**) Same as (A) to (I), but with the Fe atom replaced by a Ag atom. (**S** to **X**) Subtraction maps of the constant-height $\mathrm{d}I/\mathrm{d}V$ maps of the corrals with the Fe atom and with the Ag atom (S = D - M, T = E - N, etc.). Scale bar in (S) corresponds to $1\,\mathrm{nm}$.

Supplementary Note 3 Semi-infinite 3D tight-binding model

The semi-infinite three-dimensional (3D) tight-binding model consists of a two-dimensional surface Ag layer that is coupled to a 3D bulk band, as schematically shown in Fig. S5. Moreover, the quantum corral consisting of Ag adatoms is described by a nonmagnetic scattering potential that couples to the Shockley surface-states, while the Fe adatom is described by a magnetic scattering potential that couples to the electronic states in the top-most bulk layer. In the following, we provide a detailed discussion of the Hamiltonian describing the various components of this model, and how we computed the LDOS presented in the main text.

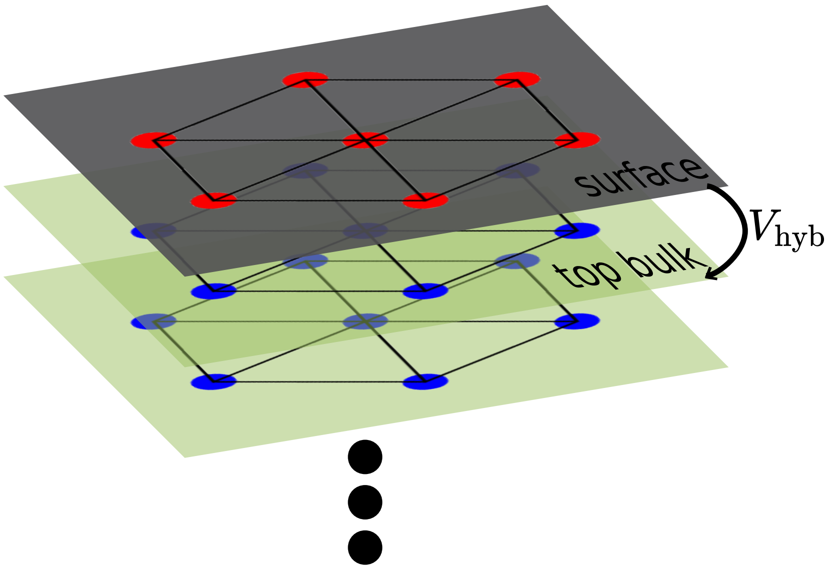

Supplementary Figure 5 | Schematic representation of the semi-infinite 3D tight-binding model. The gray layer represents the Shockley surface-states, while the green layers represent the bulk layers of the Ag island. V_{hyb} represents the hybridization between the Shockley surface-states (red dots) and the electronic states of the top bulk layer (blue dots).

Electronic structure of the surface state and eigenmodes of the quantum corral The electronic structure of the 2D surface band comprised of Shockley surface-states is described by the Hamiltonian:

$$H_{\text{s}} = -t \sum_{\langle \mathbf{r}\mathbf{r}' \rangle, \sigma} f^{\dagger}_{\mathbf{r},\sigma} f_{\mathbf{r}',\sigma} - \mu \sum_{\mathbf{r},\sigma} f^{\dagger}_{\mathbf{r},\sigma} f_{\mathbf{r},\sigma} + H.c. \tag{6}$$

where $f^\dagger_{\mathbf{r},\sigma}$ creates an electron with spin σ at site $\mathbf{r}$, $-t$ is the electronic hopping between the nearest-neighbor sites on a 2D hexagonal lattice, and μ is the chemical potential. The dispersion of the surface band in Ag(111) was previously determined using quasi-particle interference (QPI) experiments; we adjusted the relevant parameters slightly to reproduce the energy position of the observed eigenmodes, yielding $t = 850$ meV and $\mu = -5071.4$ meV [2,3].

The quantum corral consists of Ag adatoms; their effect on the surface band is described by a nonmagnetic scattering potential and the Hamiltonian

$$H_{\mathrm{c}} = -U \sum_{\mathbf{R},\sigma} f^\dagger_{\mathbf{R},\sigma} f_{\mathbf{R},\sigma} \ , \tag{7}$$

where $f^\dagger_{\mathbf{R},\sigma}$ creates a fermion at site $\mathbf{R}$ and spin σ on the surface, and the sum runs over all sites $\mathbf{R}$ of the Ag adatoms that form the corral. The real space positions of the Ag adatoms are obtained from the experimental constant-current STM images shown in Fig. S6A and are shown in Fig. S6B. Since the bright spots representing the Ag adatoms in the topography plot of Fig. S6A are spatially extended over a few lattice sites, we assign the nonmagnetic scattering potential U to the center site of each bright spot, as well as its six nearest-neighbor sites. Moreover, we take $U = 2000$ meV to reproduce the experimentally measured energies [2] of the corral eigenmodes.

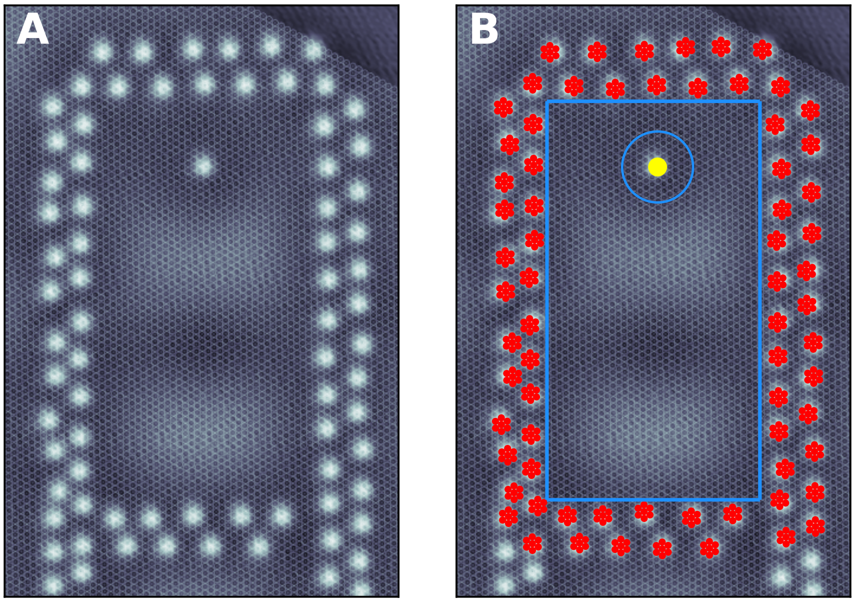

Supplementary Figure 6 | Determination of the real space locations of the corral Ag adatoms. (**A**) Experimental constant-current STM image of the rectangular quantum corral with width $L_y = 9.1$ nm and length $L_x = 16.29$ nm. (**B**) Theoretical model for the locations of the Ag adatoms using the adatom positions from (A). The red dots represent the center sites of each Ag adatom that forms the corral, as well as their six nearest-neighboring sites. The yellow dot represents the site of the single Ag or Fe adatom inside the corral. The region between the blue lines is used to compute the integrated intensity of the $\beta^{\pm}$-branches shown in Fig. 5q of the main text. A region with radius of approximately 5 lattice sites around the Fe impurity (enclosed by the circular blue line) is excluded from the integration.

We begin by considering the spatial and energetic form of the eigenmodes of the quantum corral. To this end, we write the total Hamiltonian for the surface band and quantum corral $H_{\rm sc} = H_{\rm s} + H_{\rm c}$ in matrix form via

$$H_{\rm sc} = \Psi^\dagger \hat{H}_{\rm sc} \Psi \ , \tag{8}$$

where we defined the spinor in Nambu space,

$$\Psi^\dagger = (f^\dagger_{1,\uparrow}, f_{1,\downarrow}, f^\dagger_{2,\uparrow}, f_{2,\downarrow}, ..., f^\dagger_{i,\uparrow}, f_{i,\downarrow}, ..., f^\dagger_{N,\uparrow}, f_{N,\downarrow}) \tag{9}$$

with $i = 1, 2, ..., N$ being the site indices of the system with N sites. The energies of the corral eigenmodes are obtained by diagonalizing $\hat{H}_{\rm sc}$. To obtain the form of the LDOS of of these eigenmodes, we compute the retarded Greens function using

$$\hat{G}_{\rm sc}(\omega) = [(\omega + i\Gamma)\hat{I} - \hat{H}_{\rm sc}]^{-1} \tag{10}$$

with $\tau = \hbar/\Gamma$ being the lifetime of the eigenmodes, and $\hat{I}$ being the identity marix. The LDOS $N(E, \mathbf{r})$ as a function of energy E and position $\mathbf{r}$ is then given by

$$N(E, \mathbf{r}) = -\frac{1}{\pi} \mathrm{Im} \left[\hat{\mathrm{G}}_{\mathrm{sc}}(\omega = \mathrm{E}/\hbar) \right]_{\mathrm{ii}} \ , \tag{11}$$

where $\left[\hat{G}_{\mathrm{sc}}(\omega) \right]_{ii}$ is the (ii) element (with i being the site index) of the matrix $\hat{G}_{\mathrm{s}}$ corresponding to position $\mathbf{r}$.

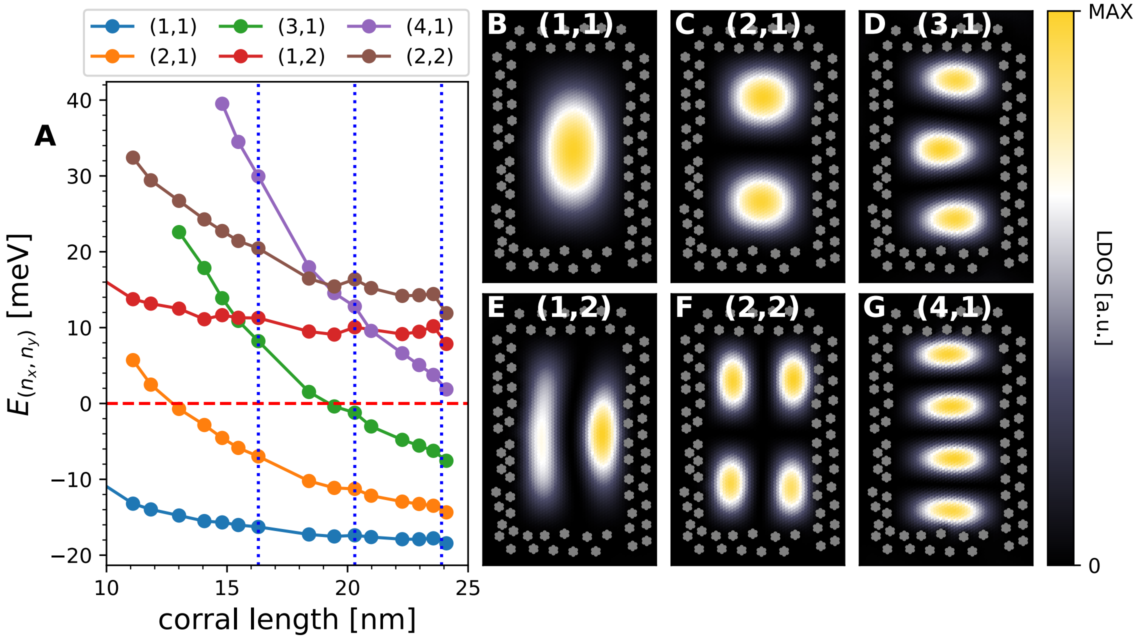

Supplementary Figure 7 | Energies of the corral eigenmodes. (**A**) Energies $E_{(n_x,n_y)}$ of the corral eigenmode (n_x, n_y) as a function of the corral length. The dashed vertical lines indicate the lengths of the corrals shown in Fig. 5 of the main text. (**B** to **G**) Spatial LDOS plots of the six lowest energy eigenmodes for a corral of length $L_x = 16.3$ nm.

The dependence of the eigenmode energies on the corral lengths for the six lowest energy eigenmodes is shown in Fig. S7A; the corresponding spatial LDOS plots of these six eigenmodes for a corral of length $L_x = 16.3$ nm are shown in Figs. S7B to G. These eigenmodes are characterized by the number of maxima in the LDOS in x- and y-directions, given by n_x and n_y, respectively.

Bulk states of the Ag island We next consider the bulk states of the Ag island. Since no first-principle calculations for the electronic structure of such an island are currently available, we

model the Ag island as a system of M Ag layers with a hexagonal lattice that are coupled via an interlayer hopping. Each of the individual layers is described by the Hamiltonian $(l = 1, ..., M)$,

$$H_l = -t_{\rm b} \sum_{\langle \mathbf{r}\mathbf{r}' \rangle, \sigma} d^\dagger_{l,\mathbf{r},\sigma} d_{l,\mathbf{r}',\sigma} - \mu_{\rm b} \sum_{l,\mathbf{r},\sigma} d^\dagger_{l,\mathbf{r},\sigma} d_{l,\mathbf{r},\sigma} + \Delta_0 \sum_{\mathbf{r}} d^\dagger_{l,\mathbf{r},\uparrow} d^\dagger_{l,\mathbf{r},\downarrow} + H.c. \tag{12}$$

where $-t_{\rm b}$ is the intralayer electronic hopping amplitude between nearest neighbor sites on a hexagonal lattice (we assume the same lattice structure as for the surface band), $d^\dagger_{l,\mathbf{r},\sigma}(d_{l,\mathbf{r},\sigma})$ creates (destroys) an electron with spin σ at site $\mathbf{r}$ in layer l, $\mu_{\rm b}$ is the bulk chemical potential, and Δ_0 is the superconducting order parameter induced in the Ag island. The values of the relevant band parameters are discussed below.

Using the spinor

$$\Phi^\dagger_l = (d^\dagger_{l,1,\uparrow}, d_{l,1,\downarrow}, d^\dagger_{l,2,\uparrow}, d_{l,2,\downarrow}, ..., d^\dagger_{l,i,\uparrow}, d_{l,i,\downarrow}, , ...d^\dagger_{l,N,\uparrow}, d_{l,N,\downarrow}) \ , \tag{13}$$

where l is the layer index, and i is the site index on layer l, we obtain from Eq. (12) the matrix form of the Hamiltonian,

$$H_l = \Phi^\dagger_l \hat{H}_l \Phi_l \ , \tag{14}$$

where $\hat{H}_l = \hat{H}$ is the same for all layers. Moreover, the bulk layers l and $l+1$ are coupled via an interlayer hopping described by

$$T_{l,l+1} = -t_\perp \sum_{\mathbf{r},\sigma} d^\dagger_{l,\mathbf{r},\sigma} d_{l+1,\mathbf{r},\sigma} = \Phi^\dagger_l \hat{T}_{l,l+1} \Phi_{l+1} \ , \tag{15}$$

where $\hat{T}_{l,l+1} = \hat{T}$ is the matrix form of the above Hamiltonian, which is the same for all layers, and $-t_\perp$ is the interlayer electronic hopping amlitude between nearest neighbor sites from neighboring layers. For simplicity, we assume a stacking in which the bulk sites along the z-axis are stacked on top of each other. The Hamiltonian matrix for the entire Ag bulk island is then given by

$$\hat{H}_{\rm 3D} \quad = \quad \begin{pmatrix} \hat{H}_1 & \hat{T}_{1,2} & & \\ \hat{T}^\dagger{}_{2,1} & \hat{H}_2 & \hat{T}_{2,3} & \\ & \hat{T}^\dagger{}_{3,2} & \ddots & \ddots \\ & & \ddots & \\ & & & \end{pmatrix} \tag{16}$$

The retarded Greens functions of the bulk Ag system in matrix form, are then given by

$$\hat{G}_{\rm 3D}(\omega) = [(\omega + i\Gamma)\hat{I} - \hat{H}_{\rm 3D}]^{-1} \tag{17}$$

where $\hat{I}$ is the identity matrix. Next, by defining intra- $(l = m)$ and inter-layer $(l \neq m)$ Greens function matrices in Matsubara time via $(l, m = 1, ..., M)$

$$\hat{G}_{lm}(\tau) = -\langle T_\tau \Phi_l(\tau) \Phi^\dagger_m(0) \rangle \ , \tag{18}$$

we can rewrite the retarded Greens function matrix $\hat{G}_{\text{3D}}$ as

$$\hat{G}_{\text{3D}} = \left(\begin{array}{cccc} \hat{G}_{11} & \hat{G}_{12} & \hat{G}_{13} & \\ \hat{G}_{21} & \hat{G}_{22} & \hat{G}_{23} & \dots \\ \hat{G}_{31} & \hat{G}_{32} & \hat{G}_{33} & \\ & \vdots & & \ddots \\ & & & \end{array} \right) . \tag{19}$$

We can now rewrite Eq. (17) using $z = \omega + i\Gamma$ as

$$\left(\begin{array}{cccc} z\hat{I} - \hat{H}_1 & -\hat{T}_{1,2} & & \\ -\hat{T}^{\dagger}{}_{2,1} & z\hat{I} - \hat{H}_2 & -\hat{T}_{2,3} & \\ & -\hat{T}^{\dagger}{}_{3,2} & z\hat{I} - \hat{H}_3 & \ddots \\ & & \ddots & \ddots \\ & & & \end{array} \right) \left(\begin{array}{cccc} \hat{G}_{11} & \hat{G}_{12} & \hat{G}_{13} & \\ \hat{G}_{21} & \hat{G}_{22} & \hat{G}_{23} & \dots \\ \hat{G}_{31} & \hat{G}_{32} & \hat{G}_{33} & \\ & \vdots & & \ddots \\ & & & \end{array} \right) = \hat{I} . \tag{20}$$

By multiplying the lth row of the first matrix with the 1st column of the second matrix, one obtains the following chain of equations, describing the interactions between the nearest-neighboring layers [4] :

$$(z\hat{I} - \hat{H}_1)\hat{G}_{1,1} = \hat{I} + \hat{T}_{1,2}\hat{G}_{2,1} \qquad \text{for } \; l = 1 \tag{21a}$$
$$(z\hat{I} - \hat{H}_2)\hat{G}_{2,1} = \hat{T}^{\dagger}_{2,1}\hat{G}_{1,1} + \hat{T}_{2,3}\hat{G}_{3,1} \qquad \text{for } \; l = 2 \tag{21b}$$
$$(z\hat{I} - \hat{H}_3)\hat{G}_{3,1} = \hat{T}^{\dagger}_{3,2}\hat{G}_{2,1} + \hat{T}_{3,4}\hat{G}_{4,1} \qquad \text{for } \; l = 3 \tag{21c}$$
$$\vdots \tag{21d}$$
$$(z\hat{I} - \hat{H}_l)\hat{G}_{l,1} = \hat{T}^{\dagger}_{l,l-1}\hat{G}_{l-1,1} + \hat{T}_{l,l+1}\hat{G}_{l+1,1} \qquad \text{for } \; l > 1 \tag{21e}$$

Using next $\hat{H}_l = \hat{H}\ \forall l$ and $\hat{T}_{l,l+1} = \hat{T}\ \forall l$, we can rewrite this chain of equations as

$$(z\hat{I} - \hat{\epsilon}_s^{(1)})\hat{G}_{1,1} = \hat{I} + \hat{\alpha}^{(1)}\hat{G}_{2,1} \tag{22a}$$
$$(z\hat{I} - \hat{\epsilon}^{(1)})\hat{G}_{2,1} = \hat{\beta}^{(1)}\hat{G}_{1,1} + \hat{\alpha}^{(1)}\hat{G}_{3,1} \tag{22b}$$
$$(z\hat{I} - \hat{\epsilon}^{(1)})\hat{G}_{3,1} = \hat{\beta}^{(1)}\hat{G}_{2,1} + \hat{\alpha}^{(1)}\hat{G}_{4,1} \tag{22c}$$
$$\vdots$$
$$(z\hat{I} - \hat{\epsilon}^{(1)})\hat{G}_{l,1} = \hat{\beta}^{(1)}\hat{G}_{l-1,1} + \hat{\alpha}^{(1)}\hat{G}_{l+1,1}, \tag{22d}$$

where

$$\hat{\epsilon}_s^{(1)} = \hat{H} \tag{23a}$$
$$\hat{\epsilon}^{(1)} = \hat{H} \tag{23b}$$
$$\hat{\alpha}^{(1)} = \hat{T} \tag{23c}$$
$$\hat{\beta}^{(1)} = \hat{T}^{\dagger} . \tag{23d}$$

By substituting Eq. (22b) into Eq. (22a) and analogously for the rest of the chain of equations, we obtain a new system of equations, decribing the interactions between the 2nd-nearest neighboring layers:

$$(z\hat{I} - \hat{\epsilon}_s^{(2)})\hat{G}_{1,1} = \hat{I} + \hat{\alpha}^{(2)}\hat{G}_{1+2,1} \tag{24a}$$

$$(z\hat{I} - \hat{\epsilon}^{(2)})\hat{G}_{1+2,1} = \hat{\beta}^{(2)}\hat{G}_{1,1} + \hat{\alpha}^{(2)}\hat{G}_{1+4,1} \tag{24b}$$

$$(z\hat{I} - \hat{\epsilon}^{(2)})\hat{G}_{1+4,1} = \hat{\beta}^{(2)}\hat{G}_{1+2,1} + \hat{\alpha}^{(2)}\hat{G}_{1+6,1} \tag{24c}$$

$$\vdots \tag{24d}$$

$$(z\hat{I} - \hat{\epsilon}^{(2)})\hat{G}_{1+2l,1} = \hat{\beta}^{(2)}\hat{G}_{1+2(l-1),1} + \hat{\alpha}^{(2)}\hat{G}_{1+2(l+1),1} \tag{24e}$$

with

$$\begin{aligned}\hat{\epsilon}_s^{(2)} &= \hat{H} + \hat{T}(z\hat{I} - \hat{H})^{-1}\hat{T}^\dagger \\ &= \hat{\epsilon}_s^{(1)} + \hat{\alpha}^{(1)}(z\hat{I} - \hat{\epsilon}^{(1)})^{-1}\hat{\beta}^{(1)}\end{aligned} \tag{25a}$$

$$\begin{aligned}\hat{\epsilon}^{(2)} &= \hat{H} + \hat{T}(z\hat{I} - \hat{H})^{-1}\hat{T}^\dagger + \hat{T}^\dagger(z\hat{I} - \hat{H})^{-1}\hat{T} \\ &= \hat{\epsilon}^{(1)} + \hat{\alpha}^{(1)}(z\hat{I} - \hat{\epsilon}^{(1)})^{-1}\hat{\beta}^{(1)} + \hat{\beta}^{(1)}(z\hat{I} - \hat{\epsilon}^{(1)})^{-1}\hat{\alpha}^{(1)}\end{aligned} \tag{25b}$$

$$\begin{aligned}\hat{\alpha}^{(2)} &= \hat{T}(z\hat{I} - \hat{H})^{-1}\hat{T} \\ &= \hat{\alpha}^{(1)}(z\hat{I} - \hat{\epsilon}^{(1)})^{-1}\hat{\alpha}^{(1)}\end{aligned} \tag{25c}$$

$$\begin{aligned}\hat{\beta}^{(2)} &= \hat{T}^\dagger(z\hat{I} - \hat{H})^{-1}\hat{T}^\dagger \\ &= \hat{\beta}^{(1)}(z\hat{I} - \hat{\epsilon}^{(1)})^{-1}\hat{\beta}^{(1)}\end{aligned} \tag{25d}$$

Repeating the substitution after n iterations, we obtain the following equations describing the interactions between the 2nd-nearest neighboring layers,

$$(z\hat{I} - \hat{\epsilon}_s^{(n)})\hat{G}_{1,1} = \hat{I} + \hat{\alpha}^{(n)}\hat{G}_{1+2^n,1} \qquad l = 0 \tag{26a}$$

$$(z\hat{I} - \hat{\epsilon}^{(n)})\hat{G}_{1+2^n l,1} = \hat{\beta}^{(n)}\hat{G}_{1+2^n(l-1),1} + \hat{\alpha}^{(n)}\hat{G}_{1+2^n(l+1),1} \qquad l > 0 \tag{26b}$$

$$(z\hat{I} - \hat{\epsilon}^{(n)})\hat{G}_{1,1+2^n l} = \hat{\beta}^{(n)}\hat{G}_{1,1+2^n(l-1)} + \hat{\alpha}^{(n)}\hat{G}_{1,1+2^n(l+1)} \qquad l > 0 \tag{26c}$$

This relation can be written in matrix form as:

$$\left(z\hat{I} - \hat{H}_{\text{3D}}^{\text{eff}}\right)\hat{G}_{\text{3D}}^{\text{eff}} = \hat{I} \tag{27}$$

with

$$\hat{H}_{\text{3D}}^{\text{eff}} = \begin{pmatrix} \hat{\epsilon}_s^{(n)} & \hat{\alpha}^{(n)} & & \\ \hat{\beta}^{(n)} & \hat{\epsilon}^{(n)} & \hat{\alpha}^{(n)} & \\ & \hat{\beta}^{(n)} & \ddots & \ddots \\ & & \ddots & \\ & & & \end{pmatrix} \tag{28}$$

being the effective Hamiltonian matrix for the bulk system, and

$$\hat{G}_{3\mathrm{D}}^{\mathrm{eff}} = \begin{pmatrix} \hat{G}_{1,1} & \hat{G}_{1,1+2^n} & \hat{G}_{1,1+2^n*2} & \\ \hat{G}_{1+2^n,1} & \hat{G}_{1+2^n,1+2^n} & \hat{G}_{1+2^n,1+2^n*2} & \cdots \\ \hat{G}_{1+2^n*2,1} & \hat{G}_{1+2^n*2,1+2^n} & \hat{G}_{1+2^n*2,1+2^n*2} & \\ & \vdots & & \ddots \\ & & & \end{pmatrix} \tag{29}$$

being the effective Greens function matrix for the bulk system.

The matrices $\hat{\epsilon}_s^{(n)}$, $\hat{\epsilon}^{(n)}$, $\hat{\alpha}^{(n)}$, $\hat{\beta}^{(n)}$ are defined recursively via

$$\hat{\alpha}^{(n)} = \hat{\alpha}^{(n-1)}(z\hat{I} - \hat{\epsilon}^{(n-1)})^{-1}\hat{\alpha}^{(n-1)} \tag{30a}$$

$$\hat{\beta}^{(n)} = \hat{\beta}^{(n-1)}(z\hat{I} - \hat{\epsilon}^{(n-1)})^{-1}\hat{\beta}^{(n-1)} \tag{30b}$$

$$\hat{\epsilon}_s^{(n)} = \hat{\epsilon}_s^{(n-1)} + \hat{\alpha}^{(n-1)}(z\hat{I} - \hat{\epsilon}^{(n-1)})^{-1}\hat{\beta}^{(n-1)} \tag{30c}$$

$$\hat{\epsilon}^{(n)} = \hat{\epsilon}^{(n-1)} + \hat{\alpha}^{(n-1)}(z\hat{I} - \hat{\epsilon}^{(n-1)})^{-1}\hat{\beta}^{(n-1)} + \hat{\beta}^{(n-1)}(z\hat{I} - \hat{\epsilon}^{(n-1)})^{-1}\hat{\alpha}^{(n-1)} , \tag{30d}$$

where the $\hat{\epsilon}_s^{(n)}$ is the effective Hamiltonian for the bulk top layer , $\hat{\epsilon}^{(n)}$ is the effective Hamiltonian for the bulk layers, and $\hat{\alpha}^{(n)}$ and $\hat{\beta}^{(n)}$ are the effective 2nd-neareast neighbor hoppings between bulk layers.

The above procedure allows us to integrate out all but the top-most bulk layer. In the limit $n \to \infty$ for the number of iterations, the intralayer coupling dominates the interlayer coupling in the effective Hamiltonian Eq. (28) and we obtain

$$\hat{\alpha}^{(n)} \to \hat{0} \tag{31a}$$

$$\hat{\beta}^{(n)} \to \hat{0} \tag{31b}$$

$$(z\hat{I} - \hat{\epsilon}_s^{(n)})^{-1} \to \hat{G}_{1,1} \tag{31c}$$

$$(z\hat{I} - \hat{\epsilon}^{(n)})^{-1} \to \hat{G}_{1+2^n,1+2^n} . \tag{31d}$$

The Greens function matrix for the top layer of the semi-infinite bulk system, $\hat{G}_{\mathrm{tb}}$, is hence given by:

$$\hat{G}_{\mathrm{tb}} = \hat{G}_{11} = (z\hat{I} - \hat{\epsilon}_s^{(n)})^{-1} \tag{32}$$

Coupling of surface and bulk states We can now construct the total Greens function of the surface states and states in the top bulk layer using Dyson's equation:

$$\hat{G}_{\mathrm{total}}^{-1} = \hat{G}_0^{-1} - \hat{H}' \tag{33}$$

where $\hat{G}_0^{-1}$ and $\hat{H}'$ are block matrices defined as:

$$\hat{G}_0 = \begin{pmatrix} \hat{G}_{\rm sc} & 0 \\ 0 & \hat{G}_{\rm tb} \end{pmatrix}; \quad \hat{H}' \; = \; \begin{pmatrix} \hat{H}_{\rm Ag} & \hat{H}_{\rm hyb} \\ \hat{H}^{\dagger}_{\rm hyb} & \hat{H}_{\rm Fe} \end{pmatrix} \tag{34}$$

Here, $\hat{G}_{\rm sc}$ is the Greens function of the surface orbital that acount for the presence of the quantum corral, and $\hat{G}_{\rm tb}$ is the Greens function for the top bulk layer in Eq. (32). $\hat{H}_{\rm Ag}$, $\hat{H}_{\rm Fe}$ and $\hat{H}_{\rm hyb}$ are the matrix forms of the following Hamiltonians

$$H_{\rm Fe} = -J \sum_{\sigma,\sigma'} d^{\dagger}_{\mathbf{R}_{\rm Fe},\sigma} \sigma^{z}_{\sigma\sigma'} d_{\mathbf{R}_{\rm Fe},\sigma'} = \Phi_1^{\dagger} \hat{H}_{\rm Fe} \Phi_1 \tag{35}$$

$$H_{\rm Ag} = -U \sum_{\sigma} f^{\dagger}_{\mathbf{R}_{\rm Ag},\sigma} f_{\mathbf{R}_{\rm Ag},\sigma} = \Psi^{\dagger} \hat{H}_{\rm Ag} \Psi \tag{36}$$

$$H_{\rm hyb} = -V_{\rm hyb} \sum_{\mathbf{r},\sigma} \left(f^{\dagger}_{\mathbf{r},\sigma} d_{\mathbf{r},\sigma} + h.c. \right) = \Psi^{\dagger} \hat{H}_{\rm hyb} \Phi_1 + h.c. \tag{37}$$

where $H_{\rm Fe}$ describes the scattering of electrons in the top-most bulk layer off a magnetic Fe atom located at site $R_{\rm Fe}$ (see Fig. S6B) inside the corral, with J being the magnetic scattering potential, $H_{\rm Ag}$ describes the scattering of surface-state electrons off a nonmagnetic Ag atom located at site $R_{\rm Ag}$ (see Fig. S6B) inside the corral, with U being the nonmagnetic scattering potential, and $H_{\rm hyb}$ represents the hybridization between the surface states and the states in the topmost bulk layer with hybridization strength $V_{\rm hyb}$. Note that for the main text, we defined $H_{\rm at} = H_{\rm Fe} + H_{\rm Ag}$. The LDOS $N(E, \mathbf{r})$ for the total system as a function of energy E and position $\mathbf{r}$ is then given by

$$N(E, \mathbf{r}) = -\frac{1}{\pi} \mathrm{Im} \left[\hat{\mathrm{G}}_{\rm total}(\omega = \mathrm{E}/\hbar) \right]_{\rm ii} \tag{38}$$

where $\left[\hat{G}_{\rm total}(\omega) \right]_{ii}$ is the (ii) element (with i being the site index) of the matrix $\hat{G}_{\rm total}$ corresponding to position $\mathbf{r}$.

To further illuminate the origin of the change in the relative intensity of the β^{+} and β^{-} components of the YSR projections with increasing corral length, as shown in Fig. 5q of the main text (which is here replotted in Fig. S8A) we note that the form of $G_{\rm total}$ in Eq. (33) together with that of $\hat{G}_0$ and $\hat{H}'$ in Eq. (34) implies that both the real and imaginary parts of $\hat{G}_{\rm sc}$ [see Eq. (10)], i.e., the Greens function of the surface describing the existence of corral eigenmodes in the normal state, enters the calculation of the full local density of state $N(E, \mathbf{r})$ in Eq. (38); the latter is employed to compute the LDOS of the YSR projections. This Greens function is highly p-h asymmetric, as shown in Figs. S8B-D where we present the frequency dependence of the real and imaginary parts of $[\hat{G}_{\rm sc}(\omega)]_{\mathbf{r},\mathbf{r}}$ at the site $\mathbf{r}$ of the magnetic impurity inside the corral for three different corral lengths.

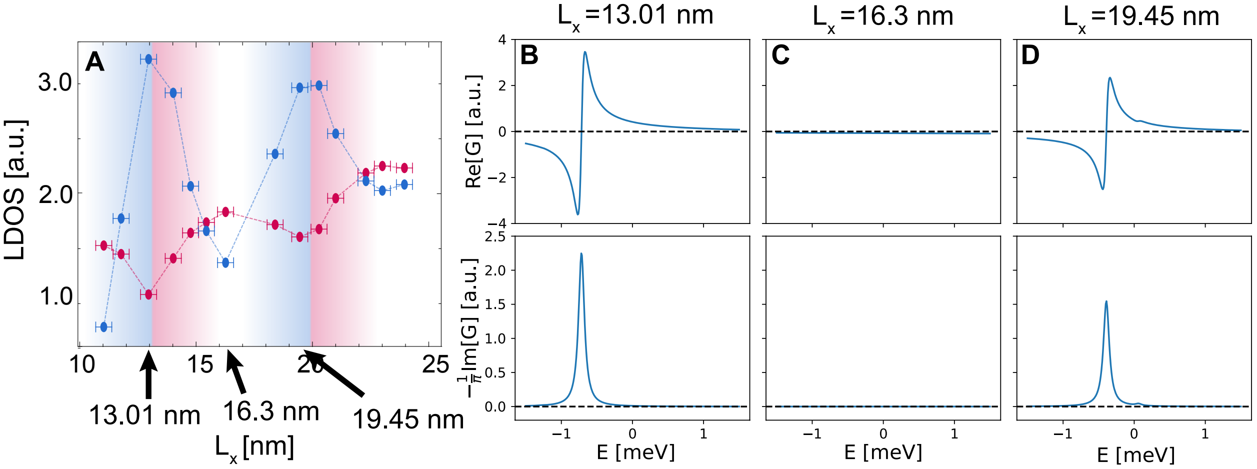

Supplementary Figure 8 | Frequency dependence of the local Greens function of the surface states. (**A**) Fig. 5q from the main text (horizontal errors defined by the deviation of the short-corral side's inner wall atoms from a straight line). (**B**)-(**D**) Frequency dependence of the real (upper row) and imaginary parts (lower row) of the local Greens function of the surface states (in the normal state), $[\hat{G}_{\rm sc}(\omega)]_{\mathbf{r},\mathbf{r}}$, at the site $\mathbf{r}$ of the Fe impurity for three different corral lengths. The corresponding corral lengths are denoted by arrows in (A).

Here, we show the energy range of the corral eigenmodes' Greens functions that is relevant for the emergence of the YSR state. For the case shown in Fig. S8C, the eigenmodes are located significantly away from the Fermi energy, $E_{\rm F}$ (see Fig. S7A), and thus the corral Greens functions is small and featureless near $E_{\rm F}$. In this case, the relative intensity of the β^+ and β^- components of the YSR projections is the same as that of the native YSR state (see arrow in Fig. S8A). In contrast, for the cases shown in Figs. S8B and D the eigenmodes are close to $E_{\rm F}$, giving rise to a large overal scale and significant p-h asymmetry of the corresponding corral Greens functions. In these cases, the relative intensity of the β^+ and β^- components of the YSR projections is inverted with respect to that of the native YSR state (see arrows in Fig. S8A). Since with increasing corral length, the Greens functions shown in Figs. S8B-D are the only components that change in the calculation of the full LDOS [see Eqs. (33), (34) and (38)], we conclude that the change in the relative intensity of the β^+ and β^- components of the YSR projections with increasing corral length is driven by the changing p-h asymmetry of the corral eigenmodes near $E_{\rm F}$. While this is reminiscent of the dependence of the p-h asymmetry of a YSR state arising from a single magnetic impurity on the p-h asymmetry of the conduction band in the normal state [5], in our case, the p-h asymmetry of the YSR state projection arises from the p-h asymmetry of the projecting subsystem, i.e., the corral eigenmodes.

The band parameters for the calculation of the surface LDOS were chosen as follows. (i) $\Delta_0 = 1.35$ meV was chosen to reproduce the experimentally measured position of the coherence peaks; (ii) since no first-principle calculations are available to describe the electronic structure of

the Ag island bulk states, we chose $t_{\rm b} = 85$ meV, $\mu_{\rm b} = -510$ meV, and $t_\perp = 85$ meV, in order to guarantee that despite the finite number of sites in the Ag island that we consider, the bulk Greens functions possess a smooth form in energy; (iii) The magnetic scattering potential of the Fe adatom $J = 335$ meV was chosen to reproduce the experimental energy of the β YSR state; (iv) $V_{\rm hyb} = 22$ meV was chosen to reproduce the experimentally observed dependence of the intensities of the projected β^+ and β^- branches on the corral length (see also discussion below). As we will discuss below, the results presented in the main text vary only very weakly with the specific values chosen for $t_{\rm b}$ and $\mu_{\rm b}$, as long as J and $V_{\rm hyb}$ are adjusted accordingly. Finally, the spatial LDOS plots shown in the main text were obtained for $\Gamma = 0.05 t_e$ and for a system size of $N_x = 128 a_0$ and $N_y = 64 a_0$ on a hexagonal lattice with periodic boundary conditions. Finally, to obtain the intensities of the $\beta^\pm$ branches shown in Fig. 5q of the main text, we integrated the theoretical LDOS at energies $\pm E_\beta$ over the region inside corral indicated in Fig. S6B.

Evidence for the dominant coupling of the Fe impurity to the Ag bulk band, rather than to the Ag surface band, comes from a comparison of the resulting YSR state energies, as shown in Fig. S9. Specifically, considering the system discussed above and described in the main text, where superconductivity is proximity induced into the surface band (which contains the quantum corral) by the Ag bulk band, we contrast two scenarios. In the first scenario, the Fe moment couples to the Ag bulk band (see Fig. S9A); this is the scenario considered in the main text. In this case, the energy of the YSR state is constant as a function of the corral length (within the theoretical energy resolution, see Fig. S9B), in agreement with the experimental observations (see Figs. S23A and S24). In the second scenario, we assume that the Fe moment couples to the surface band (see Fig. S9B), implying that Eq.(35) is replaced by

$$H_{\rm Fe} = -J \sum_{\sigma,\sigma'} f^\dagger_{{\bf R}_{\rm Fe},\sigma} \sigma^z_{\sigma\sigma'} f_{{\bf R}_{\rm Fe},\sigma'} = \Psi^\dagger \hat{H}_{\rm Fe} \Psi \tag{39}$$

In this case, the YSR state energy varies significantly with the corral length (see Fig. S9D), in contrast to the experimental observation. We, therefore, conclude that the Fe moment couples dominantly to the Ag bulk band, as discussed in the main text.

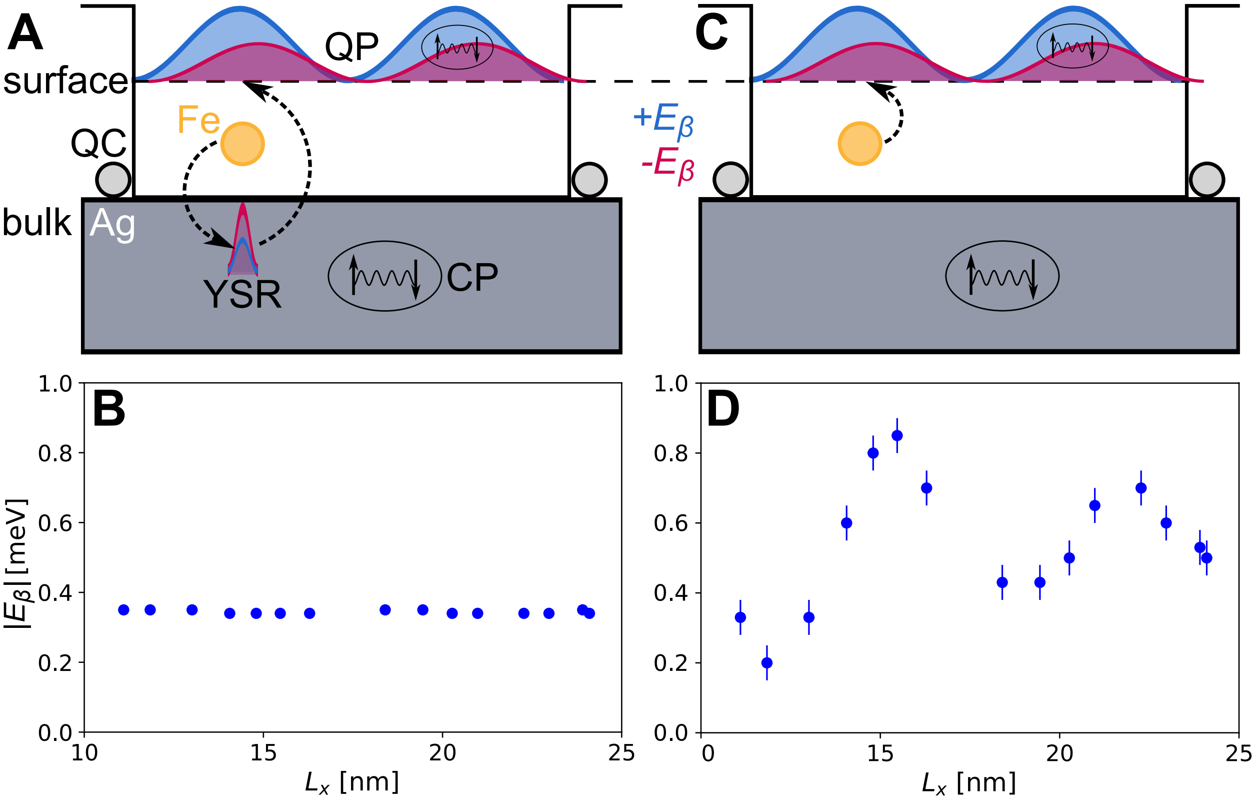

Supplementary Figure 9 | Energy of the YSR state as a function of corral length L_x. (**A**) Schematic for the case when the Fe moment couples to the Ag bulk band, and (**B**) the resulting YSR state energy as a function of corral length ($J = 335$ meV). (**C**) Schematic for the case when the Fe moment couples to the Ag surface band, and (**D**) the resulting YSR state energy as a function of corral length ($J = 1617$ meV). The vertical error bars are shown in solid blue lines and are determined by the energy step size (0.01 meV for panel **B** and 0.05 meV for panel **C**).

Energies of the Machida-Shibata states We next discuss how the energy position of the Machida Shibata states (MSSs) varies with the corral length. These states emerge inside the superconducting gap when the corral eigenmodes (in the normal state) cross the Fermi energy, E_{F}. In Fig. S10A we present the energies of the $(2,1)$ and $(3,1)$ eigenmodes as a function of the corral length; these are the only two eigenmodes that cross E_{F} for the corral lengths considered in the main text. In Figs. S10B-D and Figs. S10E-G we present the energy-dependent LDOS near the coherence peaks located at $E = 1.35$ meV (see vertical gray dashed lines) for corral lengths when the $(2,1)$ and $(3,1)$ eigenmodes are close to E_{F}. We see for all cases, that an additional peak reflecting the existence of the MSS appears around 1.2 meV. This peak possesses the lowest energy (see vertical blue dashed lines) when the eigenmode (in the normal state) is closest to E_{F}, which is the case for the $(2,1)$ mode in the corral with $L_x = 13.01$ (see Fig. S10C), and for the $(3,1)$ mode in the corral with $L_x = 19.45$ nm (see Fig. S10F), in good agreement with the experimental observations shown in Fig. S16.

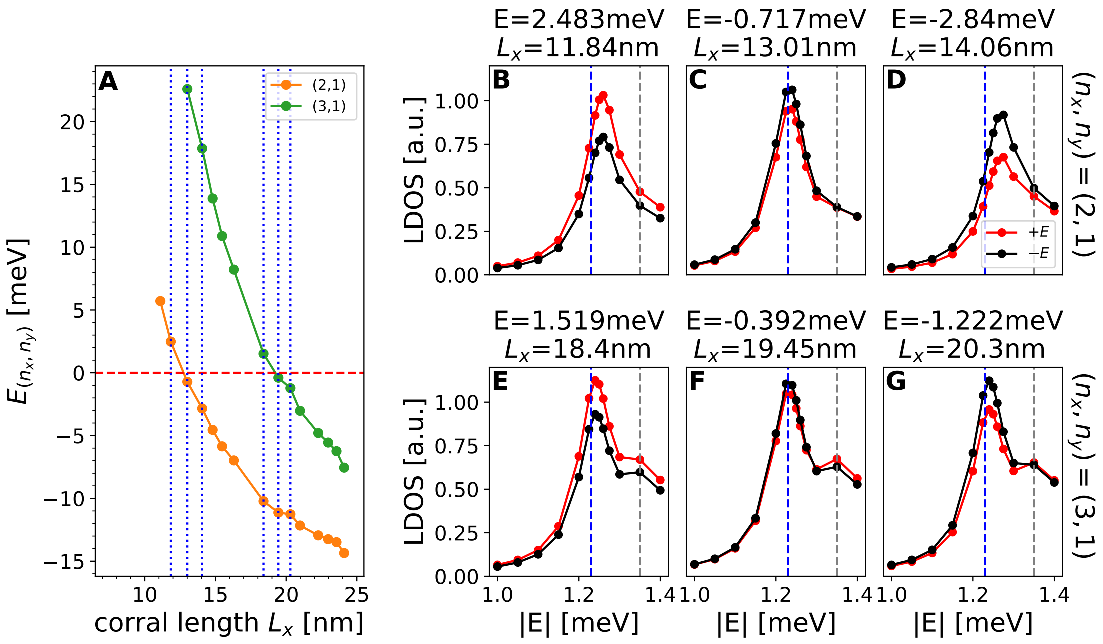

Supplementary Figure 10 | MSSs as a function of corral length. (**A**) Energies of the $(2,1)$ (orange line) and $(3,1)$ (green line) eigenmodes as a function of corral length. The red dashed horizontal line represents the Fermi energy. (**B** to **G**) DOS as a function of $|E|$ for positive energies (red line) and negative energies (black line) for corral lengths $L_x = 11.84$ nm, $L_x = 13.01$ nm, and $L_x = 14.06$ nm for which the $(2,1)$ mode is closest to E_{F} (**B** to **D**) and for $L_x = 18.4$ nm, $L_x = 19.45$ nm, and $L_x = 20.3$ nm for which the $(3,1)$ mode is closest to E_{F} (**E** to **G**), respectively. The corral lengths in **B**-**G** correspond to the vertical blue dashed lines in panel **A**. The vertical gray dashed lines represent the energy of the superconducting coherence peak at $|E| = 1.35$ meV. The vertical blue dashed line represents the lowest energy of the MSS (observed in panels **C** and **F**). The LDOS is obtained by integrating over all sites inside the corral.

Dependence of the YSR projection on the hybridization between the surface and bulk states

We demonstrated in Fig. 5q of the main text that the intensity of the β^+ component of the YSR projection is enhanced over that of the β^- component every time when an eigenmode is close to E_{F}, in good agreement with the experimental findings shown in Fig. 5p. To gain more insight into the origin of this behavior, we present in Fig. S11 the equivalent of Fig. 5q – the integrated DOS for the β^+- and β^--components of the YSR projection as a function of the corral length – for different values of the hybridization, V_{hyb}, between the surface and bulk states.

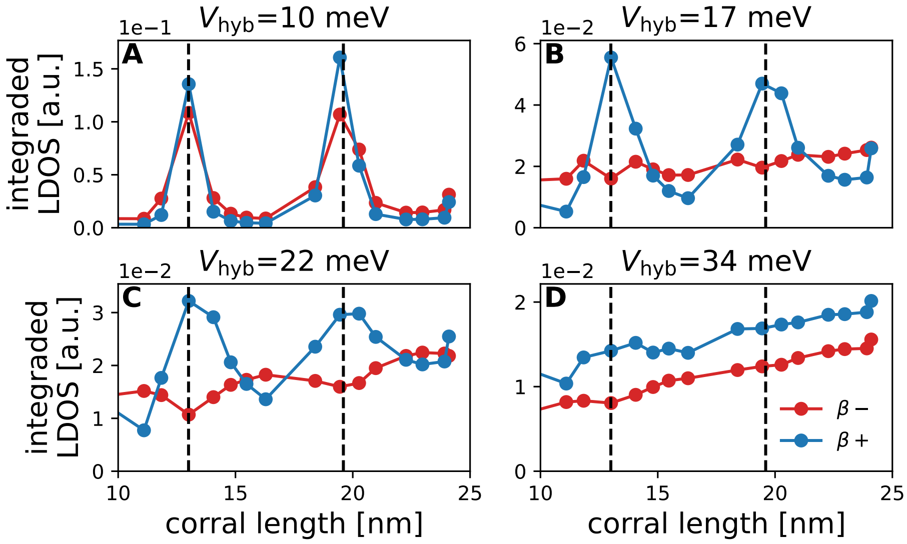

Supplementary Figure 11 | LDOS as a function of corral length. (**A** to **D**) LDOS integrated inside the corral (as shown in Fig. S6B) as a function of corral length for V_{hyb} =10 meV, 17 meV, 22 meV, and 34 meV at $E = \pm E_\beta = \pm 0.33$ meV. Black dashed lines represent the corrals for which the eigenmodes are closest to E_{F}.

For weak hybridization, $V_{\mathrm{hyb}} = 10$ meV (Fig. S11A), we find that the intensities of both components increase when the eigenmodes are close to E_{F}, although the β^+-component is more strongly enhanced. Increasing V_{hyb} further to $V_{\mathrm{hyb}} = 17$ meV (Fig. S11B) and $V_{\mathrm{hyb}} = 22$ meV (Fig. S11C), we find that only the β^+-component is enhanced, while the β^--component is suppressed. For even larger $V_{\mathrm{hyb}} = 34$ meV (Fig. S11D), the intensities of both components become approximately equal. We find that the overall scale of the intensities decreases with increasing V_{hyb}.

In Fig. S12, we plot the energy-dependent and spatially integrated DOS on the surface for several values of V_{hyb} and corral length. The in-gap peak in the DOS reflects the existence of the MSS, and we find, as expected, that this peak moves to higher energies with increasing V_{hyb}. Note, that, for the two qualitatively different results shown in Figs. S11A and B, the lowest energy of the MSSs is still significantly higher than the energy of the YSR state at $E = \pm 0.33$ meV.

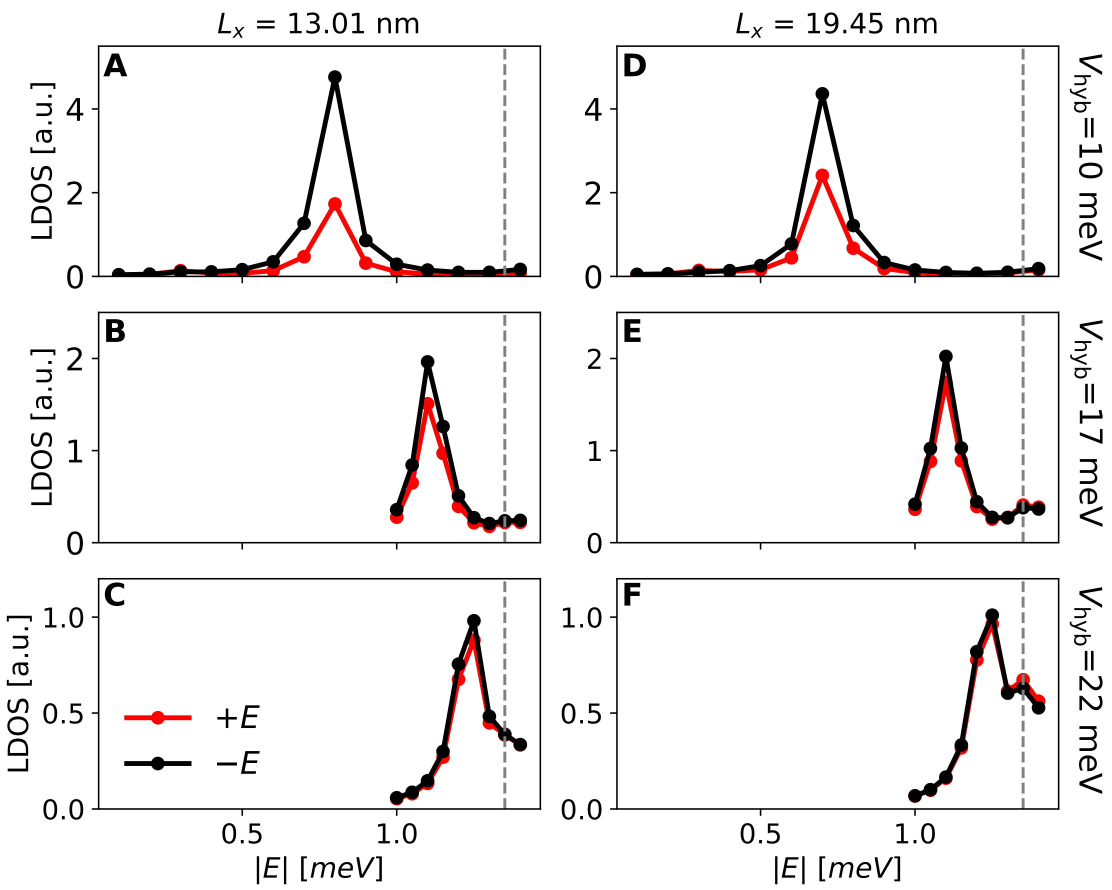

Supplementary Figure 12 | MSSs for different parameters. (**A** to **C**) DOS as a function of $|E|$ for positive energies (red line) and negative energies (black line) for corral length $L_x = 13.01$ nm for V_{hyb}=10 meV, V_{hyb}=17 meV, and V_{hyb}=22 meV, respectively. (**D** to **F**) DOS as a function of $|E|$ for corral length $L_x = 19.45$ nm for V_{hyb}=10 meV, V_{hyb}=17 meV, and V_{hyb}=22 meV, respectively. The vertical gray dashed lines represent the energy of the superconducting coherence peak at $|E| = 1.35$ meV. The DOS is obtained by integrating over all sites inside the corral.

Intensity inversion across a phase transition It is well known that there exists a critical value of the magnetic exchange, J_c, at which the YSR state branches cross zero energy, a crossing that signifies a local phase transition in which the superconducting ground state changes from a singlet to a doublet [5]. An important question has therefore been, whether it is possible to experimentally determine on which side of this phase transition a YSR state might be located. While we cannot answer this question in a definite form, we here note that the relative intensity between the β^-- and β^+-branches is inverted at the phase transition.

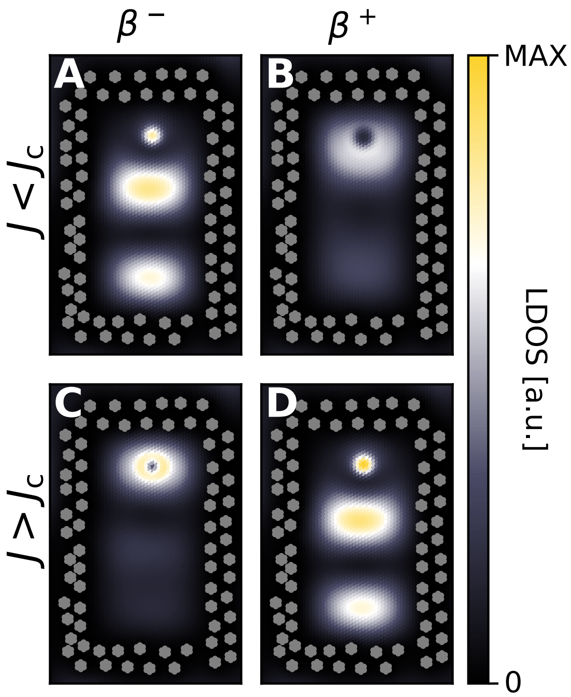

Supplementary Figure 13 | Variation of the p-h composition across a phase transition. Spatial LDOS of the β^- and β^+ components, respectively, for $J < J_\mathrm{c}$ (**A** and **B**) and for $J > J_\mathrm{c}$ (**C** and **D**), for a corral of length $L_x = 16.3$ nm. $J = 335$ meV and $J = 397$ meV are chosen, at which the energy of the YSR state is at $E = \pm 0.33$ meV.

To demonstrate this, we present in Fig. S13 a comparison of the spatial LDOS plots of the β^-- and β^+-branches at $\pm E_\beta$ for $J < J_c$ (first row) and $J > J_c$ (second row). This comparison clearly shows an inversion in the relative intensities, which thus provides another means to manipulate the p-h composition of the YSR state projection.

Dependence of the projected image on the bulk parameters of the Ag island We find that the theoretical results presented in Fig. 5 of the main text, and in particular the dependence of the integrated intensities of the β^+ and β^- components on the corral lengths shown in Fig. 5q, vary only very weakly with the specific values taken for t_b and μ_b. To demonstrate this, we increase the values of t_b and μ_b by a factor of 4 (parameter set 2) from the original parameters (parameter set 1), and adjusted the values of J and V_hyb accordingly to $J = 1340$ meV and $V_\mathrm{hyb} = 44$ meV. We find that the resulting dependence of the intensities of the projected β^+ and β^- branches on the corral length hardly change, as follows from the comparison of the result in Fig. S14A. Moreover,

also the positions of the MSSs are hardly affected by the change in the parameters, as shown in Figs. S14B and C. We, therefore, conclude that the results shown in the main text are robust against changes in the band parameter of the bulk Ag island.

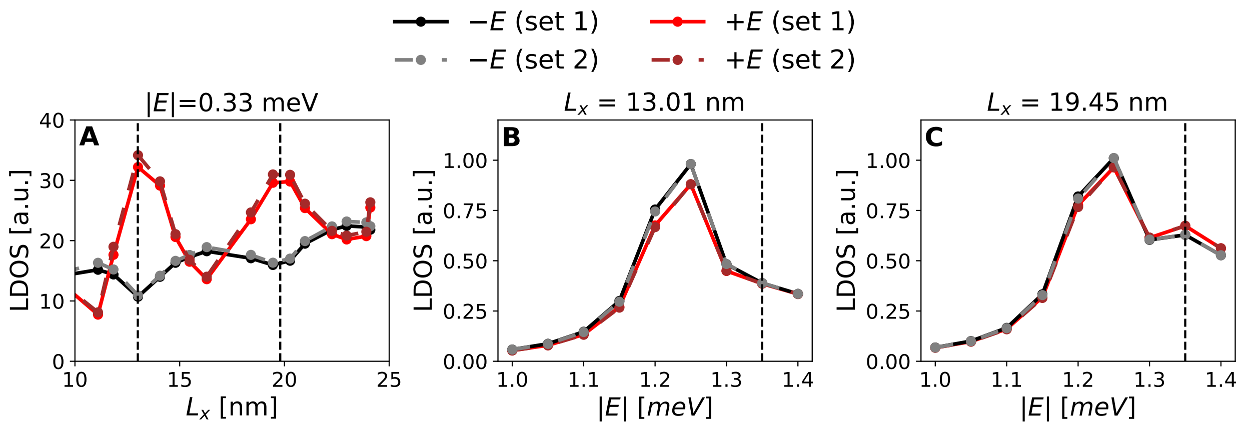

Supplementary Figure 14 | Effect of change in Ag bulk parameters. (**A**) Integrated LDOS inside the corral (as shown in Fig. S6B) as a function of the corral length at $E = \pm 0.33$ meV for parameter set 1 with t_b = 85 meV, $\mu_b = -510$ meV, $V_{\mathrm{hyb}} = 22$ meV, $J = 335$ meV (solid line) and for parameter set 2 with t_b = 340 meV, $\mu_b = -2040$ meV, $V_{\mathrm{hyb}} = 44$ meV, $J = 1340$ meV (dashed line). Black dashed vertical lines represent the corrals for which the eigenmodes are closest to E_{F}. (**B** and **C**) MSSs for set 1 (solid line) and for set 2 (dashed line) for the corrals of length $L_x = 13.01$ nm and $L_x = 19.45$ nm, respectively. The vertical black dashed lines represent the energy of the superconducting coherence peak at $|E| = 1.35$ meV.

YSR projection in an elliptical corral with a Fe adatom located at its center In Fig. S15 we present the spatial LDOS in an elliptical corral with a magnetic Fe adatom located at its center, for the β^- (Fig. S15A) and β^+ components (Fig. S15B) of the YSR state. Since the corral eigenmode closest to E_{F} possesses spectral weight at the center site, we find that an image of the YSR state is projected to the site of the other two maxima of the corral's eigenmodes, as is particularly evident for the β^+ component.

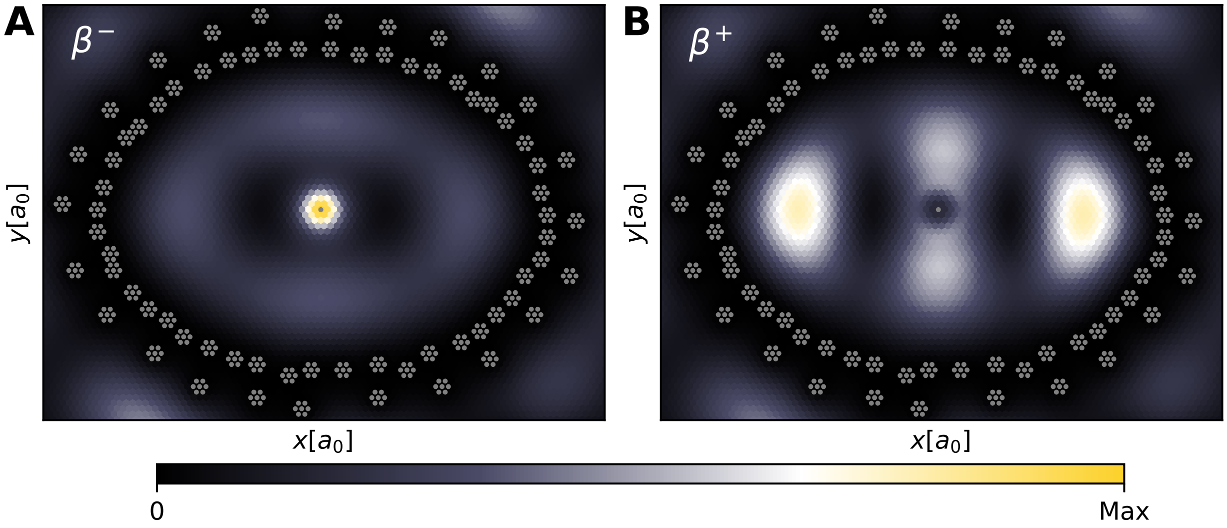

Supplementary Figure 15 | Elliptical corral with a magnetic impurity located at its center. Theroretical results for spatial LDOS in an elliptical corral with an Fe adatom located at its center for the (**A**) β^- and (**B**) β^+ components of the YSR state.

Further improvements of the theoretical model To further improve the already good agreement between the theoretical and experimental results shown in Fig. 5 of the main text, one could include several new aspects in the Hamiltonian used in the main text. In particular, so far, we have only considered a local magnetic scattering potential of the Fe adatom, while in general the adatom could also possess a non-magnetic scattering potential. Moreover, the magnetic or non-magnetic scattering potentials could possess an extended spatial form, and the detailed orbital structure could also play a role in the spatial structure of the $\beta^{\pm}$ components. A repulsive non-magnetic scattering potential would move spectral weight of the $\beta^{\pm}$ components of the YSR projection away from the site of the adatom, which could further improve the agreement with the experimental results in Fig. 5. However, we did not include these aspects in our current study as they would lead to additional parameters into which we currently do not have insight from experiment.

Supplementary Note 4 Determination of the corral length dependent eigenmode energies

As described before [2], the development of the eigenmode energies $E(n_x, n_y)$ of the corral with length L_x and width L_y usually can be determined to first approximation by fitting averaged $\mathrm{d}I/\mathrm{d}V$ spectra (Fig. S16), obtained from the $\mathrm{d}I/\mathrm{d}V$ line profiles taken along the vertical longitudinal axis inside each corral (Fig. S17, 2nd column), as a function of their L_x (fixed L_y), to the hard-wall particle-in-a-box model:

$$E(n_x, n_y) = \frac{\hbar^2}{2m_{\text{eff}}} \left[\left(\frac{\pi n_x}{(L_x - \delta_x)} \right)^2 + \left(\frac{\pi n_y}{(L_y - \delta_y)} \right)^2 \right] + E_0. \tag{40}$$

In order to omit the short-range decay of the native β YSR state around the Fe atom contained on the longitudinal corral axis, we cut out a region of $\pm 1.5\,\text{nm}$ around the Fe atom from the $\mathrm{d}I/\mathrm{d}V$ line profiles before calculating the average spectrum for each corral length (Supplementary Note 7 and Fig. S22). In Fig. S16 we show these averaged $\mathrm{d}I/\mathrm{d}V$ spectra as a function of corral length L_x (fixed $L_y = 9.1\,\text{nm}$) with color scales that emphasize the MSSs (Fig. S16A), the corral eigenmode outside the gap region (Fig. S16B) as well as the β YSR quantum projection (Fig. S16C). For assigning the corral eigenmodes and MSSs visible in Fig. S16 to their quantum numbers n_x (fixed $n_y = 1$), we analyse the corresponding spatially resolved $\mathrm{d}I/\mathrm{d}V$ line profiles of each of the corrals (Fig. S17, 2nd column) at positive and negative bias voltage outside of the gap (Fig. S17, 3rd column), at the particle and hole partners of the β YSR quantum projection (Fig. S17, 4th column) and at the minimal energies of the MSSs inside the gap (Fig. S17, 5th column), respectively. From these figures, we can see, e.g., that the corral eigenmode with three maxima along x ($n_x = 3$) first appears for the $L_x = 22.97\,\text{nm}$ corral with a large intensity outside the gap at *negative* $V_{\text{bias}} = -4.5\,\text{mV}$ and in the *negative* energy β^- YSR projection, then for the $L_x = 20.98\,\text{nm}$ corral in the *negative* energy MSS, then for the $L_x = 20.27\,\text{nm}$ corral in the *positive* energy β^+ YSR projection (while the *negative* energy β^- YSR projection has small intensity), then for the $L_x = 19.45\,\text{nm}$ corral in the *positive* energy MSS, and finally for the $L_x = 18.4\,\text{nm}$ corral again in the *negative* energy β^- YSR projection and outside the gap at *positive* $V_{\text{bias}} = 4.5\,\text{mV}$. This sequence of states corroborates our conclusions from Fig. 5p of the main text on the p-h composition of the β YSR quantum projection. In a similar way the evolution of the $n_x = 2$ and $n_x = 1$ states can be traced. This way, we can assign the MSSs in Fig. S16A, the corral eigenmode intensities outside the gap in Fig. S16B, and the β YSR projections in Fig. S16C to the different n_x as indicated by the colored dashed lines. Next, using Fig. S16A, we determined the corral lengths, for which the $n_x = 1, 2, 3$ MSSs have their minimal energies, resulting in $L_x = 7.25\,\text{nm}$, $14\,\text{nm}$, and $20.12\,\text{nm}$, respectively. For these corral lengths, the respective corral eigenmodes are closest to E_{F} [2], $E(n_x, 1) \approx E_{\text{F}}$. Using the following parameters, which have been determined by fitting the experimental averaged $\mathrm{d}I/\mathrm{d}V$ line profiles versus L_x for the corrals without Fe atoms [2] ($\delta_y = -0.28\,\text{nm}$, $E_0 = -26.4\,\text{meV}$, $m_{\text{eff}} = 0.58 m_e$), we vary δ_x until the Fermi level crossings of Eq. (40) are at the lengths L_x determined above from the minimal MSS energies. The resulting parameters are $\delta_x = 1.4\,\text{nm}$ ($n_x = 1$), $2.3\,\text{nm}$ ($n_x = 2$), $2.6\,\text{nm}$ ($n_x = 3$), and $2.4\,\text{nm}$ ($n_x = 4$). Note, that the sign change of the parameter δ_x with respect to the negative value $\delta_x = -0.28\,\text{nm}$ resulting for the corrals without Fe atoms [2] reflects the reduced effective length of the corral due

to the Fe atom which acts as an additional scattering center.

The resulting $E(n_x, 1)$ given by the dashed lines in Fig. S16 reproduce the experimental dependence of the eigenmode energies on L_x reasonably well also outside of the gap (Fig. S16B). The corresponding lengths L_x, where $E(n_x, 1) = E_\mathrm{F}$, are given by the arrows in Fig. S19 as well as by the sharp transitions between the blue and red shaded areas in Fig. 5p of the main text.

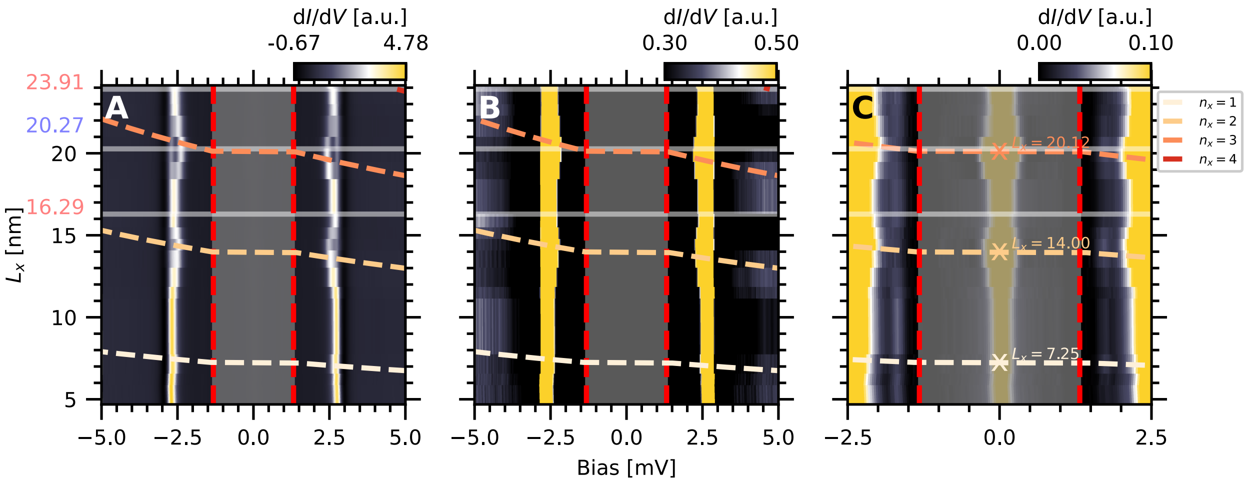

Supplementary Figure 16 | Determinantion of L_x-dependent corral eigenenergies.
(**A** to **C**) Averaged $\mathrm{d}I/\mathrm{d}V$ spectra taken inside all corrals containing the Fe atom as a function of their length $L_x = 4.7\,\mathrm{nm}$ to $L_x = 24.1\,\mathrm{nm}$ ($L_y = 9.1\,\mathrm{nm}$, see Fig. S19 for the corrals). For each corral the average $\mathrm{d}I/\mathrm{d}V$ spectrum was calculated from the $\mathrm{d}I/\mathrm{d}V$ line profiles taken along the longitudinal vertical axis of the corral (same data as in Fig. 4e of the main text, and Figs. S18D, S17, and S22C) by averaging over all x, but excluding a region around the Fe atom as shown in Figs. S22B and S22C. The color scale has been optimized to accentuate the MSSs (A) and the β YSR states (C) inside the gap region, and the corral eigenmodes outside the gap region (B). The colored, diagonal dashed lines show the L_x-dependent evolution of the eigenmode energies with $(n_x, n_y) = (1,1), (2,1), (3,1), (4,1)$ (see legend in C) obtained from fitting the MSSs and averaged $\mathrm{d}I/\mathrm{d}V$ spectra at energies outside the gap (see Supplementary Note 4, fit parameters: $\delta_x = 1.4\,\mathrm{nm}$ ($n_x = 1$), $2.3\,\mathrm{nm}$ ($n_x = 2$), $2.6\,\mathrm{nm}$ ($n_x = 3$), $2.4\,\mathrm{nm}$ ($n_x = 4$), and $\delta_y = -0.28\,\mathrm{nm}$, $E_0 = -26.4\,\mathrm{meV}$, $m_\mathrm{eff} = 0.58 m_e$). They cross E_F at the lengths given by the colored crosses in (C), which are also indicated in Fig. S19 and Fig. 5p of the main text. The white horizontal lines with indicated lengths mark the corrals shown in Figs. 5a to o of the main text.

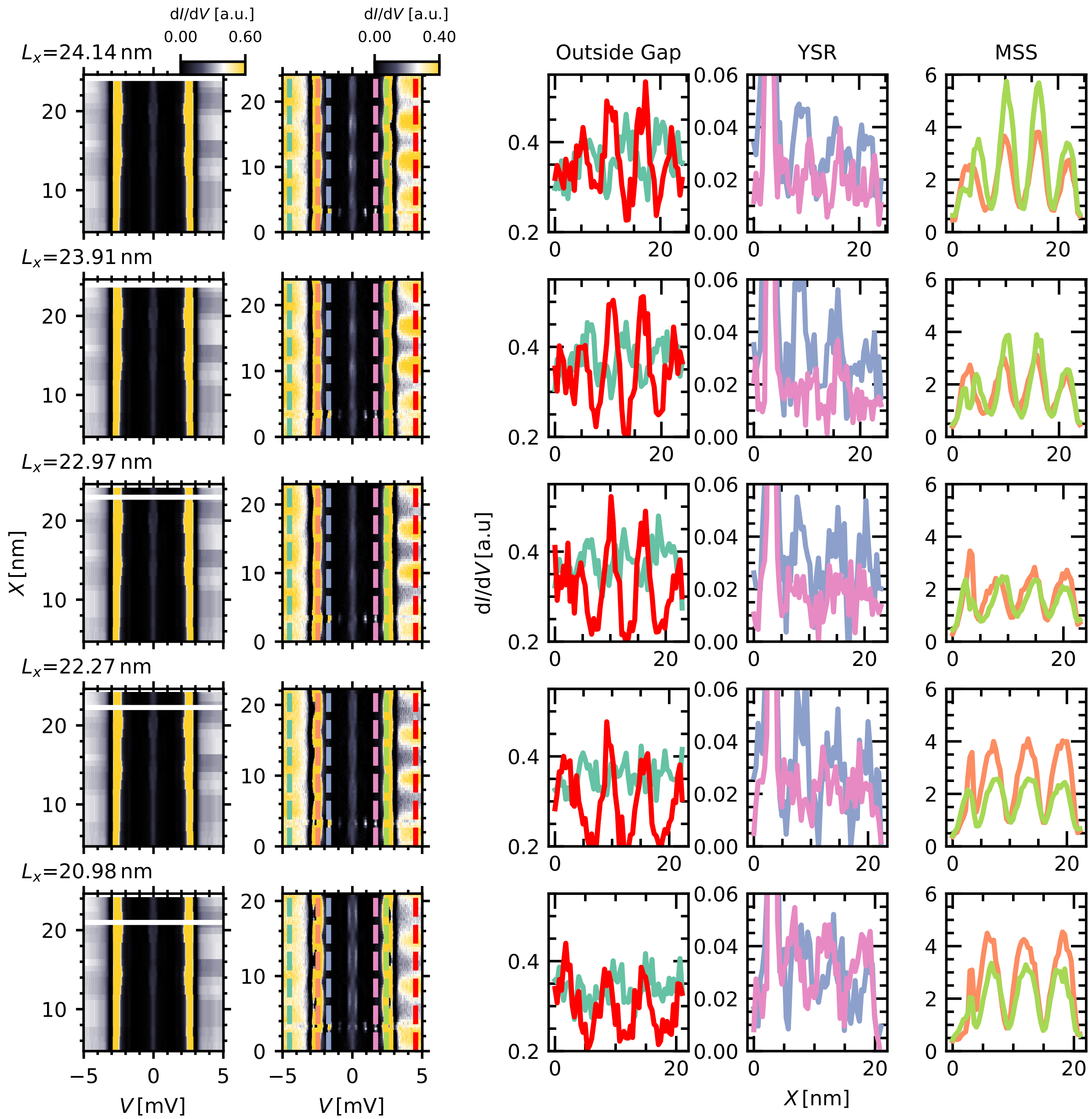

dI/dV [a.u.]
0.00 0.60
0.00 0.40
L_x=24.14 nm
L_x=23.91 nm
L_x=22.97 nm
L_x=22.27 nm
L_x=20.98 nm
X [nm]
V [mV]
Outside Gap
YSR
MSS
dI/dV [a.u]
X [nm]

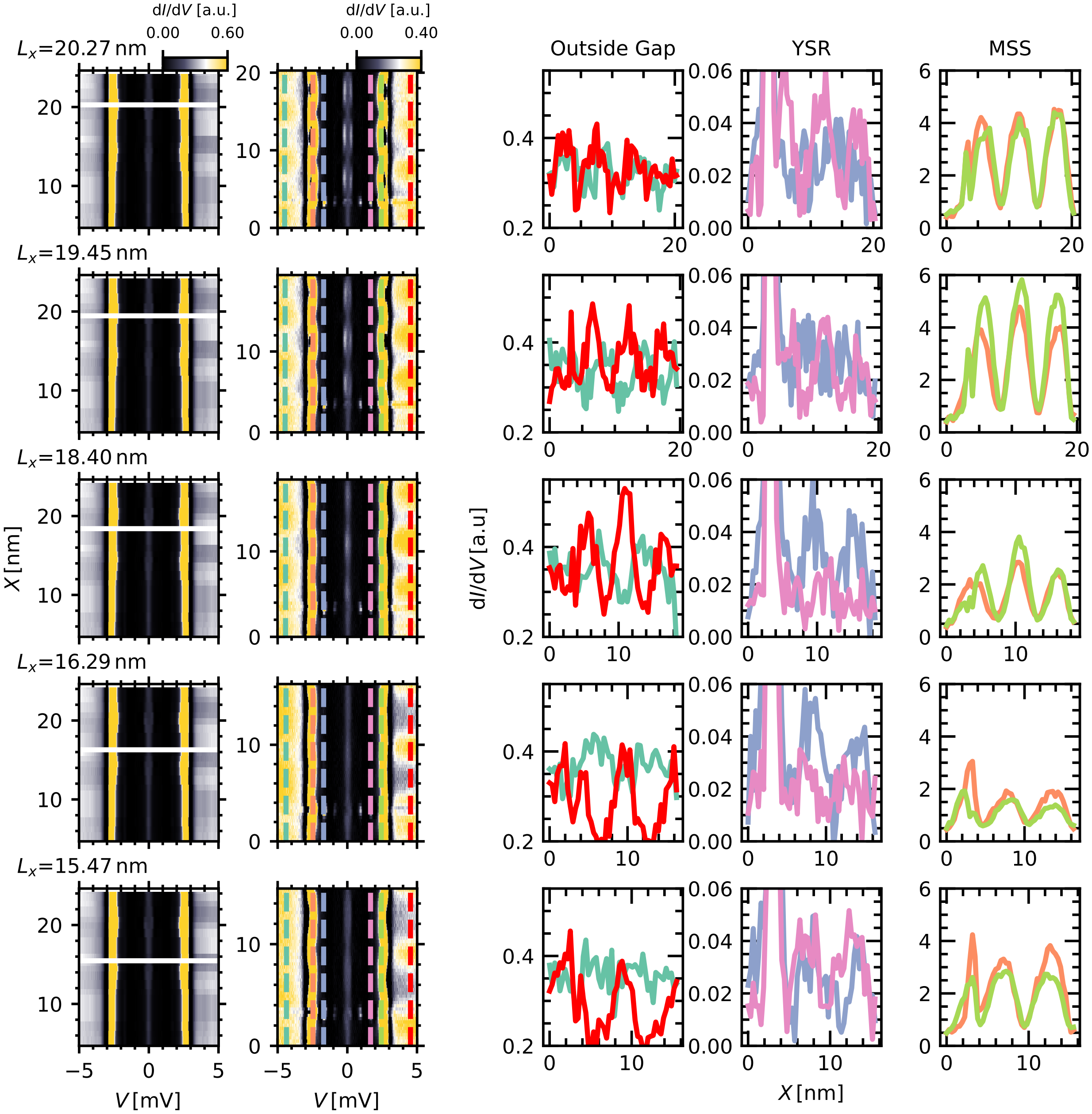
dI/dV [a.u.]
0.00
0.60
0.40
L_x=20.27 nm
L_x=19.45 nm
L_x=18.40 nm
L_x=16.29 nm
L_x=15.47 nm
X [nm]
V [mV]
Outside Gap
YSR
MSS
dI/dV [a.u]
X [nm]

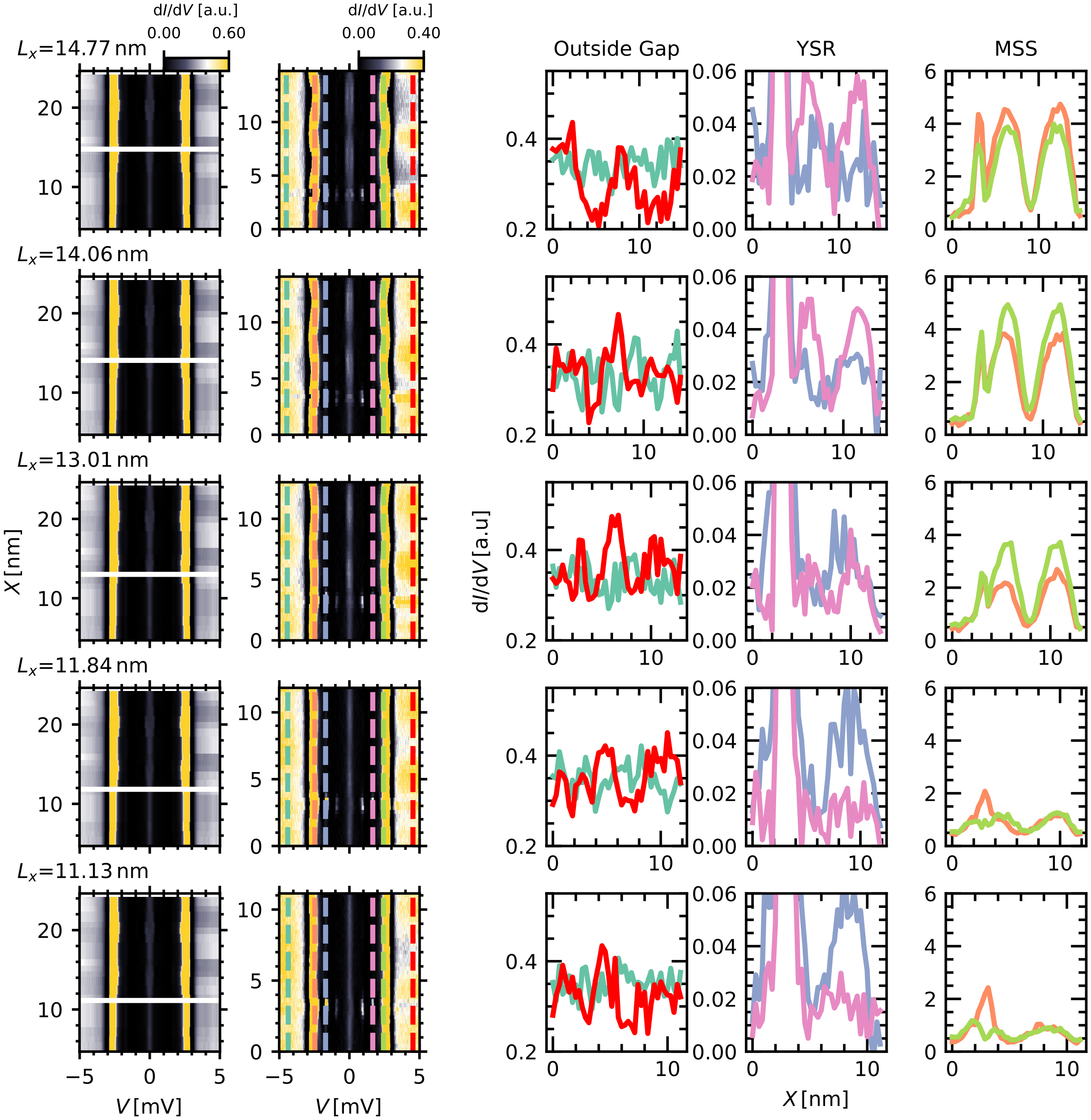
dI/dV [a.u.]
0.00
0.60
dI/dV [a.u.]
0.00
0.40
L_x=14.77 nm
L_x=14.06 nm
L_x=13.01 nm
L_x=11.84 nm
L_x=11.13 nm
X [nm]
V [mV]
V [mV]
Outside Gap
YSR
MSS
dI/dV [a.u]
X [nm]

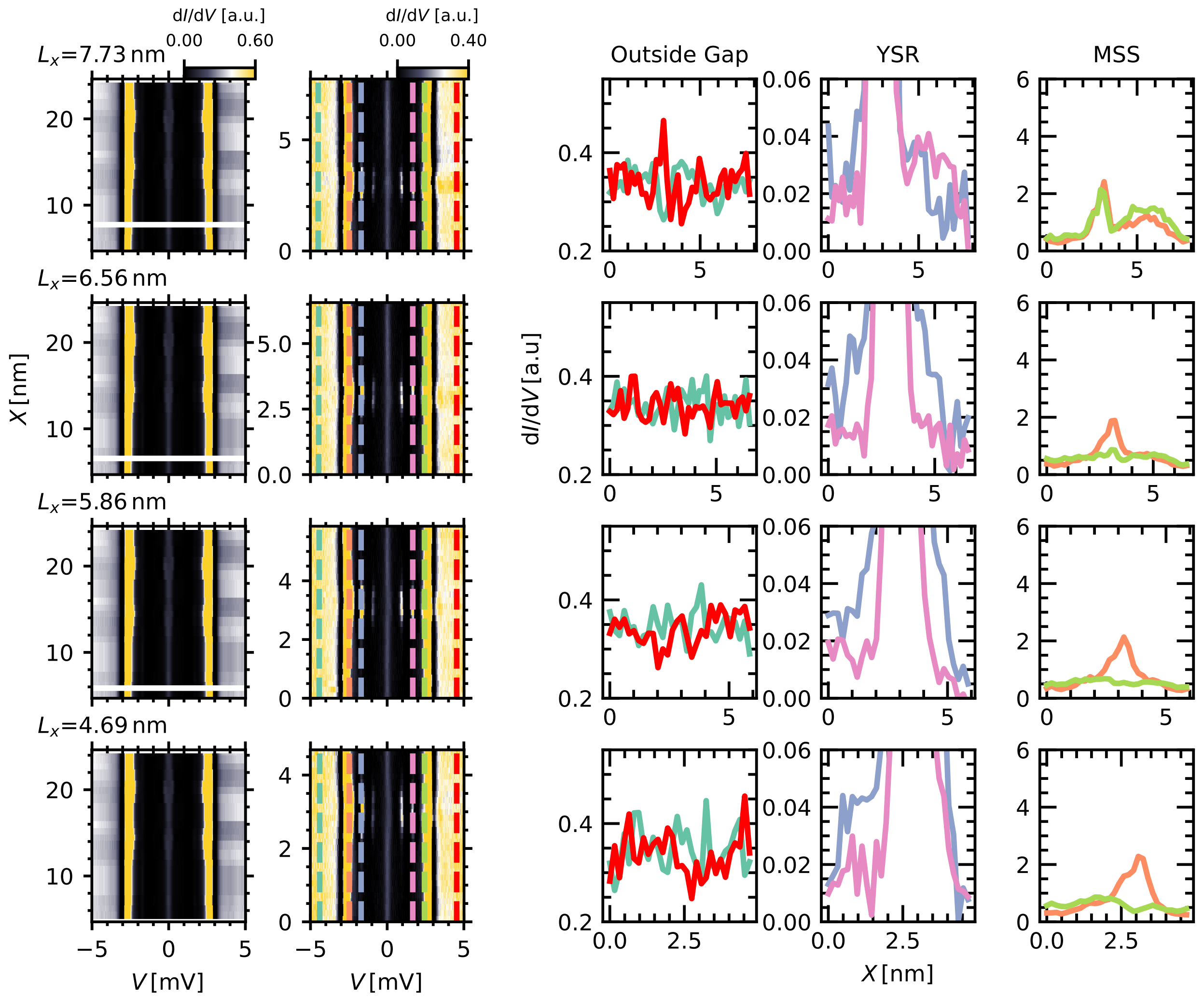

Supplementary Figure 17 | Spatially resolved spectral intensities of corral eigenmodes, of MSSs and of YSR quantum projections. (**1st column**) Averaged $\mathrm{d}I/\mathrm{d}V$ spectrum from Fig. S16 with a white line at the corral length L_x analyzed in the corresponding row and indicated above each panel. (**2nd column**) $\mathrm{d}I/\mathrm{d}V$ line profiles through the longitudinal vertical axis of each of the 19 corrals containing the Fe atom (measurement parameters as in Fig. 4e of the main text). Note, that the corrals are upside down with respect to Figs. 4 and 5 of the main text such that the Fe atoms are always located at $x \approx 2.5\,\mathrm{nm}$. Colored dashed vertical lines, see below. (**3rd column** to **5th column**) Spatially-resolved spectral intensity extracted from the $\mathrm{d}I/\mathrm{d}V$ line profile given in the 2nd column (same row) of the corral eigenmodes at positive and negative bias slightly outside of the gap (3rd column), and of the particle-hole-partner YSR quantum projections (4th column) and MSSs (5th column). The bias voltages are indicated by the correspondingly colored dashed lines in the 2nd column.

Supplementary Note 5 $\mathrm{d}I/\mathrm{d}V$ spectra in rectangular corrals with Ag and Fe atoms

Figure S18 shows the extended data of the rectangular Ag quantum corral with a Ag (Fig. S18A) and a Fe atom (Fig. S18B) in the top quarter, which is also shown in Fig. 4 of the main text. The width and length of the corral measured from the distances of the inner Ag atom rows on opposing sides are $L_y = 9.1\,\mathrm{nm}$ and $L_x = 22.26\,\mathrm{nm}$, respectively. Figures S18C and S18D show the corresponding $\mathrm{d}I/\mathrm{d}V$ line profiles from Figs. 4f and 4e of the main text, respectively, but across the full bias voltage range and without cutting out the gray regions between the red dashed vertical lines marking $\pm\Delta_\mathrm{t}/e$. Moreover, using a larger $\mathrm{d}I/\mathrm{d}V$ value range, the MSSs splitting off from the de Gennes-Saint James coherence peaks are clearly visible at bias voltages of $V_\mathrm{bias} \approx \pm 2.5\,\mathrm{mV}$, modulating in intensity as a function of the position x [2]. The spatial shape and energy of these MSSs is indistinguishable between the two corrals containing the Ag (Fig. S18C) and the Fe atom (Fig. S18D), suggesting that they are not strongly influenced neither by the different electronic configurations of the two atomic species nor by their strongly different spin-related properties.

Single point $\mathrm{d}I/\mathrm{d}V$ spectra taken at the four locations indicated by numbers in Figs. S18A and S18B are presented in Figs. S18E and S18F, i.e., on the Ag atom (1) and an empty spot (2) of the first corral as well as on the Fe atom (3) and an empty spot (4) of the second corral. They are plotted in two different bias voltage ranges in order to emphasize the MSS and the YSR states (Fig. S18E) and the YSR quantum projection (Fig. S18F), respectively. In Fig. S18E, the MSS is visible in all four spectra around $\pm 2.5\,\mathrm{mV}$. Furthermore, the spectrum taken on the Fe atom (3) reveals the intense $\beta^\pm$ YSR peaks well separated from all other spectroscopic features (gray vertical line), the $\alpha^\pm$ YSR peaks ($\pm 2.41\,\mathrm{mV}$) visible very close to the MSS by a slight shift of its low energy shoulder towards E_F, and the low intensity $\gamma^\pm$ YSR peaks ($\pm 2.33\,\mathrm{mV}$) appearing as shoulder-like features in between the $\beta^\pm$ and $\alpha^\pm$ YSR peaks. In contrast, the spectrum taken on the Ag atom (1) does not show any additional in-gap states except for the MSS (see also the same spectrum in Fig. S18F). Focusing on the bias voltage range of the $\beta^\pm$ YSR peak, the $\mathrm{d}I/\mathrm{d}V$ spectrum in Fig. S18F taken on the empty spot (4) of the corral containing the Fe atom reveals a peak approximately at the same bias voltage, though with a much smaller intensity. This peak is not visible in the two spectra (1,2) taken in the corral with the Ag atom. As described in the main text, we assign this peak to the $\beta^\pm$ YSR quantum projection.

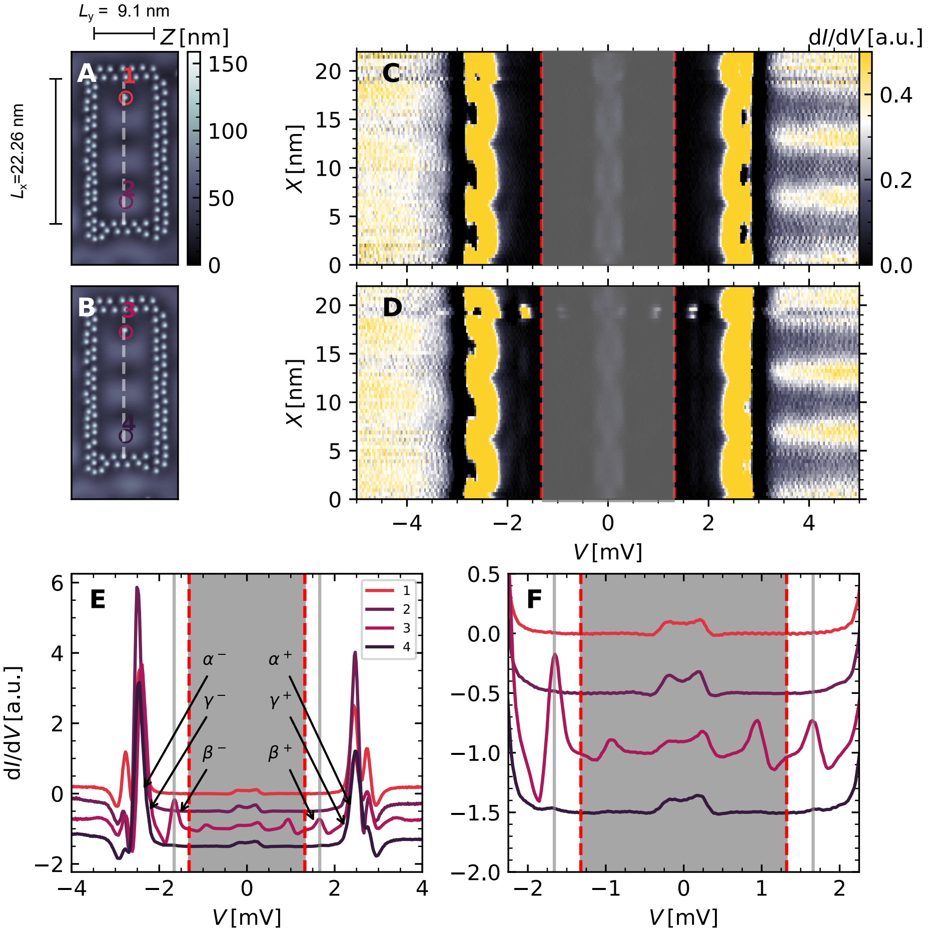

Supplementary Figure 18 | Extended data of the YSR quantum projection in a rectangular corral. (**A** and **B**) Same constant-current STM images of the Ag corrals as in Fig. 4 of the main text ($L_x = 22.26\,\mathrm{nm}$, $L_y = 9.1\,\mathrm{nm}$) with a Ag atom placed in the topmost quarter (A) and of the corral where the Ag was replaced with an Fe atom (B) ($V_{\mathrm{bias}} = -5\,\mathrm{mV}$, $I_{\mathrm{set}} = 1\,\mathrm{nA}$). (**C** and **D**) Same $\mathrm{d}I/\mathrm{d}V$ line profiles as in Fig. 4 of the main text taken along the dashed vertical lines in (A) and (B), respectively, but across the full bias range without cutting out the gray region between the red dashed vertical lines marking $\pm\Delta_{\mathrm{t}}/e$, and limited to a narrower range of $\mathrm{d}I/\mathrm{d}V$ values ($V_{\mathrm{stab}} = -5\,\mathrm{mV}$, $I_{\mathrm{stab}} = 1\,\mathrm{nA}$, $V_{\mathrm{mod}} = 50\,\mu\mathrm{V}$). (**E** and **F**) $\mathrm{d}I/\mathrm{d}V$ spectra taken on the Ag atom (at position 1 in panel A, red curve), on the empty spot of the corral with the Ag atom (at position 2 in panel A, purple curve), on the Fe atom (at position 3 in panel B, magenta curve), and on the empty spot of the corral with the Fe atom (at position 4 in panel B, black curve). YSR states are labelled with $\alpha^{\pm}$, $\beta^{\pm}$ and $\gamma^{\pm}$. The gray vertical lines mark the voltage of the $\beta^{\pm}$ YSR states. The red dashed vertical lines mark the voltage of the tip gap $\pm\frac{\Delta_{\mathrm{t}}}{e} = 1.32\,\mathrm{mV}$ ($V_{\mathrm{stab}} = -5\,\mathrm{mV}$, $I_{\mathrm{stab}} = 1\,\mathrm{nA}$, $V_{\mathrm{mod}} = 50\,\mu\mathrm{V}$, stabilized on outer wall Ag atom).

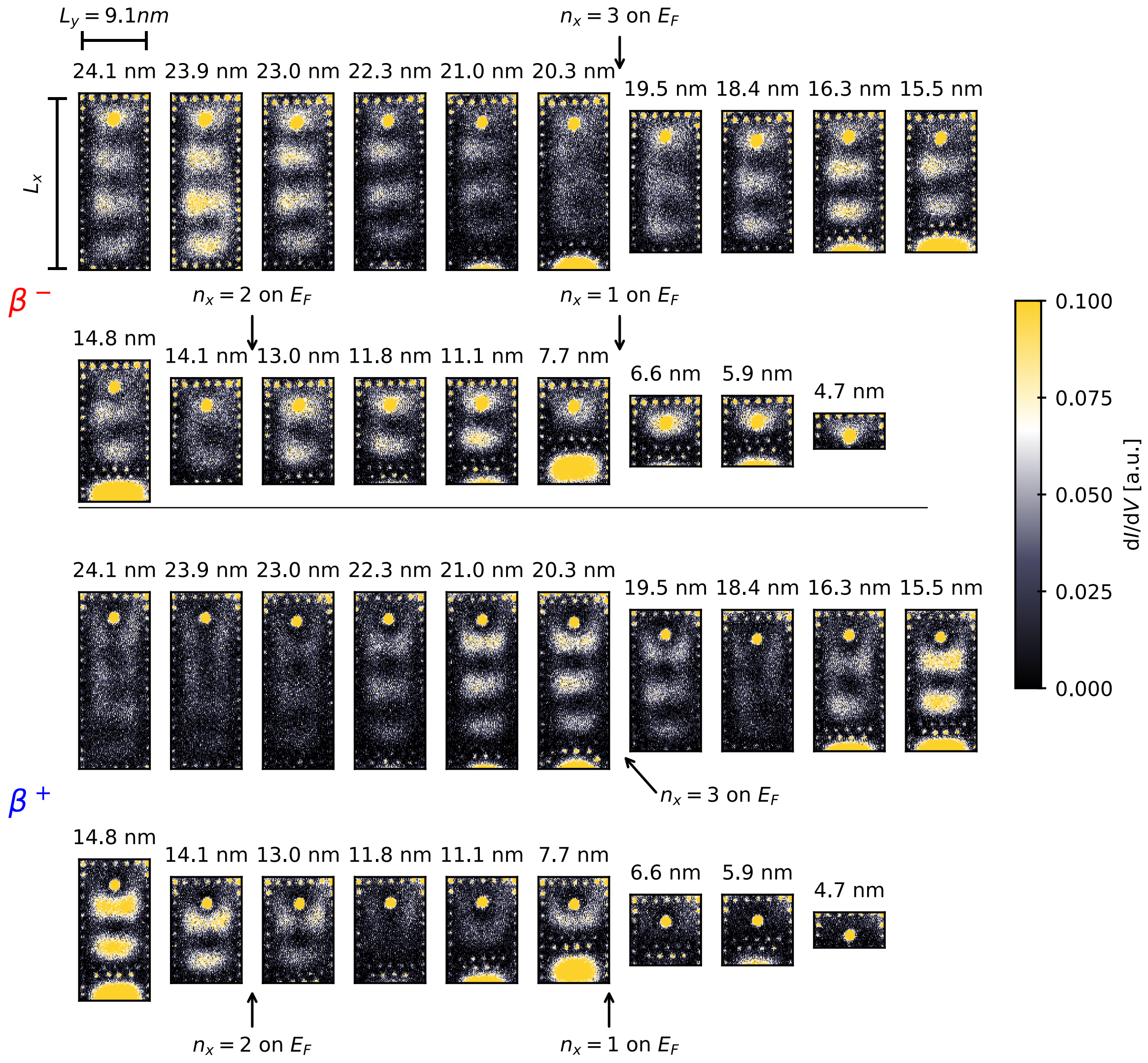

Supplementary Figure 19 | $\beta^{\pm}$ **YSR quantum projections of all rectangular corrals.** Experimental constant-height $\mathrm{d}I/\mathrm{d}V$ maps taken inside all corrals close to the bias corresponding to $-E_\beta$ (top two rows, $V_{\text{bias}} = -1.67\,\text{mV}$) and to $+E_\beta$ (bottom two rows, $V_{\text{bias}} = 1.67\,\text{mV}$, other parameters as in Figs. 5d to i of the main text). The lengths L_x are given above each panel and the widths are constant $L_y = 9.1\,\text{nm}$. The black arrows with annotations ($n_x = 3, 2, 1$ on E_F) mark the lengths L_x for which the respective eigenmode crosses E_F according to the fit done in Fig. S16.

Supplementary Note 6 Spatially resolved p-h asymmetry of the YSR quantum projection

In order to study the spatial distribution of the p-h asymmetry of the YSR quantum projection we calculate the asymmetry $\mathcal{A}$ from the experimental $\mathrm{d}I/\mathrm{d}V$ maps by

$$\mathcal{A} = \frac{(\mathrm{d}I/\mathrm{d}V)_{+E_\beta} - (\mathrm{d}I/\mathrm{d}V)_{-E_\beta}}{(\mathrm{d}I/\mathrm{d}V)_{+E_\beta} + (\mathrm{d}I/\mathrm{d}V)_{-E_\beta} + \varepsilon} \tag{41}$$

For the according asymmetries extracted from the calculated LDOS maps, $\mathrm{d}I/\mathrm{d}V$ is replaced by the calculated LDOS at the corresponding energies. Here, $\varepsilon = 0.1$ is a small offset in order to avoid strong noise in $\mathcal{A}$ at locations, where both, the p and the h contributions of the projection are close to zero. The resulting asymmetry maps for the three corrals in Fig. 5 of the main text are shown in Fig. S20. The following can be observed, both in the experiment (Figs. S20J to L) as well as in the calculations (Figs. S20S to U). For all three corrals, $\mathcal{A} < 0$ on the location of the Fe atom, corresponding to the predominant h character of the native YSR state of the Fe atom. For the corrals shown in Figs. S20A and C, with eigenmodes located well outside the gap, $\mathcal{A} < 0$ also at the maxima of the YSR quantum projections. In contrast, for the corral shown in Fig. S20B, where the $(3, 1)$ corral eigenmode in the absence of SC would be located very close to E_F, $\mathcal{A} > 0$ at the locations of the maxima of the YSR quantum projection. Consequently, for this case, the p-h asymmetry of the YSR quantum projection is reversed with respect to that of the native Fe YSR state. This can also be concluded from the analysis of the integrated intensities of the YSR quantum projections, as will be described in the following Section, and is shown in Fig. 5p,q of the main manuscript text.

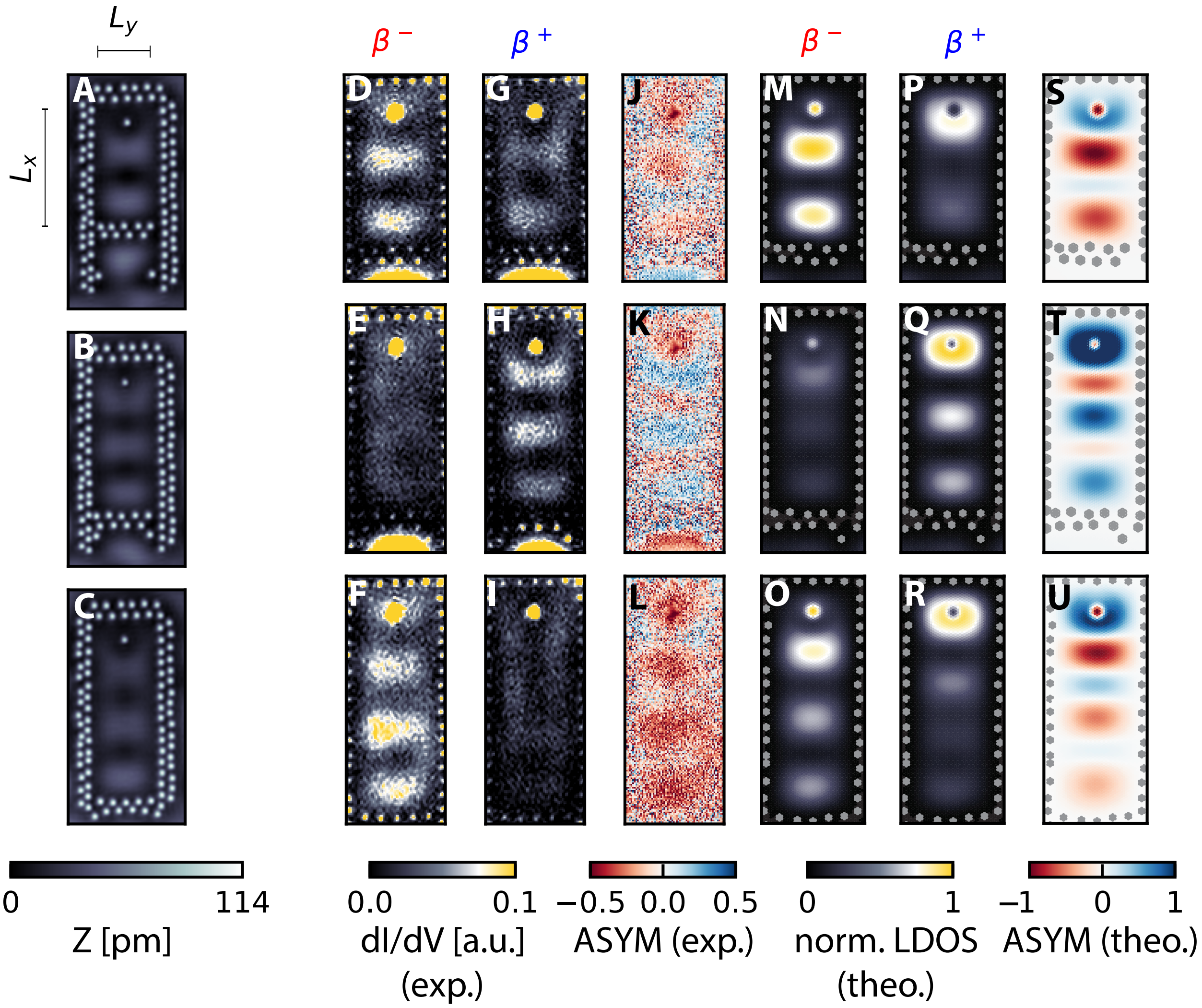

Supplementary Figure 20 | Spatially resolved p-h asymmetry of the YSR quantum projection. (**A** to **C**) Constant-current STM images of the same Ag corrals as in Fig. 5 of the main text including an Fe atom at the top. (**D** to **I** and **M** to **R**) Experimental constant-height $\mathrm{d}I/\mathrm{d}V$ maps (D to I) and simulated LDOS maps (M to R) taken inside all corrals close to the bias corresponding to $\pm E_\beta$ as indicated (same as in Fig. 5 of the main text). (**J** to **L** and **S** to **U**) Asymmetry maps (J to L) of the $\mathrm{d}I/\mathrm{d}V$ maps and (S to U) of the LDOS maps calculated as described in Supplementary Note 6.

Supplementary Note 7 Fitting procedures for the β YSR quantum projection

In order to extract the intensities of the $\beta^{\pm}$ YSR quantum projections as a function of corral length L_x, we used the following two different procedures, the first using the constant-height $\mathrm{d}I/\mathrm{d}V$ maps in Fig. S19, and the second using the $\mathrm{d}I/\mathrm{d}V$ line profiles given in the 2nd column of Fig. S17. Please note, that the intensities of the quantum projections corresponding to the $n_x = 1$ mode were excluded from this analysis, as they are too strongly spatially overlapping with the native Fe YSR state. For both procedures, the size L_x of each corral was determined by the distance along x between the maxima in the STM constant-current image of the Ag atoms in the inner wall at the top and the inner wall at the bottom (Fig. S22A, light blue horizontal lines). A height profile as in Fig. S22B was taken along the central longitudinal axis of each corral given by the vertical cyan dashed line in Fig. S22A. The position of the Fe atom in each corral was determined from the according maximum in the height profile, and an x interval of about $\pm 1.5\,\mathrm{nm}$ around this position, which is a bit larger than the spatial extents of the native $\beta^{\pm}$ YSR states (see Fig. S3), was defined (Fig. S22B, gray shaded area, and Figs. S21A,B, between the top two horizontal orange lines).

In the first procedure, we then analysed the constant-height $\mathrm{d}I/\mathrm{d}V$ maps in Fig. S19, as exemplarily shown in Fig. S21 for the $L_x = 24.1\,\mathrm{nm}$ corral containing a $(4, 1)$ eigenmode. To avoid the signal from the native YSR state of the Fe atom itself, we only use the lower portion of the $\mathrm{d}I/\mathrm{d}V$ map located inside the corral, but remove the upper portion around the Fe atom (see orange lines in Figs. S21A,B). This data can be seen in Fig. S21C for the β^- YSR quantum projection and in Fig. S21D for the β^+ YSR quantum projection. Afterwards, the data is averaged along the horizontal position (y) and plotted along the vertical position x as shown in Figs. S21E,F. These data points can be decently fitted to cosine functions $\cos(k^- x + \theta^-)$ for the β^- and $\cos(k^+ x + \theta^+)$ for the β^+ quantum projection intensities depicted by the solid red and blue lines, respectively. The good agreement between fit and data shows, that, while there is a difference in the wavenumbers k^- and k^+ of the p and h components of the YSR quantum projection, the phase shift $(\theta^+ - \theta^-)$ between the two components is constant. The latter hallmarks the long-range coherent nature of the YSR quantum projection in the rectangular quantum corral [6].

The resulting amplitudes of the fits represent the intensities of the $\beta^{\pm}$ YSR quantum projections obtained for this specific L_x, which are given in Fig. 5p of the main text as a function of L_x. In order to estimate the error, we did the same analysis with two horizontal distances between the two vertical orange lines in Figs. S21A,B, the first using the distance shown in the figure and the second using one third of this distance. The intensities plotted in Fig. 5p of the main manuscript are the mean values of these two intensities and the errors are given by their differences.

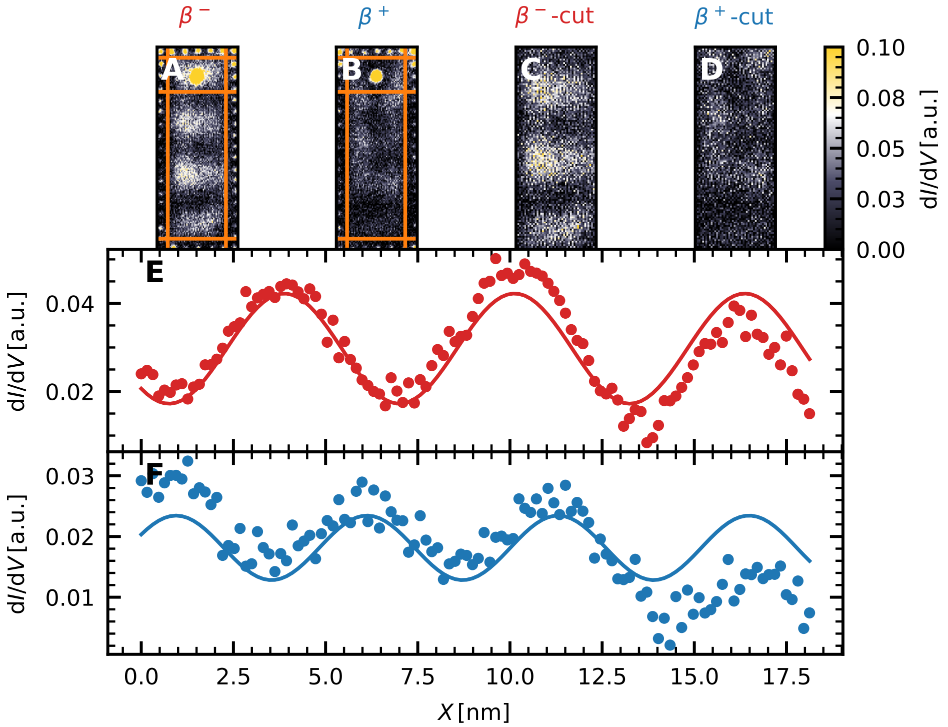

Supplementary Figure 21 | Analysis of the quantum projection using the $\mathrm{d}I/\mathrm{d}V$ maps. (**A** and **B**) Constant-height $\mathrm{d}I/\mathrm{d}V$-maps taken close to the bias voltages corresponding to $-E_\beta$ (A) and $+E_\beta$ (B) inside the $L_x = 24.1\,\mathrm{nm}$ corral with the Fe atom (same data as in Fig. S19). (**C** and **D**) Zoom into the bottom rectangular areas defined by the orange lines in panels (A) and (B), respectively. (**E** and **F**) $\mathrm{d}I/\mathrm{d}V$ signal (data points) as a function of vertical position (x) averaged along the width (horizontal position, y) of panels (C) and (D), respectively. The lines are fits of cosine functions to the data points as described in the text.

In order to cross check this result and to also extract the energies of the $\beta^\pm$ YSR quantum projections, we analyzed all the averaged $\mathrm{d}I/\mathrm{d}V$ line profiles given in the 2nd column of Fig. S17 as described in the following exemplarily for the corral of length $L_x = 23.91\,\mathrm{nm}$, see Fig. S22. A $\mathrm{d}I/\mathrm{d}V$ line profile as in Fig. S22C was taken along the central longitudinal axis of each corral given by the vertical cyan dashed line in Fig. S22A. Next, we used a $\mathrm{d}I/\mathrm{d}V$ spectrum which was acquired on the Fe atom in the same corral (Fig. S22D) to define an energy window around the native $\beta^\pm$ YSR states and corrected for a small bias offset in the measurement. To that end, we extracted the bias voltages $V_{\beta-}$ and $V_{\beta+}$ of the $\beta^\pm$ YSR peaks by fitting a Gaussian to the data points in a bias voltage interval around the peaks (Fig. S22D, vertical lines), as shown in Fig. S22F exemplarily for the β^- peak. We then calculated the bias offset $\Delta V = \frac{V_{\beta+}+V_{\beta-}}{2}$ for offset correction of all $\mathrm{d}I/\mathrm{d}V$ line profiles (Fig. S22C and 2nd column of Fig. S17). From these $\mathrm{d}I/\mathrm{d}V$ line profiles, we cut out the same x interval of points around the Fe atom determined above (Fig. S22C, gray

shaded area). For all the remaining points, e.g., the one marked by a black horizontal dashed line in Fig. S22C, we extracted the $\mathrm{d}I/\mathrm{d}V$ spectrum (Fig. S22E) and fitted a Gaussian to the β^- (Fig. S22G) and β^+ YSR quantum projection peaks (not shown), using only the data points defined by the same bias voltage interval mentioned above around the YSR peak in the $\mathrm{d}I/\mathrm{d}V$ spectrum taken on the Fe atom. From this fit, we extracted a value for the intensity and energy of the β^- YSR projection at this point in the particular corral. Note, that if this fitting procedure failed to converge, e.g., for positions around a nodal line of the projection, these points were omitted. Finally, all intensities and energies of these fits to the β^- and β^+ YSR projection peaks were separately averaged weighted by the errors of the individual Gaussian fits resulting in the average values of the intensities and energies of the β^- and β^+ YSR projections for the particular corral of length L_x. These averaged energies and intensities are shown in Figs. S23A,B as a function of L_x. As already visible in Fig. 4e of the main text for a particular corral, the bias voltages of the $\beta^\pm$ YSR projections are almost equal to the bias voltages corresponding to the energies $\pm E_\beta$ of the $\beta^\pm$ YSR states of the Fe atom for all the investigated corrals (cf. Figs. S23A and S24B). Nevertheless, there is a small systematic shift of the $\beta^\pm$ YSR projection bias voltage towards the gap edge with respect to $\pm E_\beta$ by less then $100\,\mu\mathrm{V}$, which is also visible in Figs. S22F,G. It is most probably caused by the overlap of the YSR projection peak with the shoulder of the very intense MSS (see, e.g., Fig. 3k of the main text). Most importantly, the intensities of the $\beta^\pm$ YSR quantum projections extracted from this second procedure (Fig. S23B) show the maxima and minima at the same L_x values as when using the first procedure (cf. Fig. 5p of the main text), even though the relative heights of the maxima slightly differ.

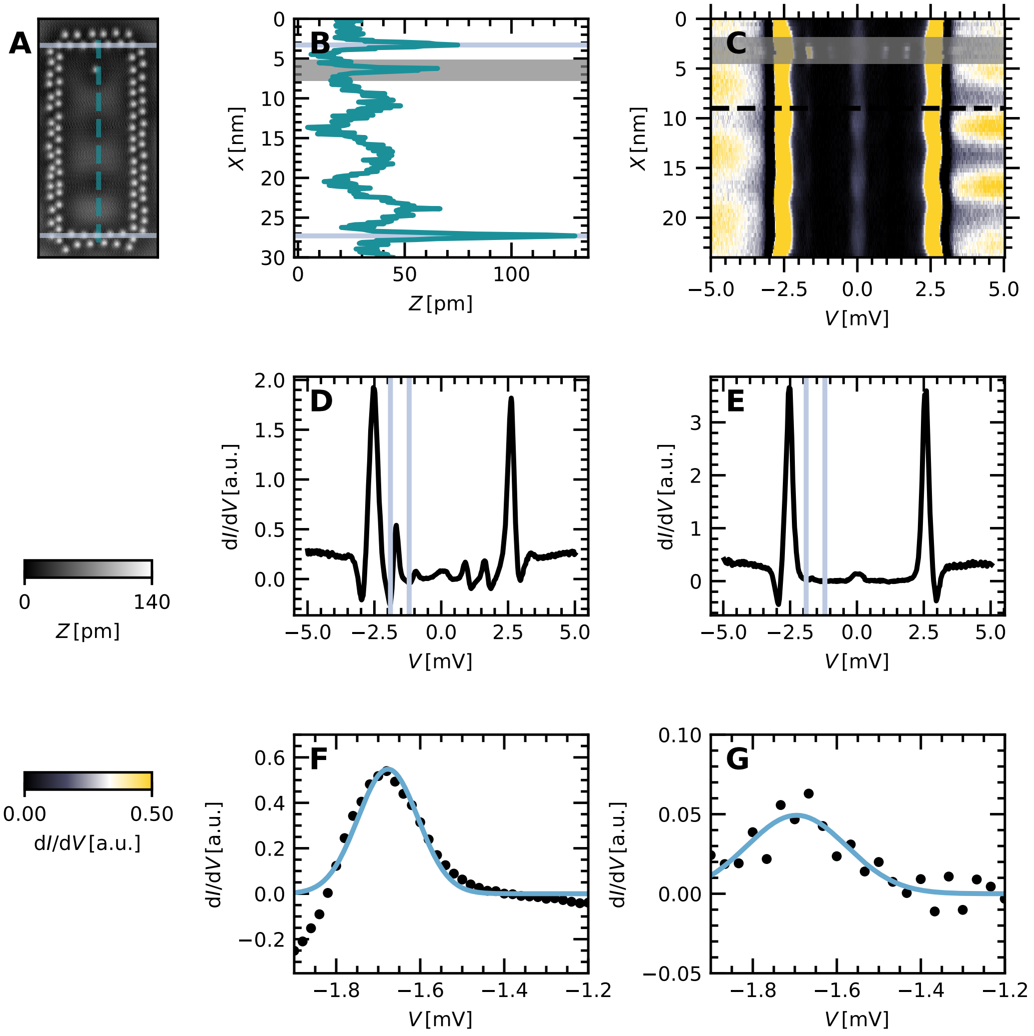

Supplementary Figure 22 | Analysis of the quantum projection using $\mathrm{d}I/\mathrm{d}V$ line profiles. (**A**) Constant-current STM image of the Ag corral with width $L_y = 9.1\,\mathrm{nm}$ and length $L_x = 23.91\,\mathrm{nm}$, including a Fe atom at the top (same data as in Fig. 5c of the main text). (**B**) Height profile along the central longitudinal axis of the corral indicated by the cyan dashed line in (A). The horizontal light blue lines in (A) and (B) mark the positions of the top and bottom inner Ag rows, which determine the corral length L_x. (**C**) $\mathrm{d}I/\mathrm{d}V$ line profile along the same cyan dashed line in (A) (same data as in Fig. S17, but upside down). The gray shaded areas around the position of the Fe atom in (B) and (C) depict the x range which is cut out from the $\mathrm{d}I/\mathrm{d}V$ line profile for the analysis of the averaged YSR quantum projection energy and intensity (see Supplementary Note 7). Its size corresponds to the spatial extent of the native β YSR state (see Figs. S3G and H). (**D** and **E**) $\mathrm{d}I/\mathrm{d}V$ spectra at the location of the Fe atom (D) and at an exemplary position x on the central longitudinal axis inside the corral (E) given by the black dashed line in (C). (**F** and **G**) $\mathrm{d}I/\mathrm{d}V$ data points in the intervals marked by the vertical lines in (D) and (E), respectively, which are included for the Gaussian fits of the β^- peaks given by the blue lines. These fits are used to extract the average energies and intensities of the β^- YSR state (F) and quantum projection (G) as described in Supplementary Note 7.

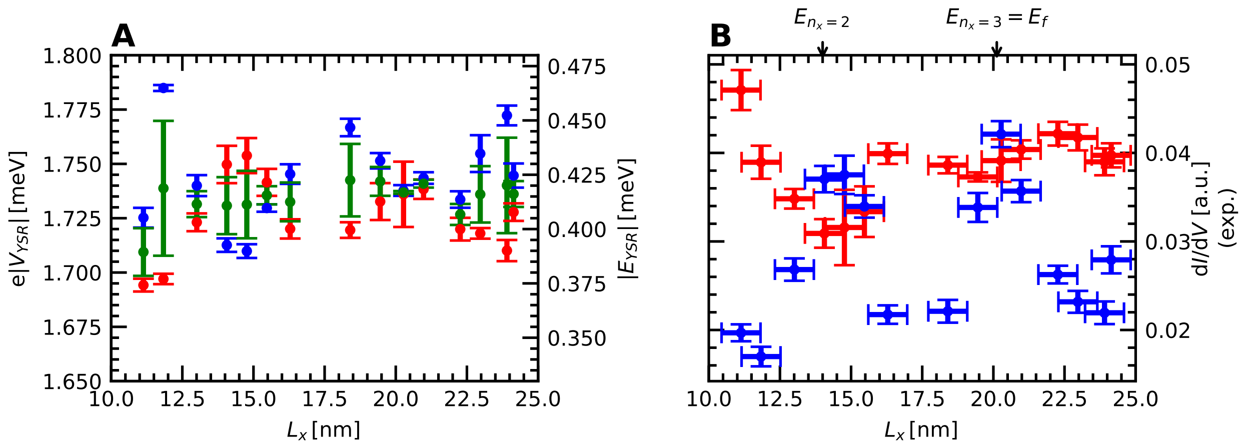

Supplementary Figure 23 | Energies and intensitites of the $\beta^{\pm}$ YSR quantum projections. (**A**) Average values of the energies of the β^{-} (red) and β^{+} (blue) YSR quantum projections as a function of corral length L_x. The green data points are the mean values of the β^{-} and β^{+} YSR quantum projection energies. (**B**) Intensities of the β^{-} (red) and β^{+} (blue) YSR quantum projections extracted from the second procedure as described in Supplementary Note 7. The horizontal errors are defined by the deviation of the short-corral side's inner wall atoms from a straight line. The vertical errors are standard deviations of the weighted averages as described in Supplementary Note 7.

Supplementary Note 8 $\beta^{\pm}$ YSR states on Fe atoms in the corral

For each corral of length L_x we also acquired a $\mathrm{d}I/\mathrm{d}V$ spectrum on top of the Fe atom inside the corral. To this end, before taking each spectrum, the tip is stabilized on the same Ag atom in the outer wall of the corral. The spectra are shown in Fig. S24A as a waterfall plot for all the different corrals as a function of L_x. Whenever a corral eigenmode approaches E_F, a MSS splits off from the substrate coherence peaks, and merges with them when the eigenmode shifts far away from the gap region [2]. However, at the same time, the energy of the $\beta^{\pm}$ peaks on the Fe stays largely constant. Figure S24B shows the average voltage $|V_\mathrm{YSR}| = \frac{V_{\beta^+} - V_{\beta^-}}{2}$ (and corresponding average energy $|E_\mathrm{YSR}|$) of the $\beta^{\pm}$ peaks extracted by fitting to Gaussians. Again, it can be seen that the energies of the β YSR peaks on the Fe in the corral stay largely constant, independent of the corral length L_x. This insensitivity of the energy of the β YSR peak on the presence or absence of the corral eigenmode alludes to a dominant coupling of the Fe $3d$ orbitals to the bulk Ag electron states and a much weaker coupling to the Ag surface state.

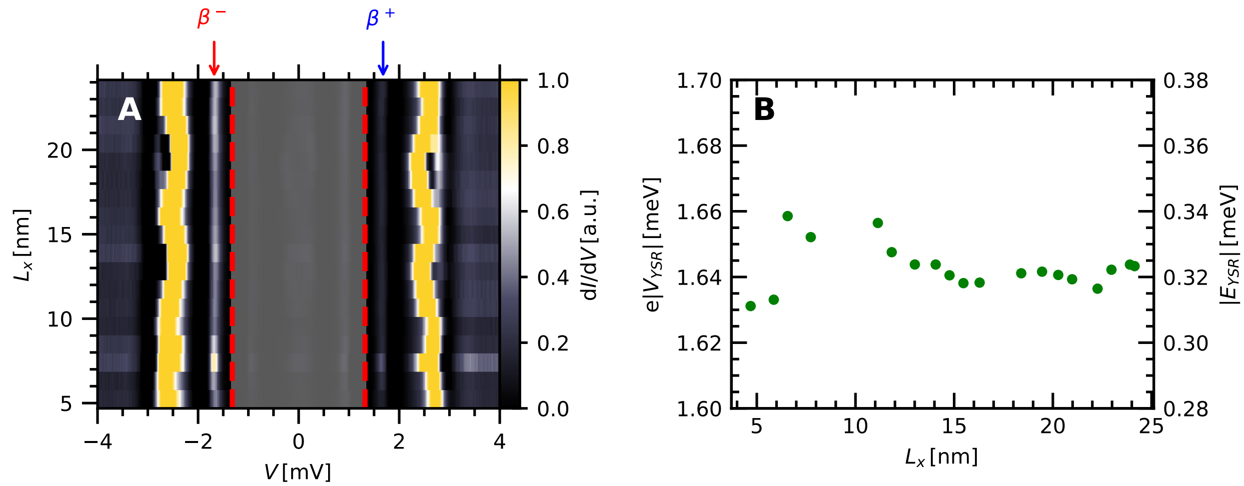

Supplementary Figure 24 | Fe YSR state evolution with increasing corral length.
(**A**) $\mathrm{d}I/\mathrm{d}V$ spectra taken on the Fe atom inside the corrals with lengths of $L_x = 4.7\,\mathrm{nm}$ to $L_x = 24.1\,\mathrm{nm}$ (constant width $L_y = 9.1\,\mathrm{nm}$, $V_{\mathrm{stab}} = -5\,\mathrm{mV}$, $I_{\mathrm{stab}} = 1\,\mathrm{nA}$, $V_{\mathrm{mod}} = 50\,\mu\mathrm{V}$; note, that for all these measurements, the tip was stabilized on the same Ag atom in the outer corral wall). The colored arrows indicate the β^- and β^+ YSR peaks and the red dashed vertical lines indicate $\pm\frac{\Delta_t}{e}$. (**B**) Average β YSR-state energies on Fe extracted by Gaussian fits to the β^- and β^+ peaks from (A) (see Supplementary Note 8).

Supplementary Note 9 Effective model and analytical results

In this section, we show that, despite the complexity of the experimental setup, the qualitative physics can be captured by an effective model that is consistent with the results of the semi-infinite 3D tight-binding model in Supplementary Note 3 and generates a more qualitative understanding. To this end, we describe the corral mode as a Machida Shibata state (MSS), i.e., a non-interacting spin-degenerate level that acquires proximity superconductivity [2], and the YSR state by a non-interacting Anderson impurity which breaks time-reversal symmetry [7]. The bulk superconductor mediates a coupling between the two states, which sensitively depends on their real-space distance. We consider the Hamiltonian

$$\begin{aligned} H_{\mathrm{M}} =& H_{\mathrm{MS}} + H_{\mathrm{YSR}} + H_{\mathrm{SC}}, \\ H_{\mathrm{SC}} =& \sum_{\mathbf{k},\sigma} \epsilon_{\mathbf{k}} c^{\dagger}_{\mathbf{k},\sigma} c_{\mathbf{k},\sigma} - \Delta \sum_{\mathbf{k}} \left(c^{\dagger}_{\mathbf{k}\uparrow} c^{\dagger}_{-\mathbf{k}\downarrow} + c_{-\mathbf{k}\downarrow} c_{\mathbf{k}\uparrow} \right), \\ H_{\mathrm{YSR}} =& \sum_{\sigma} (\sigma J + U) \tilde{d}_{\sigma}{}^{\dagger} \tilde{d}_{\sigma} + \sum_{\mathbf{k},\sigma} \tilde{V}(\mathbf{k}) c^{\dagger}_{\mathbf{k},\sigma} \tilde{d}_{\sigma} + \tilde{V}^{*}(\mathbf{k}) \tilde{d}^{\dagger}_{\sigma} c_{\mathbf{k},\sigma}, \\ H_{\mathrm{MS}} =& E_{\mathrm{R}} \sum_{\sigma} d^{\dagger}_{\sigma} d_{\sigma} + \sum_{\mathbf{k},\sigma} V(\mathbf{k}) c^{\dagger}_{\mathbf{k},\sigma} d_{\sigma} + V^{*}(\mathbf{k}) d^{\dagger}_{\sigma} c_{\mathbf{k},\sigma}. \end{aligned} \tag{42}$$

Here, the operators d_σ and $\tilde{d}_\sigma$ refer to the MSS and the YSR level, respectively, and $\epsilon_{\mathbf{k}} = \mathbf{k}^2/2m - E_{\mathrm{F}}$ is the dispersion relation of the $3D$ bulk superconductor with Fermi energy E_{F}. The coupling constants $V(\mathbf{k}) = V$ and $\tilde{V}(\mathbf{k}) = \tilde{V} e^{i\mathbf{kR}}$ describe a MSS localized at the origin and a YSR state located at position **R**, respectively. In the limit $\tilde{V} = 0$, we obtain the model used for empty corrals in Ref. 2. In the experiment, the length of **R** qualitatively is the distance of the Fe atom to the position where the corral state dominantly couples to the bulk [2]. Yet, because of the technically necessary approximation of a localized corral mode, there is no one-to-one correspondence to the experimental geometry. Because the MSSs of different quantum numbers are energetically sufficiently separated, we constrain the model to one of these states and describe its crossing of the Fermi energy E_{F} as in Fig. 5 of the main text. To this end, we integrate out the bulk modes and find the YSR quantum projection density of states when evaluating the DOS of the corral state at the energy of the YSR state $\mathrm{DOS(E)} = -\frac{1}{\pi} \lim_{\eta\to 0} \mathrm{Im} \left(G_{d_\uparrow d^{\dagger}_\uparrow}(E + i\eta) + G_{d_\downarrow d^{\dagger}_\downarrow}(E + i\eta) \right)$, where η is a small positive number. Using the Green's functions equation of motion technique [8], we find the energy-dependent Green's function to be

$$\begin{aligned} G_{d_\uparrow d^{\dagger}_\uparrow}(E) =& \frac{2\delta\epsilon\zeta + \zeta^2\mu + \delta^2\xi + \nu(\epsilon^2 - \mu\xi)}{D}, \\ D =& -\delta^4 + 2\beta\gamma\epsilon\zeta - \beta^2\zeta^2 + \alpha\zeta^2\mu + \alpha\epsilon^2\nu + \beta^2\nu\xi - \alpha\mu\nu\xi \\ & + \delta^2(2\gamma\epsilon + 2\beta\zeta + \mu\nu + \alpha\xi) + 2\delta(\alpha\epsilon\zeta + \gamma\zeta\mu + \beta\epsilon\nu + \beta\gamma\xi) + \gamma^2(-\epsilon^2 + \mu\xi), \end{aligned} \tag{43}$$

and $G_{d_\downarrow d_\downarrow^\dagger}(E)$ is obtained by changing $J \to -J$. Above, we have defined

$$\alpha = E - E_R + \frac{\Gamma E}{\sqrt{\Delta^2 - E^2}}, \beta = -\frac{\Delta\Gamma}{\sqrt{\Delta^2 - E^2}},$$
$$\gamma = -\frac{\sqrt{\Gamma\tilde{\Gamma}}}{\sqrt{\Delta^2 - E^2}} \frac{e^{-\frac{\sqrt{\Delta^2 - E^2}R}{\upsilon_{\mathrm{F}}}}}{k_{\mathrm{F}}R} \left(\sin(k_{\mathrm{F}}R)E + \sqrt{\Delta^2 - E^2}\cos(k_{\mathrm{F}}R)\right),$$
$$\delta = -\frac{\Delta\sqrt{\Gamma\tilde{\Gamma}}}{\sqrt{\Delta^2 - E^2}} \frac{\sin(k_{\mathrm{F}}R)e^{-\frac{\sqrt{\Delta^2 - E^2}R}{\upsilon_{\mathrm{F}}}}}{k_{\mathrm{F}}R},$$
$$\epsilon = -\frac{\sqrt{\Gamma\tilde{\Gamma}}}{\sqrt{\Delta^2 - E^2}} \frac{e^{-\frac{\sqrt{\Delta^2 - E^2}R}{\upsilon_{\mathrm{F}}}}}{k_{\mathrm{F}}R} \left(\sin(k_{\mathrm{F}}R)E - \sqrt{\Delta^2 - E^2}\cos(k_{\mathrm{F}}R)\right), \zeta = -\frac{\Delta\tilde{\Gamma}}{\sqrt{\Delta^2 - E^2}},$$
$$\mu = E + E_R + \frac{\Gamma E}{\sqrt{\Delta^2 - E^2}}, \nu = E - J - U + \frac{\tilde{\Gamma} E}{\sqrt{\Delta^2 - E^2}}, \xi = E - J + U + \frac{\tilde{\Gamma} E}{\sqrt{\Delta^2 - E^2}}, \tag{44}$$

where we have assumed a linearized dispersion of the bulk near the Fermi energy $\epsilon_k \approx \upsilon_{\mathrm{F}}(k - k_{\mathrm{F}})$, where $\upsilon_{\mathrm{F}}, k_{\mathrm{F}}$ are the Fermi velocity and wavevector, respectively. The scattering strengths are defined by $\Gamma = \pi D(E_{\mathrm{F}})V^2$ and $\tilde{\Gamma} = \pi D(E_{\mathrm{F}})\tilde{V}^2$, where $D(E_{\mathrm{F}})$ is the normal-state density of the bulk. As seen from Eq. (43), the MSS and the YSR state are coupled via the bulk and the coupling strength scales with powers of $\sqrt{\Gamma\tilde{\Gamma}}/k_{\mathrm{F}}R$. The exponential decay for the parameters γ, δ, ϵ in dependence on the distance R in Eq. (44) is suppressed for corral lengths that we consider ($\xi \sim 400$ nm for proximity superconducting Ag)[2]. The denominator D defined in Eq. (43) simplifies to $D \approx -\left(-\beta^2 + \alpha\mu\right)\left(-\zeta^2 + \nu\xi\right)$ in the small hybridization limit $R \gg \lambda_{\mathrm{F}}$, where λ_{F} is the Fermi wavelength of the proximitized superconducting Ag bulk. In this limit, the MSS and the YSR state are weakly coupled to each other, irrespective of the coupling of the MSS to the bulk. The conditions $\left(-\beta^2 + \alpha\mu\right) = 0$ and $\left(-\zeta^2 + \nu\xi\right) = 0$ determine the energies of the uncoupled MSS and YSR state (for $J \gg \Delta$) in this limit, respectively. In the experiment of the main text, the relevant limit is the small-hybridization regime (between MSS and YSR state) where we do not observe significant fluctuations in the energetic position of the YSR state as the corral eigenmodes are tuned through the Fermi energy, see Fig. S23. Also, the degenerate MSS levels practically do not split due to the weak hybridization. Yet, there remains a finite density of states at the YSR energy in the corral, corresponding to the YSR quantum projection. We find that this regime in the model in Eq. (42) requires $R \gg \lambda_{\mathrm{F}}$, which is consistent with the length scales of the studied corrals (about 10 nm) compared to λ_{F} of the Ag bulk, estimated to be ~ 0.5 nm [9]. The small wavelength λ_{F} is, also, related to the strong localization of the β YSR states if the MSS's energy is far away from E_{F}, see Fig. 2 of the main text.

The particle-hole weight of the YSR quantum projection is determined by Eq. (43) and the indirect coupling expressions γ, δ, ϵ in Eq. (44). We note that the spatial period of the oscillations in the quantum projections of the $\beta^{\pm}$ peak is $\frac{\lambda_{\mathrm{F}}}{2}$, because γ, δ, and ϵ always appear in pairs in Eq. (43). The phase of these oscillations depends on several parameters such as the energetic position of the

YSR state or MSS. Thus, as the corral eigenmodes are tuned through the Fermi energy, included in our model by changing E_R, the particle-hole ratio of the YSR quantum projection oscillates. This effect originates from the bulk particle-hole oscillations of the YSR state [6]. Yet, the extra corral levels shift the phase and intensity of these oscillations.

To generally analyze the change of the particle-hole polarization, we focus on the asymmetry $\mathcal{A} := \mathrm{DOS}(\beta^+) - \mathrm{DOS}(\beta^-)$ of the YSR quantum projection of the $\beta^\pm$ peaks while tuning the corral modes through the Fermi energy, see Fig. S25. By exhaustive numerical search in the (J, U, R) parameter space, we find that $\mathcal{A}$ vanishes up to two times in the parameter range $E_R = 2\Delta \to -2\Delta$ for a fixed hybridization strength $\Gamma = \Delta$, see Fig. S25. We can therefore classify four scenarios by counting the number of crossings of the relative intensity of the quantum projection peaks in the range $E_R = 2\Gamma = 2\Delta \to -2\Delta$ and accounting for the sign of its value, i.e., $\mathrm{sgn}(\mathcal{A})$ at $E_R = -2\Delta$. Firstly, the asymmetry $\mathcal{A}$ vanishes twice while tuning the corral mode's energy E_R through the superconducting gap. Typically, the β^+ and β^- peaks run through a global maximum and a local minimum, respectively, see Fig. S25A. This case largely resembles the experimental findings and the semi-infinite 3D tight-binding model simulations shown in Figs. 5p,q of the main text, where the p-h composition of the YSR quantum projection is inverted with respect to that of the native YSR state when $E_R \approx 0$. We additionally check the influence of weakening the MSS hybridization to the bulk, Γ, with the MSS energy still not crossing the YSR energy (see Fig. S26) and find qualitative agreement with the 3D tight-binding model of Supplementary Note 3. Secondly, $\mathcal{A}$ vanishes twice but now the β^- peak runs through a global maximum and the β^+ peak through a local minimum, see Fig. S25B (non-inverted). Thirdly, if the intensity of the β^+ peak is dominant and increases first upon tuning E_R, the quantum projection peaks are qualitatively non-inverted with respect to the particle-hole asymmetry of the MSS, see example in Fig. S25C. This is the expected behaviour for a single particle-polarized level hybridizing first with the β^+ and afterwards with the β^- YSR state when tuned through the superconducting gap. Fourthly, if the intensity of the β^- peak is dominant first and also responds first when E_R is tuned through the gap, the quantum projection peaks are qualitatively inverted with respect to the p-h asymmetry of the MSS (not shown). This is expected for a hole-polarized level tuning through the Fermi level.

We note that the asymmetry $\mathcal{A}$ of the $\beta^\pm$ YSR quantum projection peaks strongly depends on the distance R between the YSR state and the MSS, see Eq. (43), pictured in the changes between Figs. S25D and E. Yet, we observe some universal characteristics. First, the change of the electronic ground state parity (red line in Figs. S25D,E) of the YSR state inverts the sign of $\mathcal{A}$ at $E_R = -2\Delta$ and, thus, changes the non-inverted to the inverted behaviour (and vice-versa). The influence of R on the $\beta^\pm$ asymmetry, $\mathcal{A}$, is shown in Figs. S25F,G.

While, ultimately, the Green's function in Eq. (43) encapsulates the full behavior of the system, qualitative insights can be gained by recalling that the particle-hole asymmetry in the model in Eq. (42) originates from the bulk particle-hole oscillations of the YSR state [6]. These oscillations' periodicity, $\lambda_\mathrm{F}/2$, matches the behavior shown in Figs. S25F,G. Yet, the presence of the MSS then introduces a shift in these oscillations which depends on the energetic position of

the MSS (compare Fig. S25F to Fig. S25G). Note that although the numerator in Eq. (43) does not explicitly depend on E_R, there are terms in the denominator that involve both α, which depends on E_R, and the coupling functions γ, δ, and ϵ.

While in our calculations, we focus on large effective distances R between the MSS and the YSR state, taking $R \to 0$ instead leads to a strong coupling contrary to the experimental data. To this end, we employ the local value of the YSR Green's function instead of the large R limit [10]. We remark that the four characteristic cases still appear in that limit, yet with different p-h profiles in dependence on E_R.

The minimal model, introduced in Eq. (43), offers a simplified but fundamental description of the physics observed in the experiment. As we have shown, the YSR quantum projection characteristics depend on the bulk parameters, the effective distance R and the properties of the corral modes. Additionally, the model predicts the scenarios with an inverted and a non-inverted YSR quantum projection LDOS for a different range of experimental parameters.

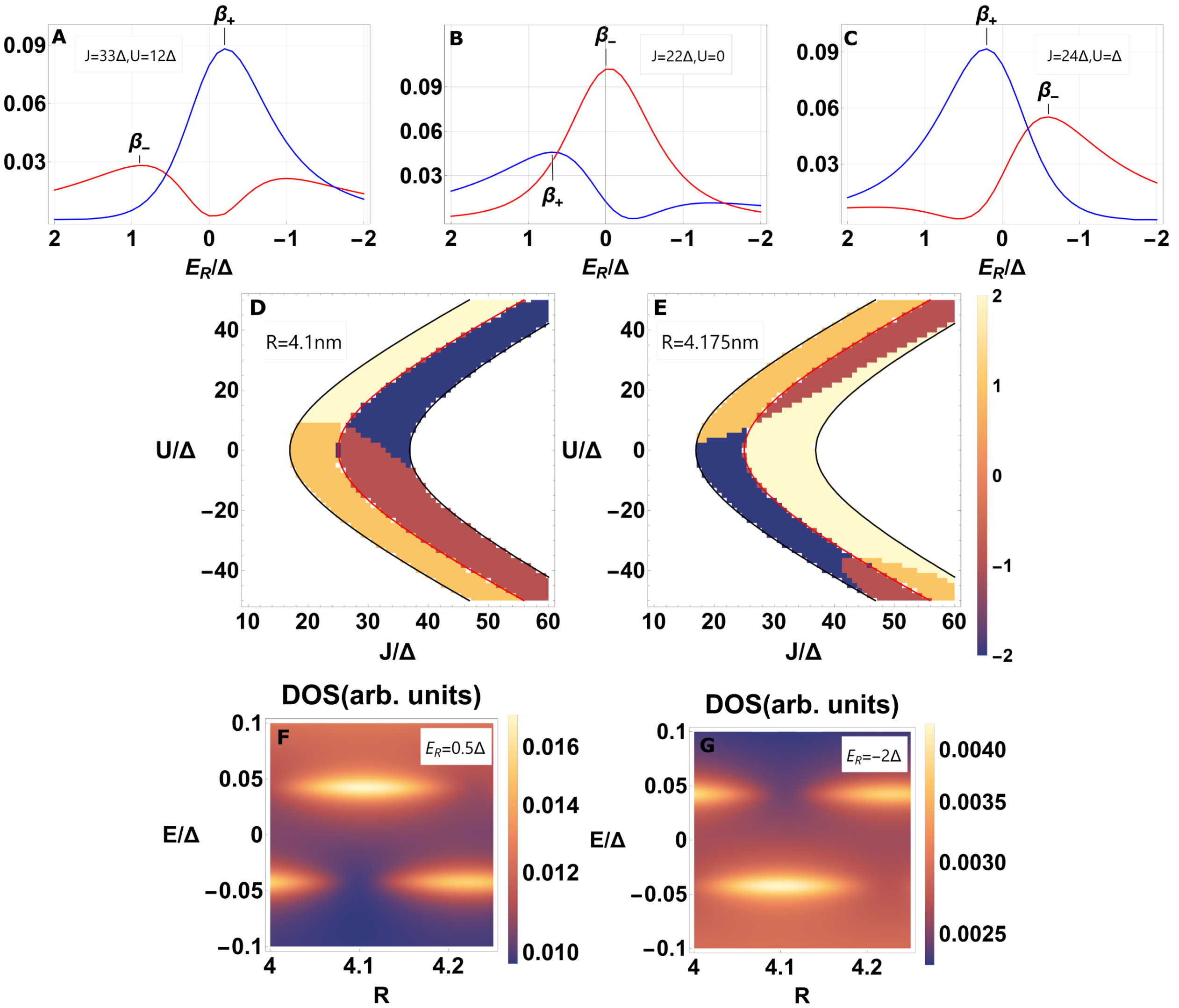

Supplementary Figure 25 | Characteristic scenarios of the p-h composition of the YSR quantum projection. (**A**) Double crossing projection-DOS plot resembling the experiment as well as the semi-infinite 3D tight-binding model simulations ($J = 33\Delta$ and $U = 12\Delta$). (**B**) Double crossing projection-DOS where the $\beta^{\pm}$ projections are qualitatively inverted compared to (A) ($J = 22\Delta$ and $U = 0$). (**C**) Single crossing projection-DOS plot showing the intensities of the projection's DOS peaks non-inverted with respect to the particle-hole polarization of the MSS ($J = 24\Delta$ and $U = \Delta$). (**D**) Characteristic parameter regimes, described by the number of crossings of the intensity of the β^+ and β^- peaks for E_R tuned from -2Δ to 2Δ multiplied by the sgn($\mathcal{A}$), calculated at $E_R = -2\Delta$ and $R = 4.1$ nm. The red line denotes the phase transition $E_{\text{YSR}} = 0$ while the black lines are the boundaries of the region $|E_{\text{YSR}}| \leq 0.37\Delta$. (**E**) Same as (D) but for $R = 4.175$ nm. (**F**) DOS as a function of the energy E and effective distance R, within one spatial period. The plot shows the DOS oscillations of the β^+ and β^- peaks in dependence on both variables. We consider $E_R = 0.5\Delta$ and a broadening $\eta = 0.01\Delta$. (**G**) Same as (F) but $E_R = -2\Delta$. In the above, we have taken $\Gamma = \Delta$, $\tilde{\Gamma} = 25\Delta$ and $\lambda_F = 0.5$ nm. We have chosen $R = 4.1$ nm for (C) and (D), and $R = 4.175$ nm for (A), (B) and (E). These results are sensitive to changes in R.

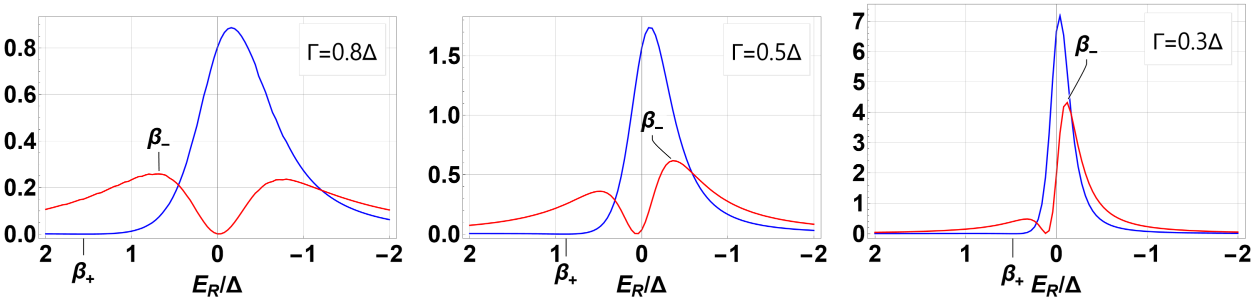

Supplementary Figure 26 | YSR quantum projection profiles for different MSS hybridization strengths to the bulk, Γ**.** β^{+}, β^{-} YSR quantum projection peaks evolution (blue and red curves, respectively) while the energy of the corral level, E_{R}, is tuned through the Fermi energy. The parameters $J = 33\Delta$, $U = 12\Delta$, $R = 4.175$ nm and $\eta = 10^{-4}\Delta$ are considered.

Supplementary References

1. Ruby, M., Peng, Y., von Oppen, F., Heinrich, B. W. & Franke, K. J. Orbital picture of Yu-Shiba-Rusinov multiplets. *Phys. Rev. Lett.* **117**, 186801 (2016).

2. Schneider, L. *et al.* Proximity superconductivity in atom-by-atom crafted quantum dots. *Nature* **621**, 60–65 (2023).

3. Grothe, S. *et al.* Quantifying many-body effects by high-resolution Fourier transform scanning tunneling spectroscopy. *Phys. Rev. Lett.* **111**, 246804 (2013).

4. Sancho, M. P. L., Sancho, J. M. L., Sancho, J. M. L. & Rubio, J. Highly convergent schemes for the calculation of bulk and surface Green functions. *Journal of Physics F: Metal Physics* **15**, 851 (1985).

5. Balatsky, A. V., Vekhter, I. & Zhu, J. X. Impurity-induced states in conventional and unconventional superconductors. *Reviews of Modern Physics* **78**, 373–433 (2006).

6. Ménard, G. C. *et al.* Coherent long-range magnetic bound states in a superconductor. *Nature Physics* **11**, 1013–1016 (2015).

7. Huang, H. *et al.* Quantum phase transitions and the role of impurity-substrate hybridization in Yu-Shiba-Rusinov states. *Communications Physics* **3**, 199 (2020).

8. Villas, A. *et al.* Interplay between Yu-Shiba-Rusinov states and multiple Andreev reflections. *Phys. Rev. B* **101**, 235445 (2020).

9. Mitchell, J. W. & Goodrich, R. G. Fermi velocities in silver: surface Landau-level resonances. *Phys. Rev. B* **32**, 4969–4976 (1985).

10. Mier, C., Choi, D.-J. & Lorente, N. Calculations of in-gap states of ferromagnetic spin chains on s-wave wide-band superconductors. *Phys. Rev. B* **104**, 245415 (2021).
